# Magmatic record of India-Asia collision

**DOI:** 10.1038/srep14289

**Published:** 2015-09-23

**Authors:** Di-Cheng Zhu, Qing Wang, Zhi-Dan Zhao, Sun-Lin Chung, Peter A. Cawood, Yaoling Niu, Sheng-Ao Liu, Fu-Yuan Wu, Xuan-Xue Mo

**Affiliations:** 1 State Key Laboratory of Geological Processes and Mineral Resources, and School of Earth Science and Resources, China University of Geosciences, Beijing 100083, China; 2 Institute of Earth Sciences, Academia Sinica, Taipei 11529, Taiwan; 3 Department of Geosciences, National Taiwan University, Taipei 10617, Taiwan; 4 Department of Earth Sciences, University of St Andrews, North Street, St Andrews KY16 9AL, UK; 5 Centre for Exploration Targeting, School of Earth and Environment, University of Western Australia, 35 Stirling Hwy., Crawley WA, 6009, Australia; 6 Department of Earth Sciences, Durham University, Durham DH1 3LE, UK; 7 Institute of Geology and Geophysics, Chinese Academy of Sciences, Beijing 100029, China

## Abstract

New geochronological and geochemical data on magmatic activity from the India-Asia collision zone enables recognition of a distinct magmatic flare-up event that we ascribe to slab breakoff. This tie-point in the collisional record can be used to back-date to the time of initial impingement of the Indian continent with the Asian margin. Continental arc magmatism in southern Tibet during 80–40 Ma migrated from south to north and then back to south with significant mantle input at 70–43 Ma. A pronounced flare up in magmatic intensity (including ignimbrite and mafic rock) at ca. 52–51 Ma corresponds to a sudden decrease in the India-Asia convergence rate. Geological and geochemical data are consistent with mantle input controlled by slab rollback from ca. 70 Ma and slab breakoff at ca. 53 Ma. We propose that the slowdown of the Indian plate at ca. 51 Ma is largely the consequence of slab breakoff of the subducting Neo-Tethyan oceanic lithosphere, rather than the onset of the India-Asia collision as traditionally interpreted, implying that the initial India-Asia collision commenced earlier, likely at ca. 55 Ma.

Continental collision is a dramatic expression of the dynamic nature of the Earth and has long-term impacts on atmosphere and ocean circulation patterns, and on the development and stability of the continental lithosphere. One of the most prominent collisions today is the ongoing interaction between the Indian and Asian continents. However, the timing of the initial India-Asia collision remains uncertain with suggestions ranging from 70 to 34 Ma^1^,^2^,^3^,^4^,^5^,^6^,^7^,^8^,^9^. This uncertainty reflects in part the differing approaches used to define collision.

Continental collision is the natural consequence of plate tectonics involving oceans opening and closing and is driven by a variety of forces most notably the descent of oceanic lithosphere at subduction zones (i.e. slab pull)^10^,^11^. Such understanding, together with the primary mechanisms of magma generation (i.e., adding fluids, increasing temperature, and decreasing pressure), allows us to place constraints on the relationship between collisional processes and magmatic responses (Fig. 1).

**(1) Initial collision,** takes place at the initial contact of crust between two continents (Fig. 1a). At this stage, oceanic lithosphere continues to subduct and dehydrates (releasing fluids), resulting in the generation of normal continental arc magmatism dominated by andesitic rocks as seen in the Andes. **(2) Ongoing collision**, involves the thin passive continental lithosphere being dragged by the subducting oceanic lithosphere (slab pull) (Fig. 1b). Minor syn-collisional magmatism during this stage is mostly peraluminous and derived from a metapelite-dominated source within the middle-upper crust due to reduced dehydration of the subducting oceanic lithosphere (releasing fluids), convective heat from small-scale mantle flow^12^, and shear heating^13^. **(3) Tectonic transition**, occurs due to the buoyancy of the lower plate continental lithosphere counteracts the effects of slab-pull of the oceanic lithosphere resulting in their separation (slab breakoff^14^) (Fig. 1c). After slab breakoff, the collision zone transitions into an intracontinental setting. Slab breakoff will open a slab window and consequently trigger partial melting of differing magma source regions (by increasing the temperature of lithosphere and decreasing the pressure of asthenosphere), producing intense magmatism with compositional diversity (defined here as post-collisional magmatism) (including basaltic magmatism showing within-plate basalt geochemistry, bimodal magmatism, and anorogenic felsic magmatism, etc.)^15^,^16^. From initial to ongoing collision, the surface plate may not slow down significantly as the attached continental lithosphere is thin with less resistance to subduct due to the descent of the dense oceanic lithosphere (slab pull), whereas after slab breakoff during the tectonic transition the velocity of the surface plate is expected to decrease significantly due to the loss of the slab pull force that is the main driving force of plate motion^10^,^11^.

The evolving magmatic record from ongoing subduction to collision, slab breakoff, and further continental lithospheric interaction provides a framework to evaluate continent-continent collision. This is because slab breakoff will postdate the initial continent-continent collision by several to ten million years, depending on convergence velocity, subducting slab dip^8^, and shape of the colliding margins. Thus, defining the timing of slab breakoff, which can be identified on the integration of geological, geochemical, geochronological, and geophysical methods^15^,^16^,^17^,^18^,^19^,^20^, provides an important time-stamp on the sequence of collision-related events.

The Gangdese arc in southern Tibet (Fig. 2a), which records the subduction of the Neo-Tethyan oceanic lithosphere and subsequent India-Asia collision^21^, allows a direct test of our petrological approach to resolving the timing of India-Asia collision. This is because the voluminous Linzizong volcanic rocks (Fig. 2b,c) and coeval intrusive rocks of the Gangdese arc (Fig. 2d) range in age from 70 to 40 Ma^22^, straddling the interpreted timing of collision^1^,^2^,^3^,^4^,^5^,^6^,^7^,^8^,^9^. We provide the first comprehensive dataset on the age and geochemistry of these rocks enabling us to tightly constrain the progressive history of convergence and collision, including redefining the timing of the latter.

## Spatial, temporal and compositional changes of the Gangdese arc

The Linzizong volcanic rocks (Fig. 2b) extend for more than 1000 km along the southern Lhasa Terrane and are well exposed in Linzhou Basin (Fig. S1). In this basin, the lower unit of the Linzizong volcanic rocks (Dianzhong Formation) is dominated by thick andesitic rocks (Fig. 2c), which unconformably overlie strongly folded Upper Cretaceous siltstone and mudstone (Fig. S1). The middle unit (Nianbo Formation) is characterized by siltstone, marl, and limestone interbedded with andesitic rocks (Fig. 2c). The upper unit (Pa’na Formation) is characterized by the presence of thick rhyolite and rhyolitic ignimbrite (Fig. 2c) with columnar jointing (Fig. S1). We undertook SIMS (secondary ion mass spectrometry) U-Pb zircon dating^23^ of magmatic rocks from the stratigraphic boundaries of each formation (Fig. S1). Sample details, zircon U-Pb age data, and geochemical data are provided in Tables S1 and S2 in the supplementary material. Our new age data are shown in red ovals with numerals in Fig. 2c and S1 and are summarized in Table 1. The SIMS U-Pb zircon age data of two samples (13LZ01-1 and 13LZ17-1) from the lowermost Dianzhong Formation (60.2 ± 0.6 and 60.2 ± 0.8 Ma, respectively) and of one sample (13LZ08-1) from the uppermost Dianzhong Formation (58.3 ± 1.3 Ma) indicate that the Dianzhong andesitic volcanism was most likely active during 60.2–58.3 Ma. One sample (12LZ29-1) from the lowermost Nianbo Formation (55.4 ± 0.5 Ma) and two samples (12LZ27-1 and 13LZ16-1) from the Upper Nianbo Formation (52.6 ± 0.4 and 52.7 ± 1.9 Ma, respectively) provide age constraint on the Nianbo Formation of 55.4–52.6 Ma. This is very compatible with the age data obtained from two marl samples (13LZ13-1 and 13LZ14-1) from the lower Nianbo Formation (54.4 ± 0.5 and 54.5 ± 0.7 Ma). Three samples from the Lower (12LZ25-1), Middle (13LZ05-1), and Uppermost (13LZ04-1) Pa’na Formation give SIMS U-Pb zircon ages of 52.3 ± 0.5, 52.6 ± 0.4, and 52.3 ± 0.6 Ma, indistinguishable within analytical errors. These data precisely bracket the duration of each formation of the Linzizong volcanic rocks from the Linzhou Basin: 60.2–58.3 Ma for the Dianzhong Formation, 55.4–52.6 Ma for the Nianbo Formation, and 52.6–52.3 Ma for the Pa’na Formation. Thus the duration of magmatic activity is some 8 m.y. defined by SIMS zircon U-Pb dating and differs significantly from previous estimates of up to 25 m.y. based on laser ablation inductively coupled plasma mass spectrometry (LA-ICPMS) zircon U-Pb and Ar-Ar dating for the Dianzhong (69–61 Ma), Nianbo (61–54 Ma), and Pa’na (54–44 Ma) formations (see ref. 24 for age summary).

The Gangdese Batholith extends over 1500 km along the southern Lhasa Terrane (Fig. 2a). It is composed mainly of diorite and granodiorite, together with abundant mafic enclaves and dykes (Fig. S1). To obtain a comprehensive dataset with adequate spatial coverage, we collected a total of 127 samples with LA-ICPMS U-Pb zircon age data and 213 samples with whole-rock geochemical data on the 80–40 Ma intrusive rocks extending from longitude E85° to E95° along the strike of the batholith (Fig. 2d). Sample details, zircon U-Pb age data, and SiO_2_ contents are provided in Tables S3 and S4 in the supplementary material. These age data reveal that the >72 Ma magmatism was confined to a narrow belt in the south (blue dashed line, Fig. 2d), shifting northward at 71–65 Ma (red dashed line), then south at 64–48 Ma, which spread over a relatively broader area than the earlier activity, and is finally (47–38 Ma) largely restricted to its southern edge. These age data define a pulse of magmatic flare-up event at ca. 51 Ma along the entire length of the arc (Fig. 2e).

Changes in the chemical composition of the Linzizong volcanic rocks available from the Linzhou Basin and the Gangdese Batholith with time are illustrated by SiO_2_ variation and zircon saturation temperature^26^ against age (Fig. 3). Figure 3a shows that an andesite-dacite association in the Dianzhong Formation was followed by bimodal volcanic suites in both the Nianbo and Pa’na formations. Figure 3b reveals an increase in zircon saturation temperature at ca. 52 Ma documented by the rhyolitic rocks in the Pa’na Formation. Figure 3c illustrates felsic-dominated magmatism in the Gangdese Batholith during 80–73 Ma, followed by significant mafic magmatic activity at 70–43 Ma. It is important to note that bimodal magmatism coeval with or slightly younger than the Nianbo and Pa’na bimodal volcanism is most likely developed within the Gangdese Batholith as indicated by the presence of ca. 52–47 Ma gabbroic dykes that intruded into the coeval granitoid (Fig. S1g). The absence of compositional gap in the whole Gangdese Batholith (Fig. 3c) is probably the consequences of magma mixing between felsic and basaltic melts^27^ as indicated by the well-developed coeval mafic enclaves (Figs S1h, S1i, and S1j).

## Discussion

Temporal trends for enhanced mafic magmatism and increased zircon saturation temperature within the Linzizong volcanic rocks and Gangdese Batholith indicate an increased mantle heat input. It is emphasized that increased mantle input and coeval magmatic flare-up are difficult to attribute to a large scale change in the stress state of the lithosphere. This is because such change is a shallow response to deep mantle dynamics and is not an effective mechanism that will produce extensive magmatic activity. One possible explanation for higher mantle heat input at 70–43 Ma was the removal of the Asian lithosphere following tectonic shortening between 90 and 69 Ma^28^. However, to account for the southward migration of the magmatism from 72–65 Ma to 64–48 Ma (Fig. 2d), and significantly increased zircon saturation temperature at ca. 52 Ma and peak activity at ca. 51 Ma (Fig. 2e), we argue for slab steepening (Fig. 4a,b) and rollback (Fig. 4c) followed by slab breakoff (Fig. 4d).

We infer that the breakoff of the Neo-Tethyan lithosphere occurred slightly earlier (e.g., ca. 53 Ma; Fig. 4d) than the rapid eruption of ca. 2 km thick rhyolite and rhyolitic ignimbrite (52.5–52.3 Ma) (Fig. 2c) documented by the Linzizong volcanic rocks (Pa’na Formation) and the magmatic flare-up event of ca. 51 Ma shown by the age relationships within the Gangdese Batholith (Fig. 2e). This inference is consistent with numerical modeling that indicates a short duration of slab breakoff (<2 Ma^29^) followed by intense magmatism as a result of the enhanced heat input from rising asthenosphere^30^. Other robust lines of evidence supporting our model of slab breakoff by this time include: (1) the occurrence of abundant 52–47 Ma mafic enclaves and dykes (Fig. S1) that suggest significantly increased contributions from the mantle; (2) the presence of ca. 52.5 Ma bimodal volcanic rocks (Fig. 3a) that points to partial melting of enriched metasomatic layers within lithospheric mantle and to crustal melting caused by thermotectonic effects as a result of slab breakoff^15^; (3) high Zr/Y ratios (3.7–6.8) of the basaltic lavas and dykes (ca. 52.9 Ma) in the Linzizong volcanic rocks (Table S2) that suggest input of subslab asthenospheric mantle^16^; and (4) dramatically increased zircon saturation temperature at ca. 52.5 Ma recovered by the rhyolitic rocks of the Pa’na Formation (Fig. 3b) that indicates the anomalously high heat input from the mantle.

The development of magmatic activity at 51–43 Ma in the Gangdese arc (Fig. 3b), as well as ca. 50 Ma diabase dykes that intrude serpentinitized peridotite within the Yarlung-Zangbo suture zone (Fig. 2d), is most likely the consequences of partial melting of differing magma source regions through increasing temperature of lithosphere and decreasing pressure of asthenosphere after slab breakoff. This interpretation is consistent with numerical modeling which indicates that hot asthenosphere continues to ascend and generates melt for several million years after slab breakoff (Fig. 4e)^30^. The shift in the whole-rock Nd and zircon Hf isotopic compositions towards negative values at ca. 50 Ma^6^,^31^,^32^ most likely reflects a profound change of the source regions associated with the involvement of slab edge materials of the Indian continent that had already been subducted to depths^19^,^32^ (Fig. 4e), rather than indicative of the initial India-Asia collision^6^.

**Figure 1:**
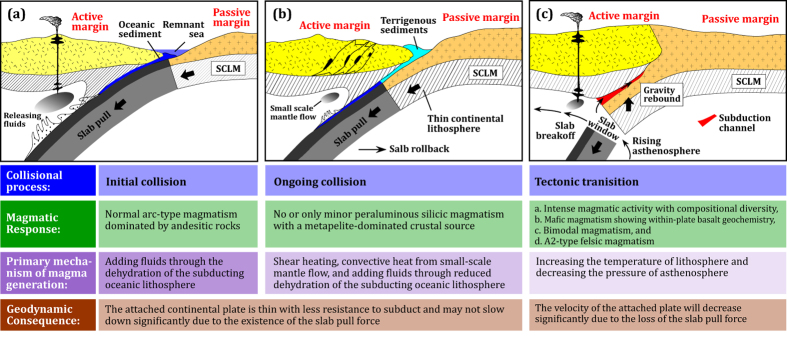
Schematic sequence of the relationship between collisional processes and magmatic responses in collision zones. This figure is generated by Di-Cheng Zhu, using Adobe Illustrator CS4 created by the Adobe Illustrator Team under an open license.

**Figure 2:**
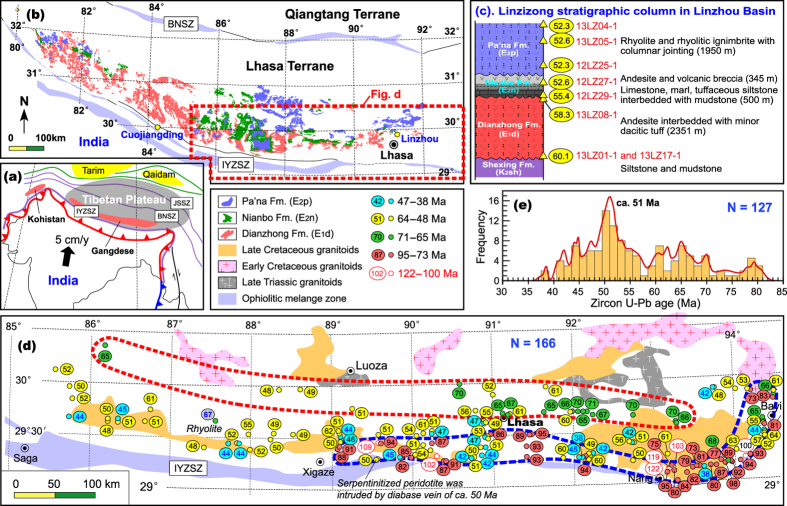
Tectonic framework of the Tibetan Plateau and the Lhasa Terrane. (**a**) Showing the Gangdese arc in the context of the Tibetan Plateau. IYZSZ = Indus-Yarlung Zangbo suture zone, BNSZ = Bangong-Nujiang suture zone, JSSZ = Jinsha suture zone. (**b**) The distribution of the Linzizong volcanic rocks. (**c**) The stratigraphic column of the Linzizong volcanic rocks in Linzhou Basin^4^. The filled ovals with numerals are host-rock crystallization ages in Ma using *in situ* zircon secondary ion mass spectrometry (SIMS) U-Pb dating method (see supplementary Table S1 for sample details). (**d**) The distribution of intrusive rocks in the Gangdese arc. The filled circles indicate sample locations, numerals within ovals are host-rock crystallization ages in Ma using *in situ* zircon LA-ICPMS U-Pb dating method (see supplementary Table S2 for sample details). Five groups of zircon ages are recognized on the basis of spatial variation of magmatism and different magmatic origin^22^. This figure is generated by Di-Cheng Zhu, using Adobe Illustrator CS4 created by the Adobe Illustrator Team under an open license. (**e**) Histogram of crystallization ages (Ma) of the intrusive rocks (85–94°E) from the Gangdese Batholith. The red line represents frequency curve. Age data used in this histogram are the crystallization ages defined by the youngest group of zircon analyses of each sample. The bin width was set at 1.5 Ma to accommodate average age uncertainties of 1.1 Ma (2σ; Table S3). Only one age datum is selected for each pluton if between-sample age difference is lower than 3 Myrs. If this difference is more than 3 Myrs, this pluton is considered to emplace at different pulses and thus the different emplacement ages are used to construct the histogram.

The timing of rapid eruption of thick rhyolitic ignimbrite (ca. 52.5 Ma) in the Linzizong volcanic rocks and intense magmatism (ca. 51 Ma) in the Gangdese Batholith matches the sudden drop of the convergence rate of the Indian plate at ca. 51 Ma^33^,^34^,^35^. This synchronicity of events suggests that the slowdown of the Indian plate is largely the consequence of slab breakoff (Fig. 4e). This is because slab breakoff will result in the loss of the slab pull force^14^,^29^, which exerts a dominant influence on the velocity of the surface plate and cause a drastic change in plate motion^10^,^11^,^36^,^37^. Subsequent intracontinental convergence (Fig. 4f) of the Indian continental lithosphere beneath the Asian lithosphere after slab breakoff is likely driven by the subduction of dense Indian continental lithosphere^38^ and slab-pull of oceanic lithosphere on the Indo-Australian plate along the Indonesian segment of the plate margin^39^. Traditionally, the slow-down of the Indian plate at ca. 51 Ma is attributed to the increased resistance to subduction interpreted as a result of the initial India-Asia collision^33^,^34^. However, we consider this interpretation questionable because (1) under such a collisional regime, no appropriate mechanisms (see Fig. 1b) are available for producing extensive magmatism in southern Tibet as presented in this study (Fig. 2c and S1) and (2) the surface motion of the plate is closely related to or substantially driven by mantle dynamics (i.e., slab pull^10^,^11^,^36^,^37^) and thus if such a driving force disappears, significant slow down would occur.

**Figure 3:**
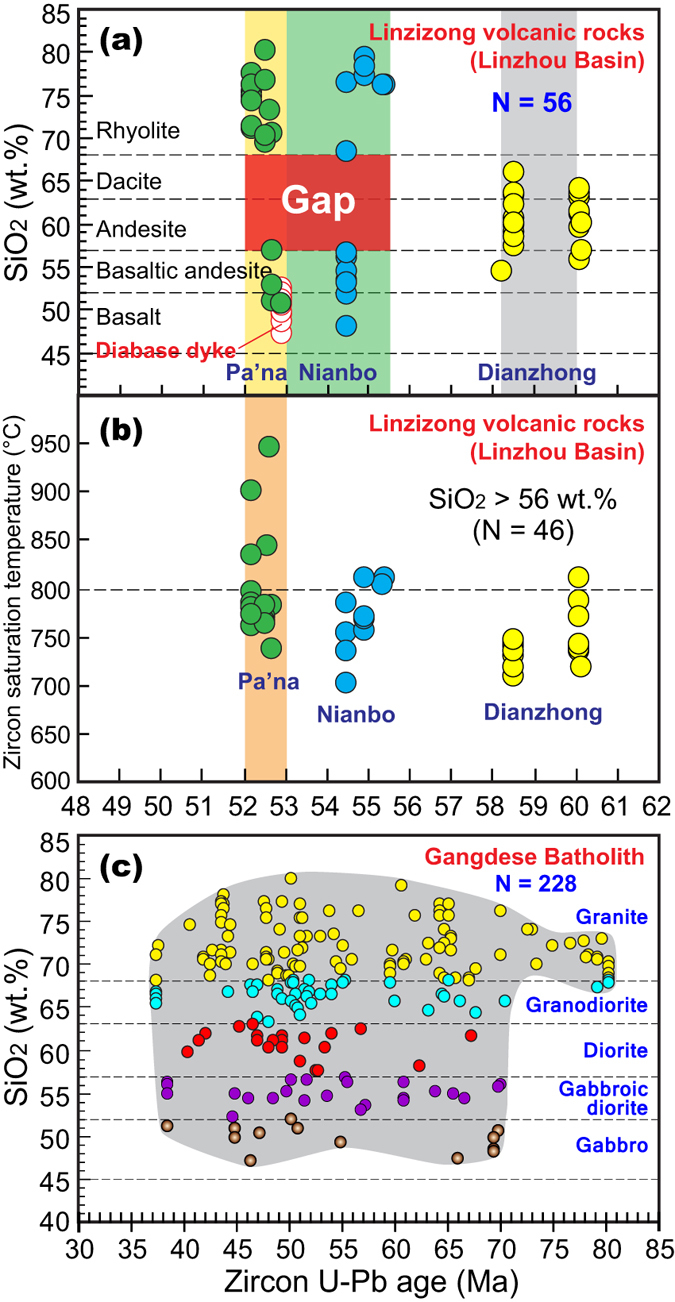
Changes in magmatic compositions with time in the Gangdese arc. (**a**,**b**) Plots of SiO_2_ content versus age (Ma) and of zircon saturation temperature (°C) versus age (Ma) for the Linzizong volcanic rocks (see supplementary Table S4 for geochemical data). Zircon saturation temperatures were calculated from whole-rock compositions with SiO_2_ >56 wt.% following the method of Watson and Harrison (1983)^26^. (**c**) Plot of SiO_2_ content versus age (Ma) for the Gangdese Batholith (E85°–E95°) (see Table S5 for geochemical data). Note that this plot did not show a clear increase of mafic magmatism at ca. 51 Ma as indicated by the presence of well-developed mafic enclaves and dykes (Fig. S1); this inconsistency reflects sampling bias with mafic material underrepresented.

It is beyond the scope of this article to discuss in detail the problems of each interpreted age (70–34 Ma) proposed for the initiation of the India-Asia collision (see ref. 9, for review). We emphasize that any estimates on the collisional timing must effectively explain all the first-order observations of the spatial, temporal (Fig. 2d), and compositional changes (Fig. 3) of magmatic activities summarized in this study. In particular, we must explain the specific geodynamical processes responsible for the southward migration of magmatism from 72–65 Ma to 64–48 Ma followed by the generation of dramatically enhanced magmatism precisely constrained at ca. 52 Ma in the Gangdese arc, including volumetrically significant ignimbrite in the Linzizong volcanic rocks (Fig. 2c) and widely developed mafic enclaves and dykes (Fig. S1) in the Gangdese Batholith. Such enhanced magmatism requires anomalously high-temperature material and heat supply from the mantle, which are unlikely to be explained by later slab breakoff (at ca. 50–40 Ma^40^, 45 Ma^18^, 48–44 Ma^41^, and 50 Ma^42^,^43^) that would predate intense magmatism^29^,^30^, or by ca. 50 Ma India-Asia or Tethyan Himalaya-Asia collision^44^ that would result in a compression regime without intense magmatism (Fig. 1b).

Eclogite-facies peak metamorphism in the western Himalaya was recently refined at ca. 47–43 Ma and consequently an age of ca. 51–47 Ma was proposed for the initial India-Asia collision^8^. However, this collision age is likely an underestimate because the exhumed ultrahigh-pressure rocks may not represent the materials from the leading edge of subducted Indian continental margin.

Slab breakoff is likely inevitable in all collision zones involving a passive continental margin^14^. It follows that subducted continental crust has reached its maximum depth when slab breakoff occurs. This means that slab breakoff provides a maximal age for continental collision. In the case of the India-Asia collision, if Neo-Tethyan slab breaks off at ca. 53 Ma, the initial India-Asia collision should commence at ca. 55 Ma. This is because the progressively enlarged subducting slab dip revealed by the southward migration of magmatism from 72–65 Ma to 64–48 Ma (Fig. 2d) and high India-Asia convergence velocity^35^ point to a time lag of ca. 1–2 Ma (Table S6) between initial collision and slab breakoff.

**Figure 4:**
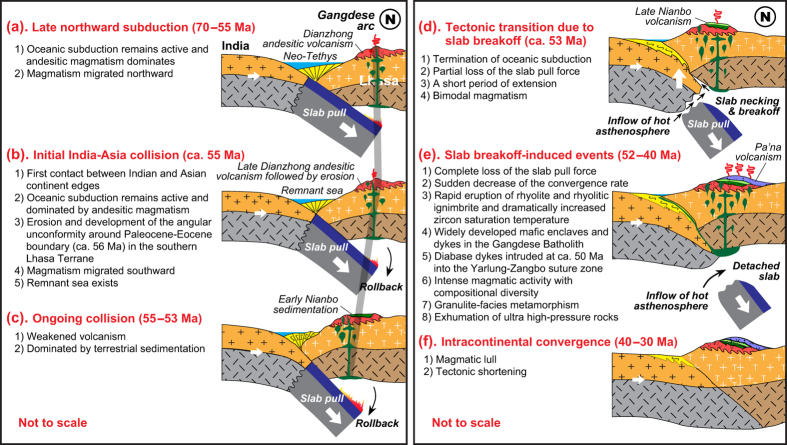
Schematic illustrations showing the India-Asia collisional processes and resultant tectonomagmatic activity over the past 70–40 m.y. (not to scale). This figure is generated by Di-Cheng Zhu, using Adobe Illustrator CS4 created by the Adobe Illustrator Team under an open license.

This timing of the initial India-Asia collision (ca. 55 Ma) obtained from our petrological approach is in good agreement with recent estimates on collision ages from the cessation of Xigaze forearc sedimentation (ca. 58–54 Ma^45^), the dramatic change of sedimentary environment and the development of an unconformity in the Xigaze forearc basin (>56 Ma^46^), the onset of India-Asia terrestrial faunal exchange (ca. 54 Ma^47^), the reappraisal of existing paleomagnetic data (52.4 ± 4.5 Ma^9^), and the new paleomagnetic data (ca. 54.3 Ma^48^). This collision may account for the angular unconformity between the Dianzhong and Nianbo formations in Linzhou Basin (Fig. 2c) and coeval unconformity found in the Xigaze forearc basin^46^ due to the locking of the subduction zone on arrival of the Indian continent at the trench^12^. Therefore, it seems most probable that (1) the Dianzhong andesitic volcanism was generated during the transition from late subduction to initial collision (Fig. 4a,b), (2) the lower Nianbo terrestrial sedimentation represents the ongoing India-Asia collision prior to slab breakoff with a short duration from initial to ongoing collision due to the high India-Asia convergence velocity^35^, and (3) the late Nianbo and Pa’na bimodal volcanic rocks (Fig. 3a) are linked to mantle decompression melting and crustal anatexis due to the ascent of hot asthenosphere through slab window after slab breakoff^30^ (Fig. 4d,e). Nevertheless, we note that the exact timing of initial impingement of the Indian margin with the subduction zone will depend on the degree of distention of the Asian margin and may vary along the strike of the convergence zone due to irregularities in shape of the margin.

Our work shows that the petrological approach that we employ here can effectively distinguish processes at varying stages of continental collision. This approach may be applied to other continent-continent collision zones also involving a passive continental margin on the down-going plate, such as the Arabia-Eurasia collision zone — where the preserved magmatic record straddles the proposed Arabia-Eurasia collision age^49^,^50^.

## Additional Information

**How to cite this article**: Zhu, D.-C. *et al.* Magmatic record of India-Asia collision. *Sci. Rep.* **5**, 14289; doi: 10.1038/srep14289 (2015).

## Supplementary Material

Supplementary Information

## Figures and Tables

**Table 1:** Summary of new zircon U-Pb age data reported in this study for the Linzizong volcanic rocks in Linzhou Basin, southern Tibet.

Formation	Sample	Rock Type	GPS position	Strata position	Datingmethod	Analyses	Age (Ma)	MSWD
Pa’na Formation	13LZ04-1	Rhyolitic breccia	N30°00.643′, E91°08.810′	Uppermost Pa’na	SIMS 1280	19	52.29 ± 0.61	2.5
	13LZ05-1^a^	Rhyolitic ignimbrite	N30°00.137′, E91°08.882′	Middle Pa’na	SIMS 1280	16	52.58 ± 0.40	1.3
	13LZ06-1	Rhyolite	N29°59.964′, E91°08.700′	Middle Pa’na	LA-ICPMS	16	50.5 ± 0.4	0.1
	12LZ23-1	Rhyolitic ignimbrite	N29°59.622′, E91°08.415′	Middle Pa’na	LA-ICPMS	16	49.7 ± 0.4	0.2
	12LZ25-1	Rhyolite	N29°59.313′, E91°08.474′	Lower Pa’na	SIMS 1280	13	52.27 ± 0.45	0.8
Nianbo Formation	13LZ16-1	Andesite	N29°59.078′, E91°11.209′	Upper Nianbo	SIMS 1280	4	52.7 ± 1.9	2.0
	12LZ27-1	Rhyolitic tuff	N29°58.557′, E91°08.736′	Upper Nianbo	SIMS 1280	15	52.64 ± 0.42	0.5
	13LZ13-1	Marl	N29°58.812′, E91°11.159′	Lower Nianbo	SIMS 1280	14	54.35 ± 0.47	1.1
	13LZ14-1	Marl	N29°58.815′, E91°11.158′	Lower Nianbo	SIMS 1280	10	54.45 ± 0.68	1.3
	12LZ29-1	Rhyolite	N29°58.231′, E91°08.955′	Lowermost Nianbo	SIMS 1280	14	55.37 ± 0.45	0.4
Dianzhong Formation	13LZ08-1	Andesite	N29°58.708′, E91°11.195′	Uppermost Dianzhong	SIMS 1280	2	58.3 ± 1.3	0.01
	12LZ06-1	Dacite	N29°57.273′, E91°12.107′	Lower Dianzhong	LA-ICPMS	17	58.5 ± 0.5	0.4
	13LZ17-1^b^	Volcanic breccia	N29°57.292′, E91°13.048′	Lowermost Dianzhong	SIMS 1280	13	60.23 ± 0.78	1.8
	13LZ01-1^c^	Andesite	N29°57.117′, E91°11.855′	Lowermost Dianzhong	SIMS 1280	21	60.22 ± 0.61	2.0

^a^Rhyolitic ignimbrite with well-developed columnar jointing.

^b^ca. 50 cm above the angular unconformity between the Dianzhong and Shexing formations located ca. 78 m east of sample SH530022 that was dated by LA ICP-MS method at 68.7 ± 2.4 Ma (MSWD = 3.6)^25^.

^c^ca. 20 cm above the angular unconformity between the Dianzhong and Shexing formations.
