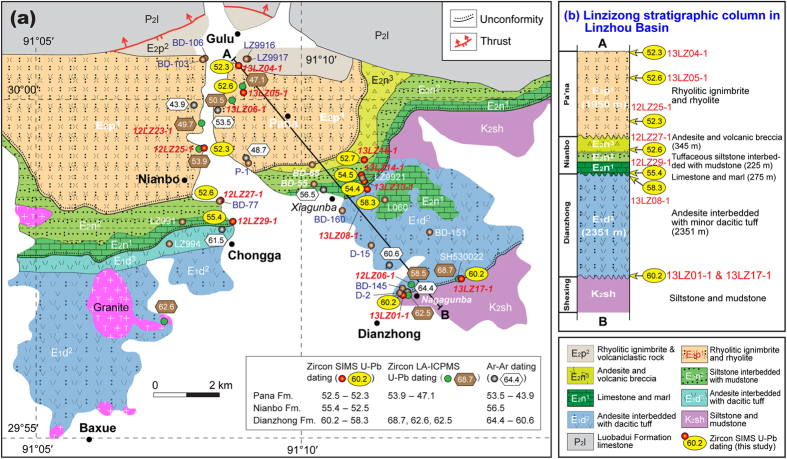# Corrigendum: Magmatic record of India-Asia collision

**DOI:** 10.1038/srep17236

**Published:** 2015-12-18

**Authors:** Di-Cheng Zhu, Qing Wang, Zhi-Dan Zhao, Sun-Lin Chung, Peter A. Cawood, Yaoling Niu, Sheng-Ao Liu, Fu-Yuan Wu, Xuan-Xue Mo

Scientific Reports 5 Article number: 14289; 10.1038/srep14289 published online: 09232015; updated: 12182015.

In Supplementary Figure 1a, the Linzizong volcanic rock samples ‘12LZ14-1’ and ‘12LZ13-1’ should read ‘13LZ14-1’ and ‘13LZ13-1’ respectively. The correct Supplementary Figure 1a appears below as Fig. 1.

In Table S1, samples ‘12LZ13-1@02’ and ‘12LZ14-1@02’ should read ‘13LZ13-1@02’ and ‘13LZ14-1@02’ respectively.

## Figures and Tables

**Figure 1:**