# Supplementary material for: Magmatic record of India-Asia collision
Source: Sci Rep. 2015 Sep 23;5:14289. doi: 10.1038/srep14289 (PMC4585790; doi:10.1038/srep14289)
Supplement: Supplementary Information [file srep14289-s1.pdf]

Online supplementary information

Magmatic record of India-Asia collision

Di-Cheng Zhu*, Qing Wang, Zhi-Dan Zhao, Sun-Lin Chung, Peter A. Cawood, Yaoling Niu, Sheng-Ao Liu, Fu-Yuan Wu, and Xuan-Xue Mo

Corresponding author, Email: dchengzhu@163.com

Includes the following materials:

1. Figure S1 Distribution and field occurrences the Gangdese arc rocks in southern Tibet

2. Zircon U-Pb dating methods, data, and geochemical data

SIMS U-Pb dating

LA-ICPMS U-Pb dating

Concordia plots for single zircon analyzed by SIMS 1280 U–Pb dating

Concordia plots for single zircon analyzed by LA-ICPMS U–Pb dating

Table S1 Zircon SIMS U-Pb age data reported in this study for the Linzizong volcanic rocks in Linzhou Basin

Table S2 Zircon LA-ICPMS U-Pb age data reported in this study for the Linzizong volcanic rocks in Linzhou Basin

Table S3 Zircon U-Pb age data of the 80–40 Ma intrusive rocks from the Gangdese Batholith (85–95°E) in southern Tibet

Table S4 Geochemical data of the Linzizong volcanic rocks in Linzhou Basin

Table S5 SiO_2 contents of the 80–40 Ma intrusive rocks from the Gangdese Batholith (85–95°E) in southern Tibet

Table S6 Calculation for the timing of collision

3. References Cited in Tables S3, S4, and S5

Magmatic record of India-Asia collision

Di-Cheng Zhu*, Qing Wang, Zhi-Dan Zhao, Sun-Lin Chung, Peter A. Cawood, Yaoling Niu, Sheng-Ao Liu, Fu-Yuan Wu, and Xuan-Xue Mo

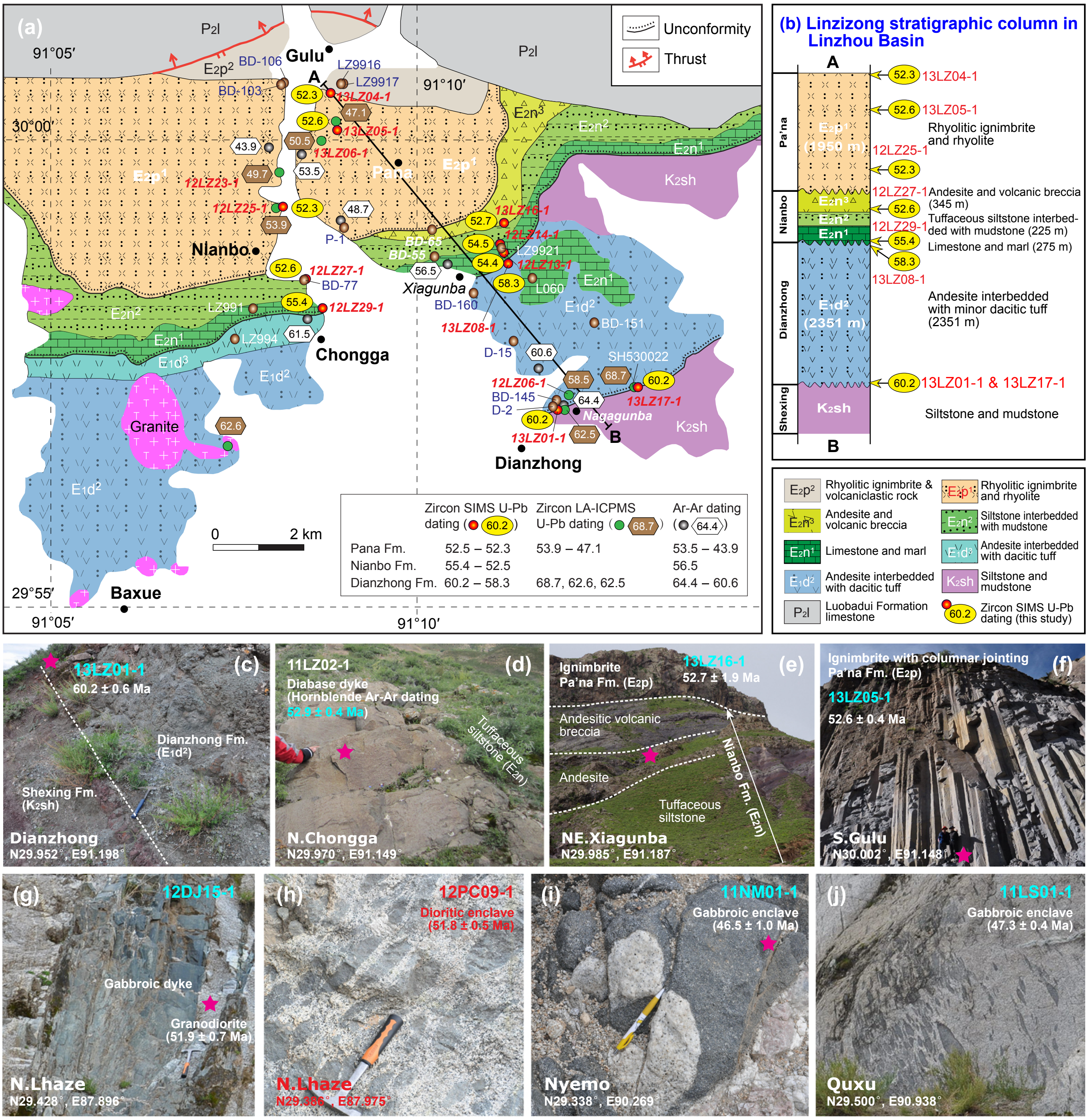

Figure S1 Distributions and field occurrences of the Gangdese arc rocks in southern Tibet
a - Distributions of the Linzizong volcanic rocks in Linzhou Basin (modified from Dong et al., 2005);
b - The stratigraphic column of the Linzizong volcanic rocks in Linzhou Basin (modified from Mo et al., 2007);
c, d, e, and f - Photos of the Linzizong volcanic rocks in Linzhou Basin taken by Di-Cheng Zhu (2011, 2013);
g, h, i, and j - Photos of the granitoids in the Gangdese Batholith taken by Di-Cheng Zhu (2011, 2012). This figure is generated by Di-Cheng Zhu, using Adobe Illustrator CS4 created by the Adobe Illustrator Team under an open license.

References:

Dong, G., Mo, X., Zhao, Z., Wang, L., Zhou, S. A new understanding of the stratigraphic successions of the Linzizong volcanics in the Linzhou Basin, northern Lhasa, Tibet, China. Geol. Bull. China 24, 549–557 (in Chinese with English abstract) (2005).
Mo, X.X. et al. Mantle contributions to crustal thickening in south Tibet in response to the India-Asia collision. Lithos 96, 225–242 (2007).

2. Zircon U-Pb dating methods, data, and Geochemical data

SIMS U-Pb dating:

Samples for U-Pb analysis were processed by conventional magnetic and density techniques to concentrate non-magnetic, heavy fractions. Zircon grains, together with zircon standard 91500 were mounted in epoxy mounts which were then polished to section the crystals in half for analysis. All zircons were documented with transmitted and reflected light micrographs as well as cathodoluminescence (CL) images to reveal their internal structures, and the mount was vacuum-coated with high-purity gold prior to secondary ion mass spectrometry (SIMS) analysis.

Measurements of U, Th and Pb were conducted using the Cameca IMS-1280 SIMS at the Institute of Geology and Geophysics, Chinese Academy of Sciences in Beijing. U-Th-Pb ratios and absolute abundances were determined relative to the standard zircon 91500 (Wiedenbeck et al., 1995), analyses of which were interspersed with those of unknown grains, using operating and data processing procedures similar to those described by Li et al. (2009). A long-term uncertainty of 1.5% (1 RSD) for $^{206}Pb/^{238}U$ measurements of the standard zircons was propagated to the unknowns (Li et al., 2010), despite that the measured $^{206}Pb/^{238}U$ error in a specific session is generally around 1% (1 RSD) or less. Measured isotopic compositions were corrected using the ^{207}Pb-based correction method and were shown as Tera-Wasserburg plots (Li et al., 2010). Uncertainties on individual analyses in data tables are reported at a 1 level; mean ages for pooled U/Pb (and Pb/Pb) analyses are quoted with 95% confidence interval. Data reduction was carried out using the Isoplot/Ex v. 2.49 program (Ludwig, 2001). The lower intercept age in the Tera-Wasserburg plot of the Qinghu standard measured during the sample runs is 160.22 ± 0.86 Ma (46 analyses, 2σ; MSWD = 1.4), identical to the result (159.5 ± 0.7 Ma) reported by Li et al. (2009).

References:

Li, Q.L., Li, X.H., Liu, Y., Tang, G.Q., Yang, J.H., Zhu, W.G., 2010. Precise U-Pb and Pb-Pb dating of Phanerozoic baddeleyite by SIMS with oxygen flooding technique. Journal of Analytical Atomic Spectrometry 25, 1107−1113.

Li, X.H., Liu, Y., Li, Q.L., Guo, C.H., Chamberlain, K.R., 2009. Precise determination of Phanerozoic zircon Pb/Pb ageby multicollector SIMS without external standardization, Geochemistry, Geophysics, Geosystems 10, Q04010, doi: 10.1029/2009GC002400.

Ludwig, K.R., 2001. Users manual for Isoplot/Ex rev. 2.49. Berkeley Geochronology Centre Special Publication. No. 1a, 1–56 pp.

Stacey, J.S., Kramers, J.D., 1975. Approximation of terrestrial lead isotope evolution by a two-stage model. Earth and Planetary Science Letters 26, 207–221.

Wiedenbeck, M., Alle, P., Corfu, F., Griffin, W.L., Meier, M., Oberli, F., Vonquadt, A., Roddick, J.C., Speigel, W., 1995. Three natural zircon standards for U-Th-Pb, Lu-Hf, trace-element and REE analyses. Geostandards Newsletter 19, 1–23.

LA-ICPMS U-Pb dating:

The Zircon U-Pb isotopic analyses were performed using the LA-ICPMS housed at the State Key Laboratory of Geological Processes and Mineral Resources, China University of Geosciences (Wuhan). The detailed operating conditions for the laser ablation system, the ICP-MS instrument, and data reduction followed those described in Liu et al. (2010). ICPMSDataCal (Liu et al., 2010) was used for off-line selection and the integration of background and analyse signals, time-drift correction, U–Pb dating, and quantitative calibration for trace element analyses. The common Pb correction followed the ComPbCorr#3-151 procedure (Andersen, 2002). Age calculations and plotting of concordia diagrams were made using ISOPLOT (ver 3.0) (Ludwig, 2003). Uncertainties of individual analyses are reported as 1σ; mean ages for pooled $^{206}Pb/^{238}U$ results are reported as 2σ.

References:

Andersen, T., 2002. Correction of common lead in U–Pb analyses that do not report ^{204}Pb. Chemical Geology 192, 59–79.

Liu, Y.S., Gao, S., Hu, Z.C., Gao, C.G., Zong, K.Q., Wang, D.B., 2010. Continental and Oceanic Crust Recycling-induced Melt-Peridotite Interactions in the Trans-North China Orogen: U–Pb Dating, Hf Isotopes and Trace Elements in Zircons from Mantle Xenoliths. Journal of Petrology 51, 537–571.

Ludwig, K.R., 2003. Isoplot 3.00: a geochronological toolkit for Microsoft Excel. Berkeley Geochronology Center, Special Publication No. 4, pp. 1–70.

Magmatic record of India-Asia collision

Di-Cheng Zhu*, Qing Wang, Zhi-Dan Zhao, Sun-Lin Chung, Peter A. Cawood, Yaoling Niu, Sheng-Ao Liu, Fu-Yuan Wu, and Xuan-Xue Mo

Concordia plots for single zircon analyzed by SIMS 1280 U–Pb dating

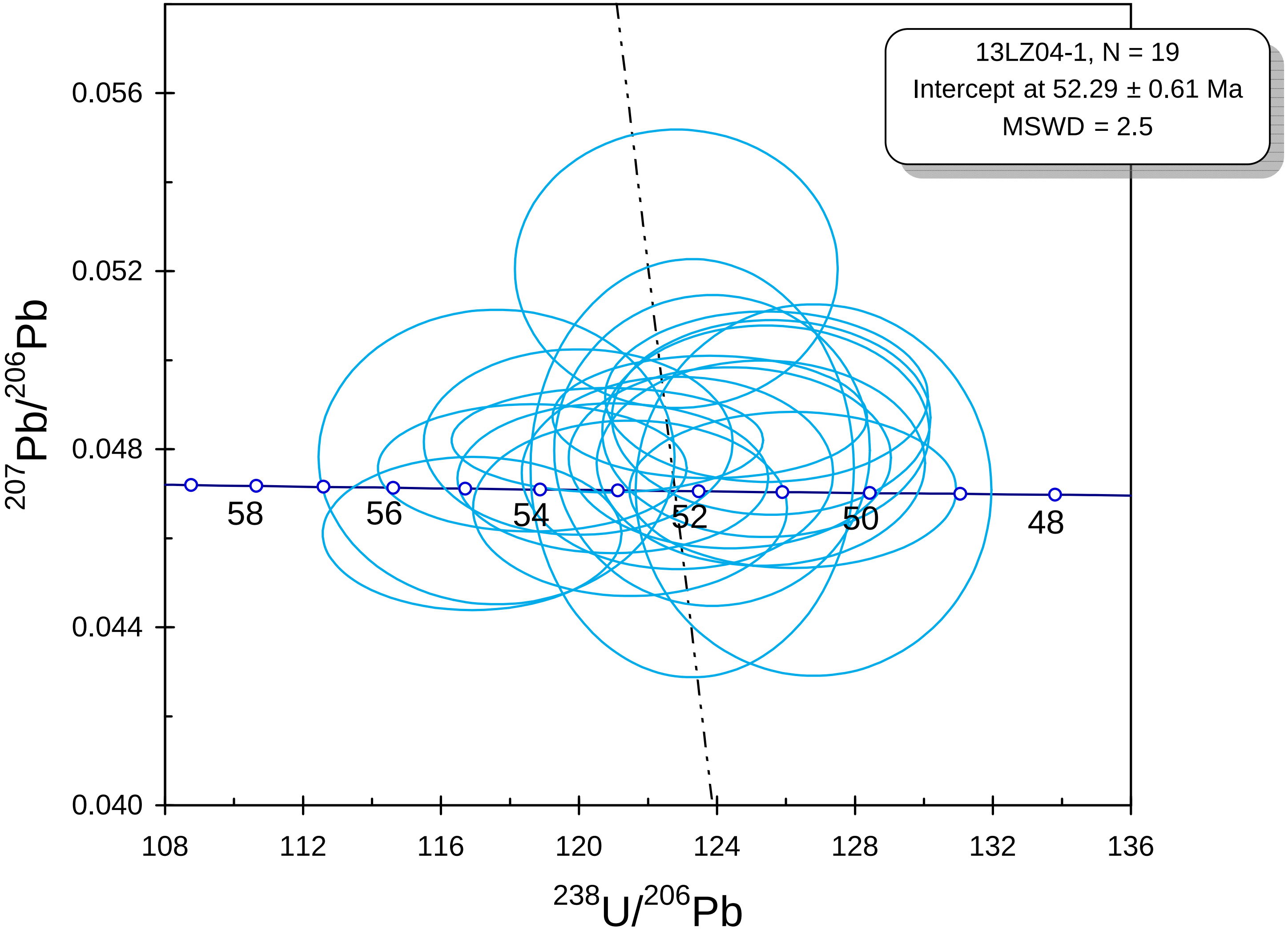

Concordia plots for single zircon analyzed by SIMS 1280 U–Pb dating

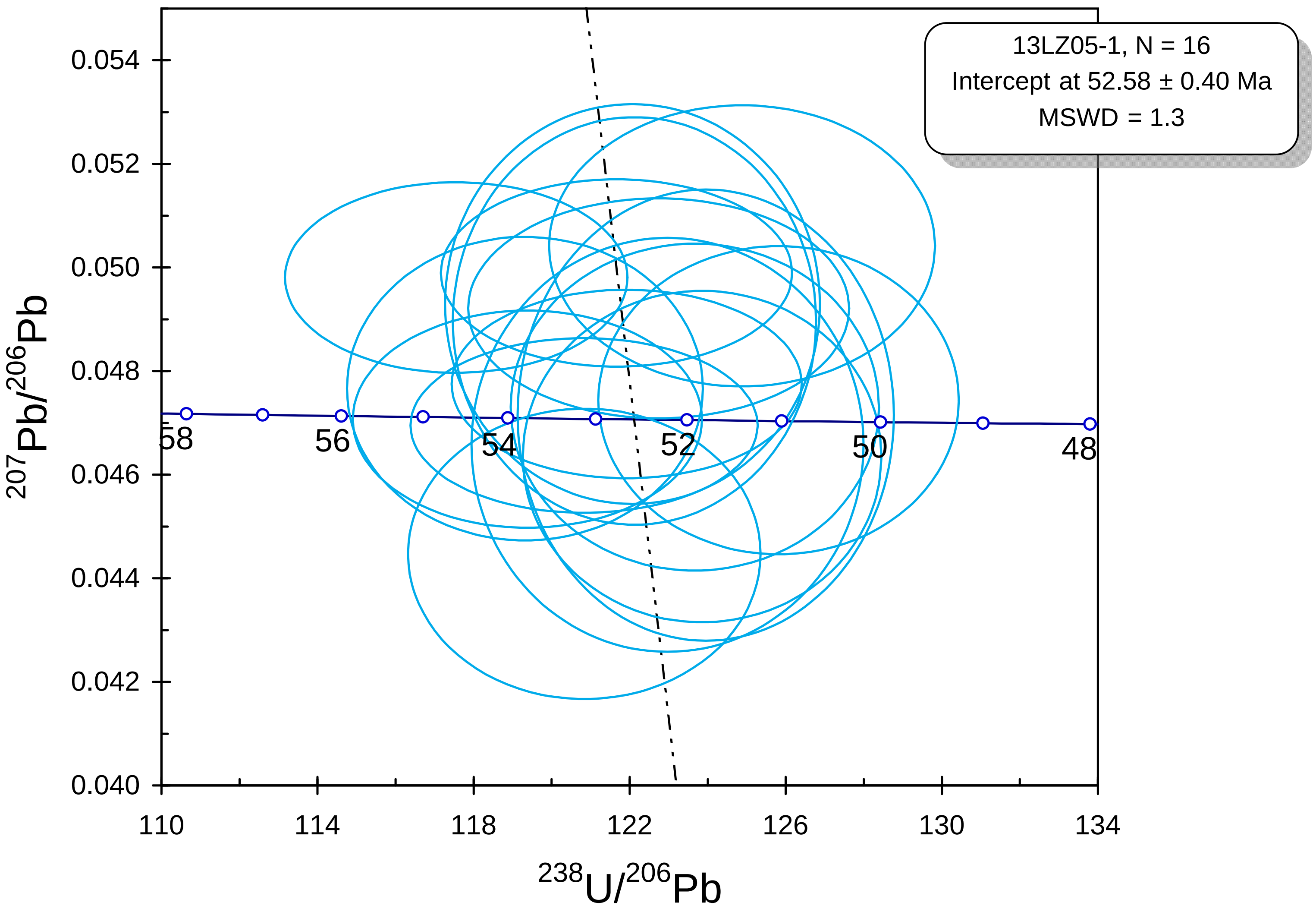

Concordia plots for single zircon analyzed by LA-ICPMS U–Pb dating

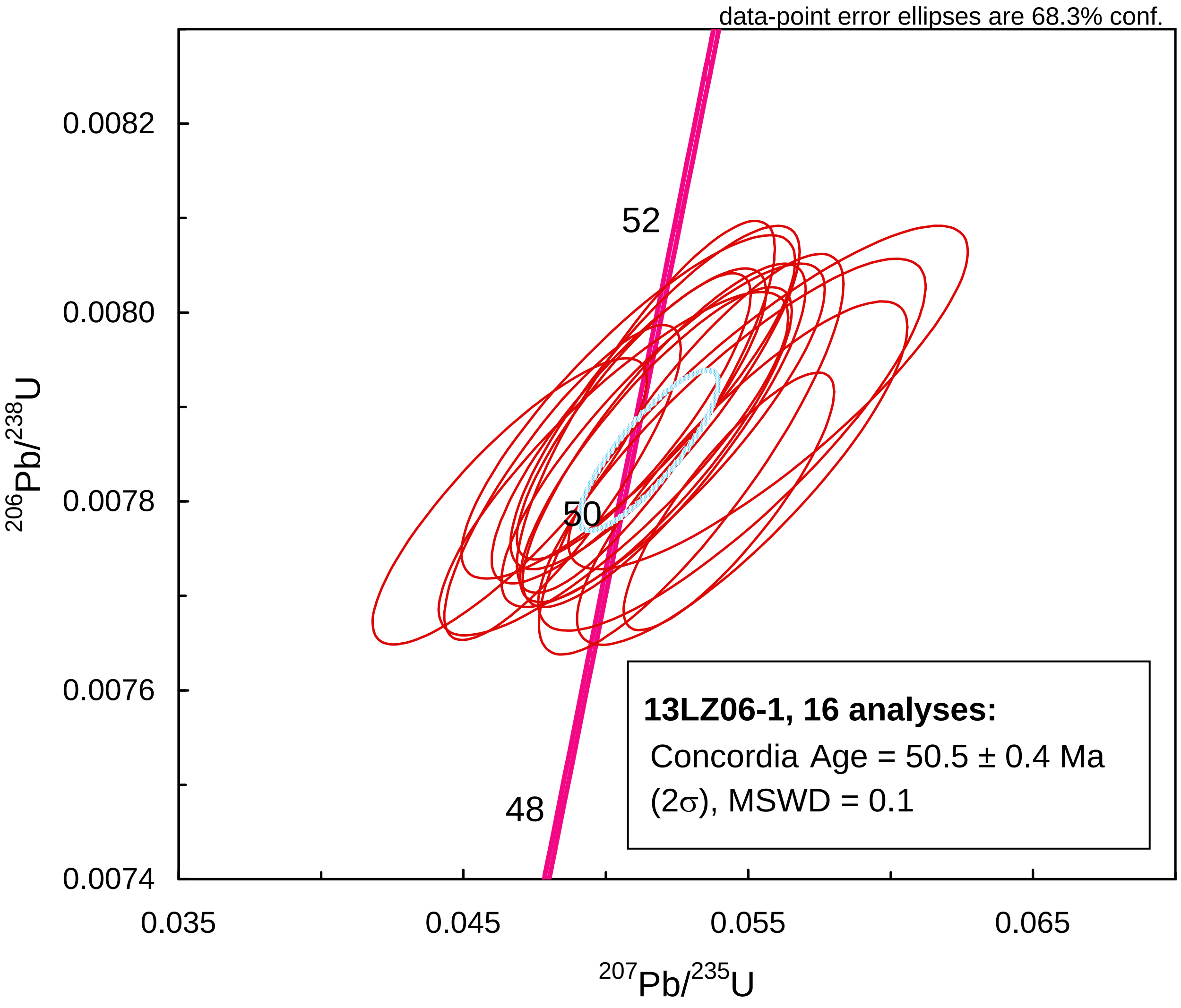

Concordia plots for single zircon analyzed by LA-ICPMS U–Pb dating

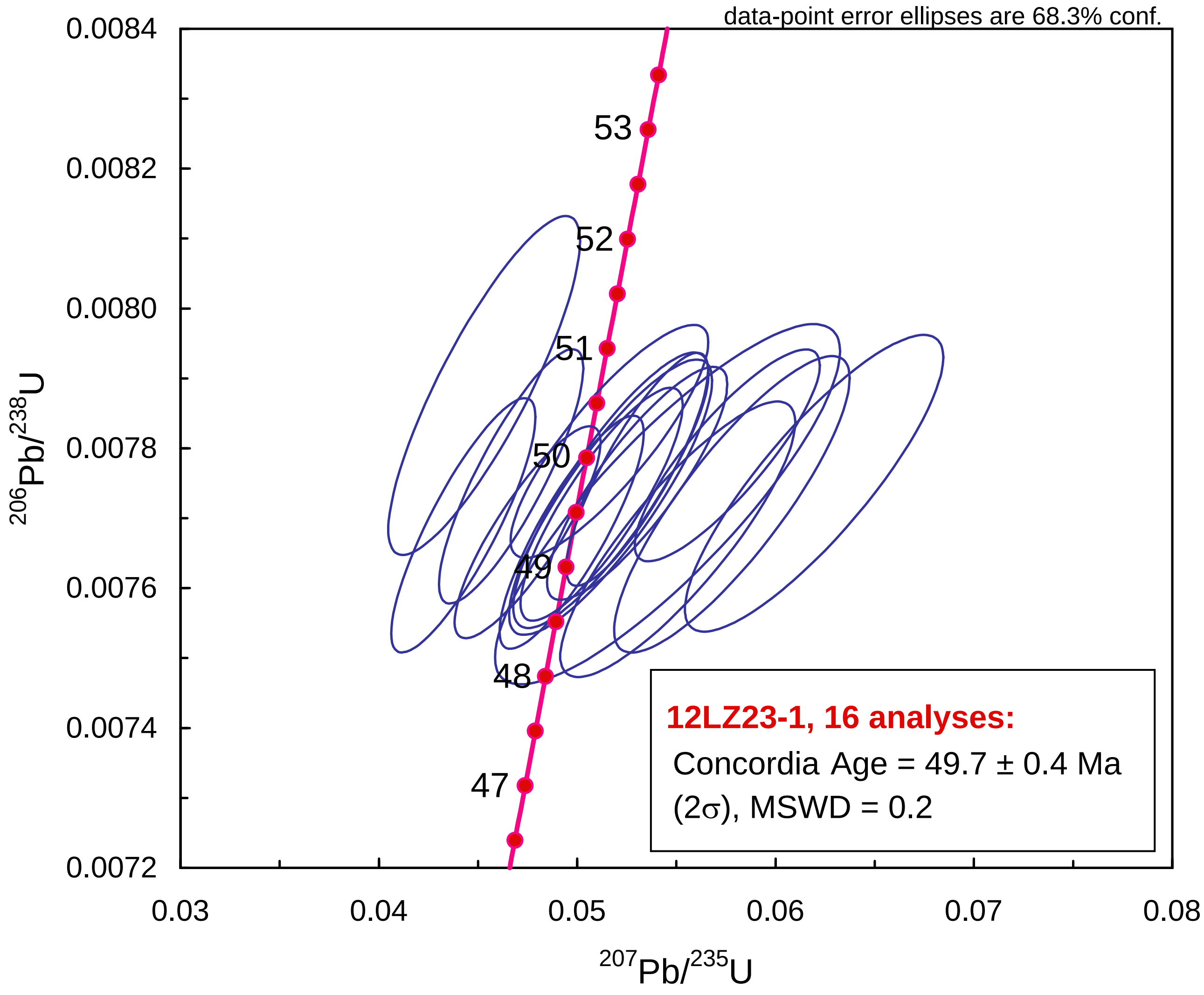

Concordia plots for single zircon analyzed by SIMS 1280 U–Pb dating

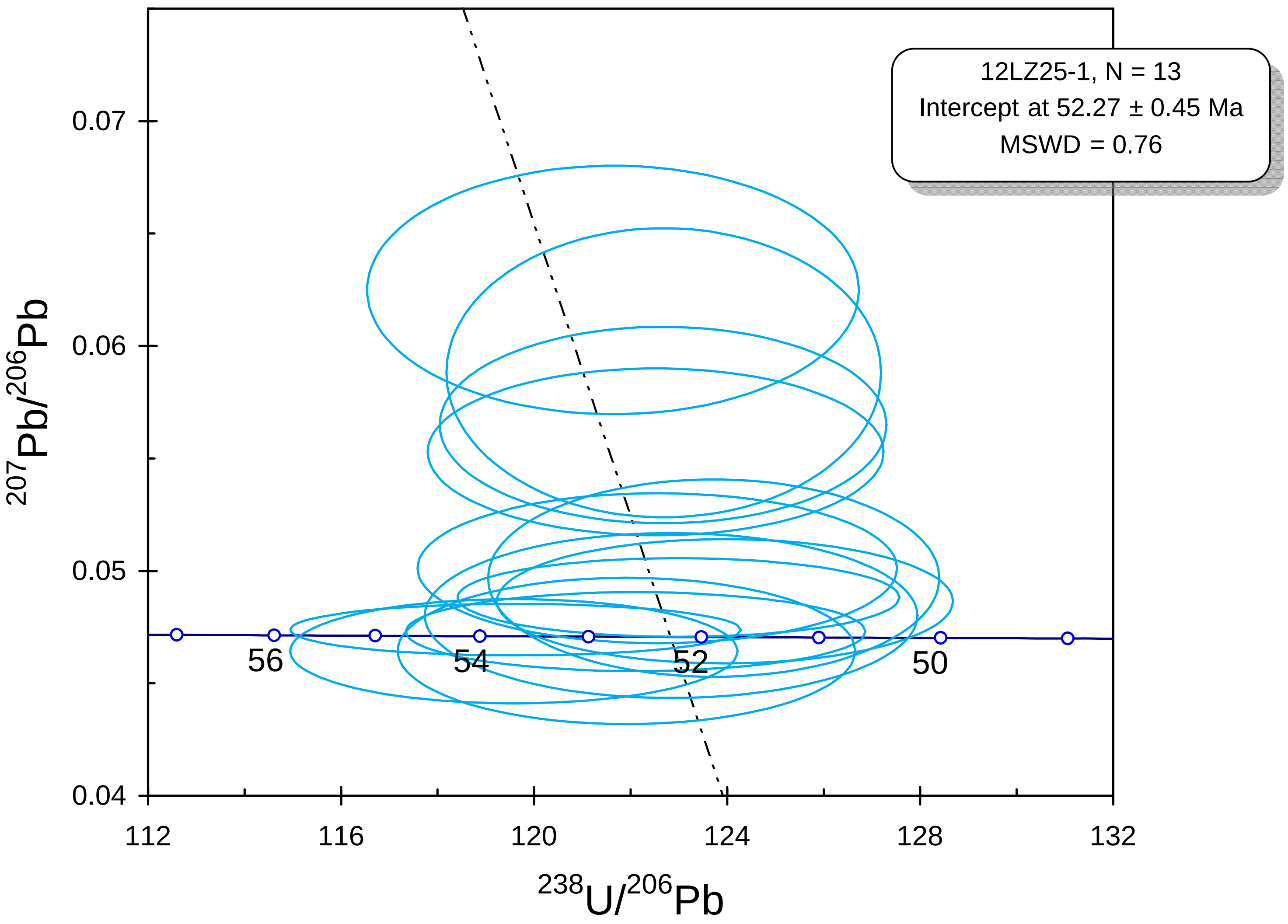

Concordia plots for single zircon analyzed by SIMS 1280 U–Pb dating

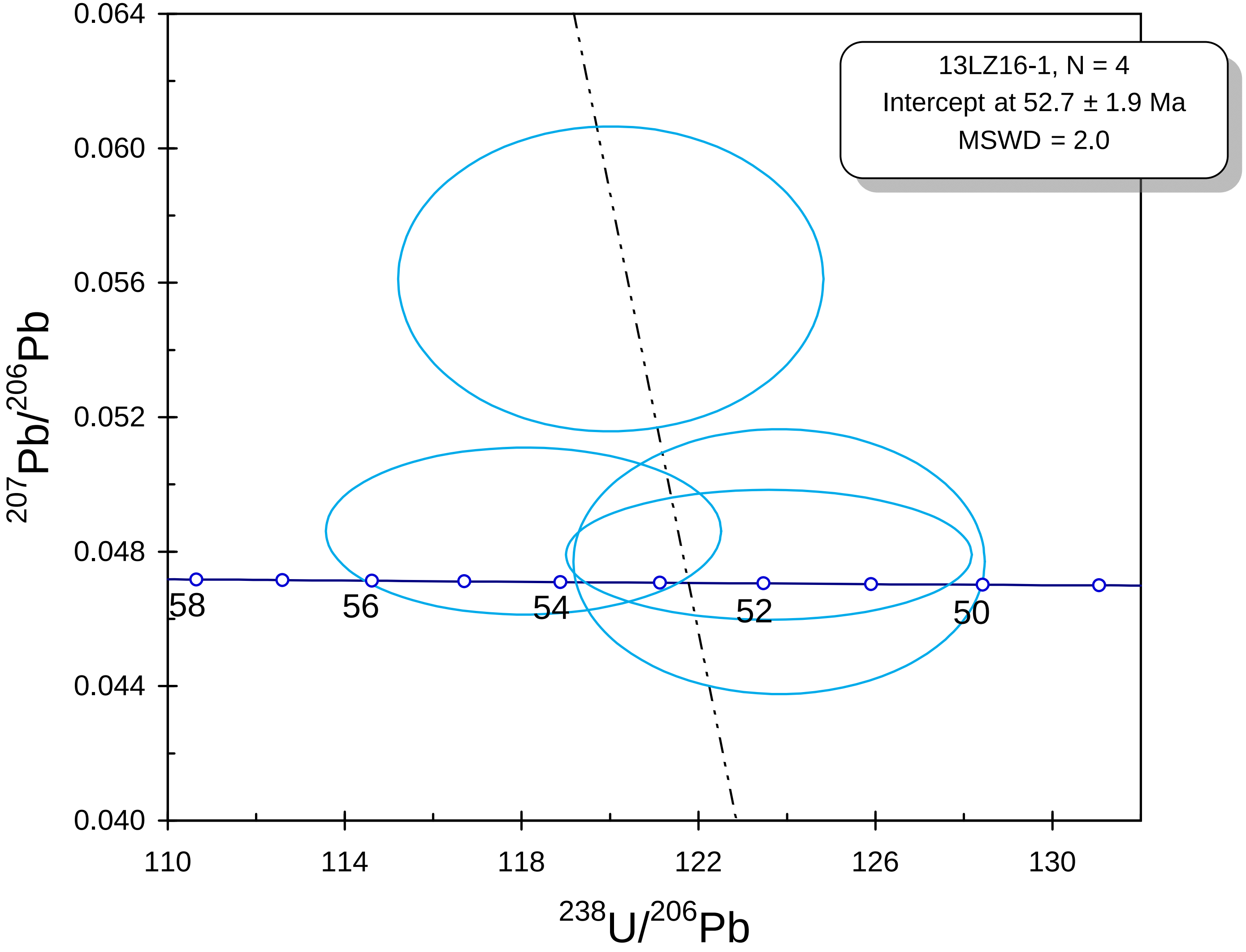

Concordia plots for single zircon analyzed by SIMS 1280 U–Pb dating

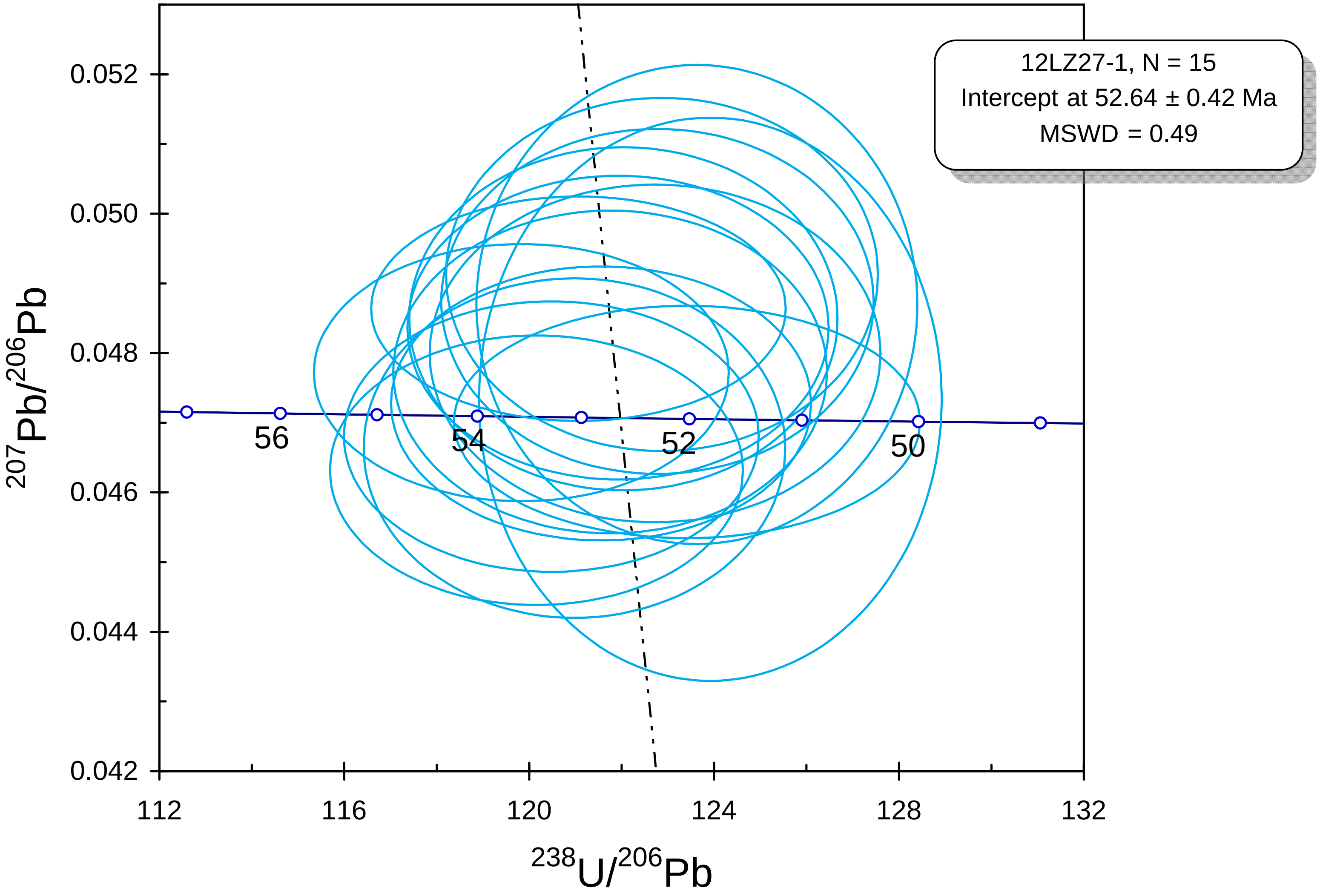

Concordia plots for single zircon analyzed by SIMS 1280 U–Pb dating

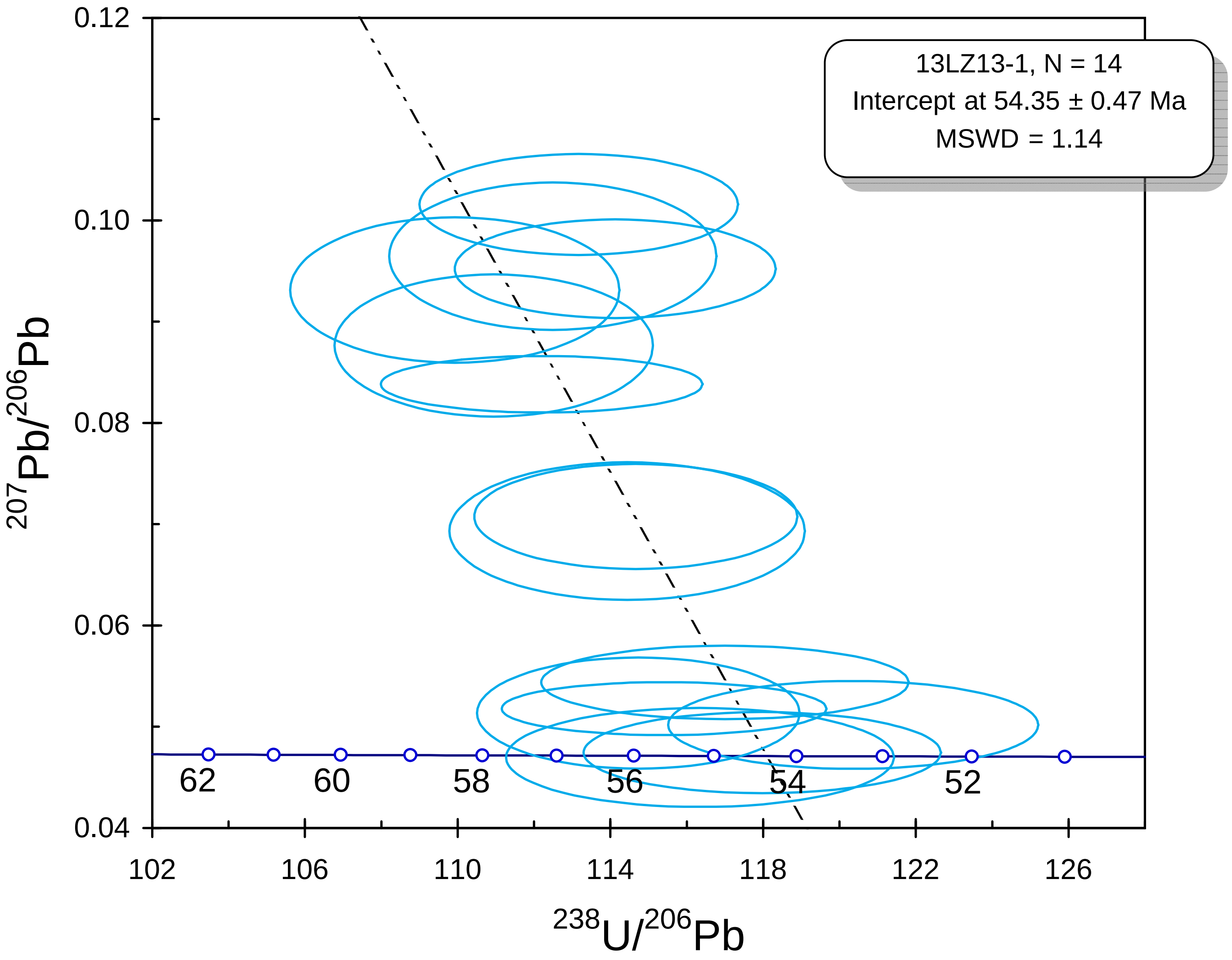

Concordia plots for single zircon analyzed by SIMS 1280 U–Pb dating

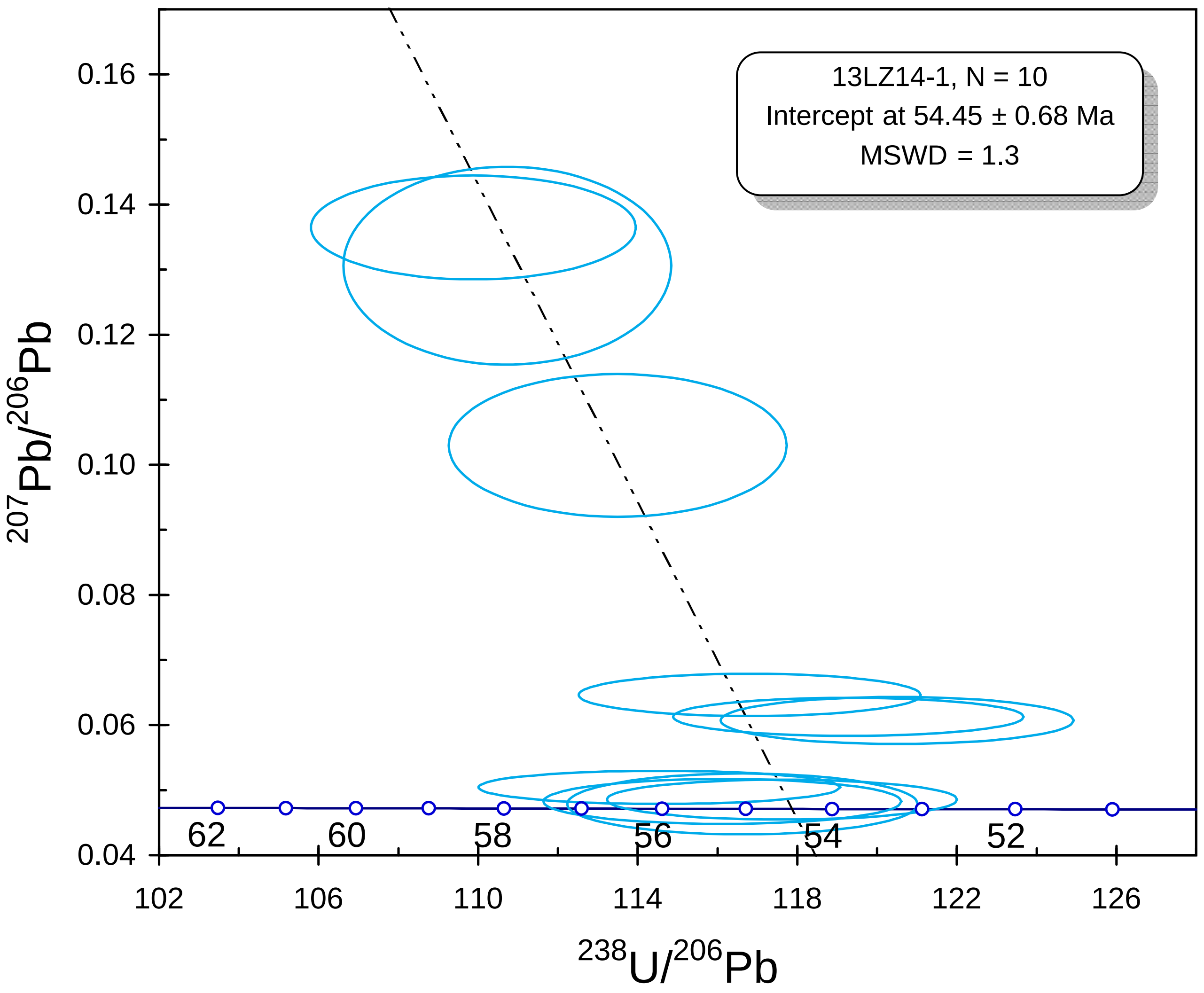

Concordia plots for single zircon analyzed by SIMS 1280 U–Pb dating

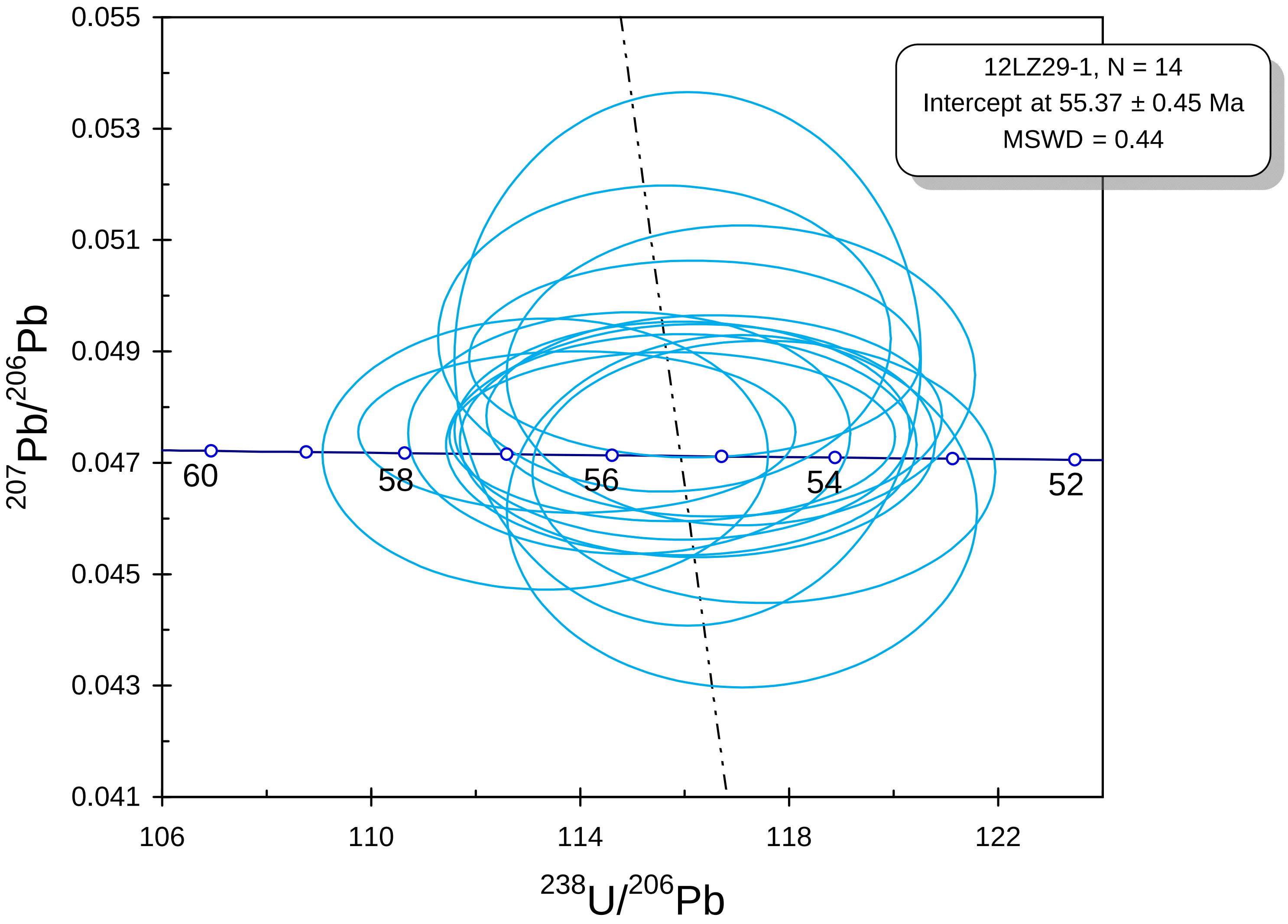

Concordia plots for single zircon analyzed by SIMS 1280 U–Pb dating

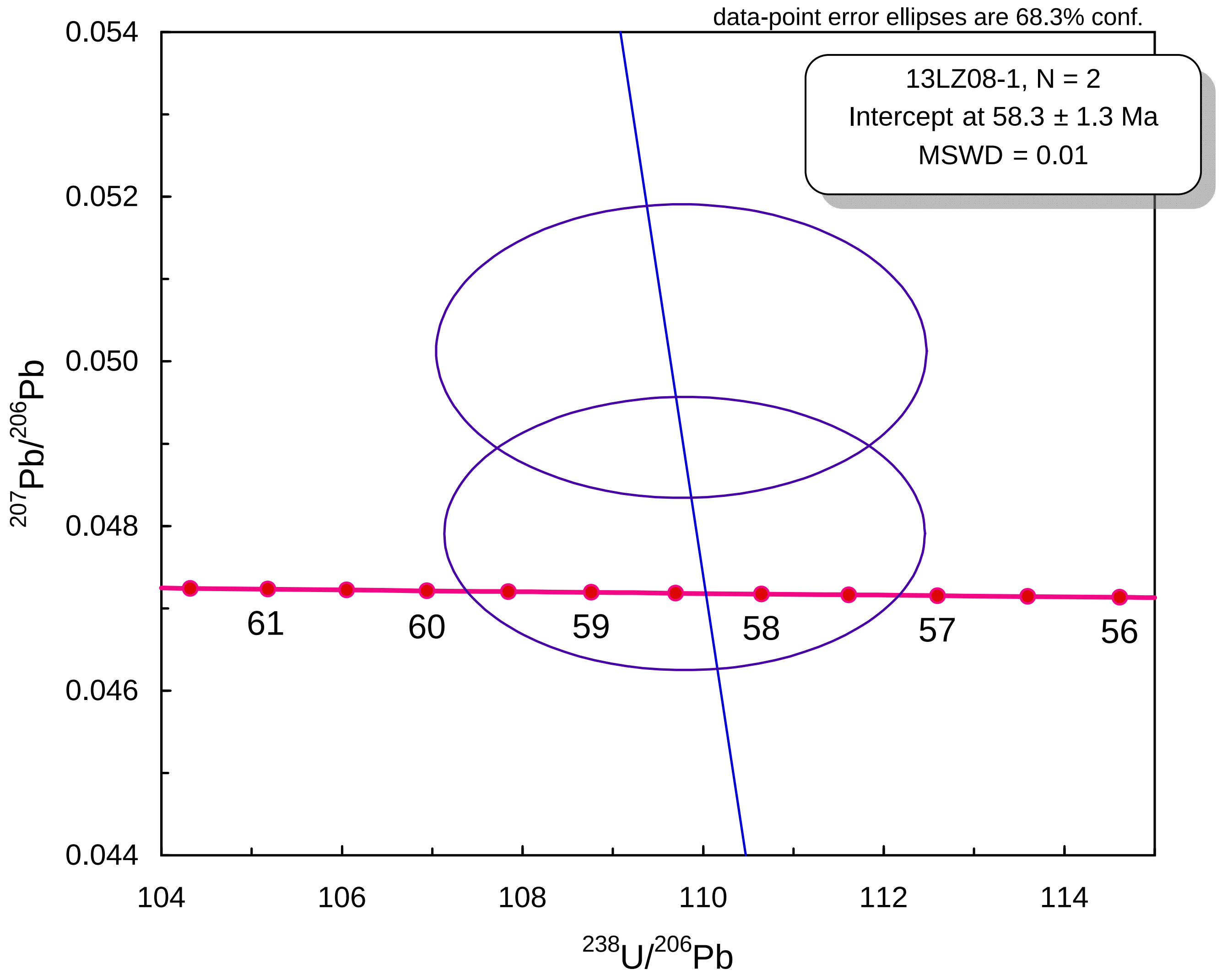

Concordia plots for single zircon analyzed by LA-ICPMS U–Pb dating

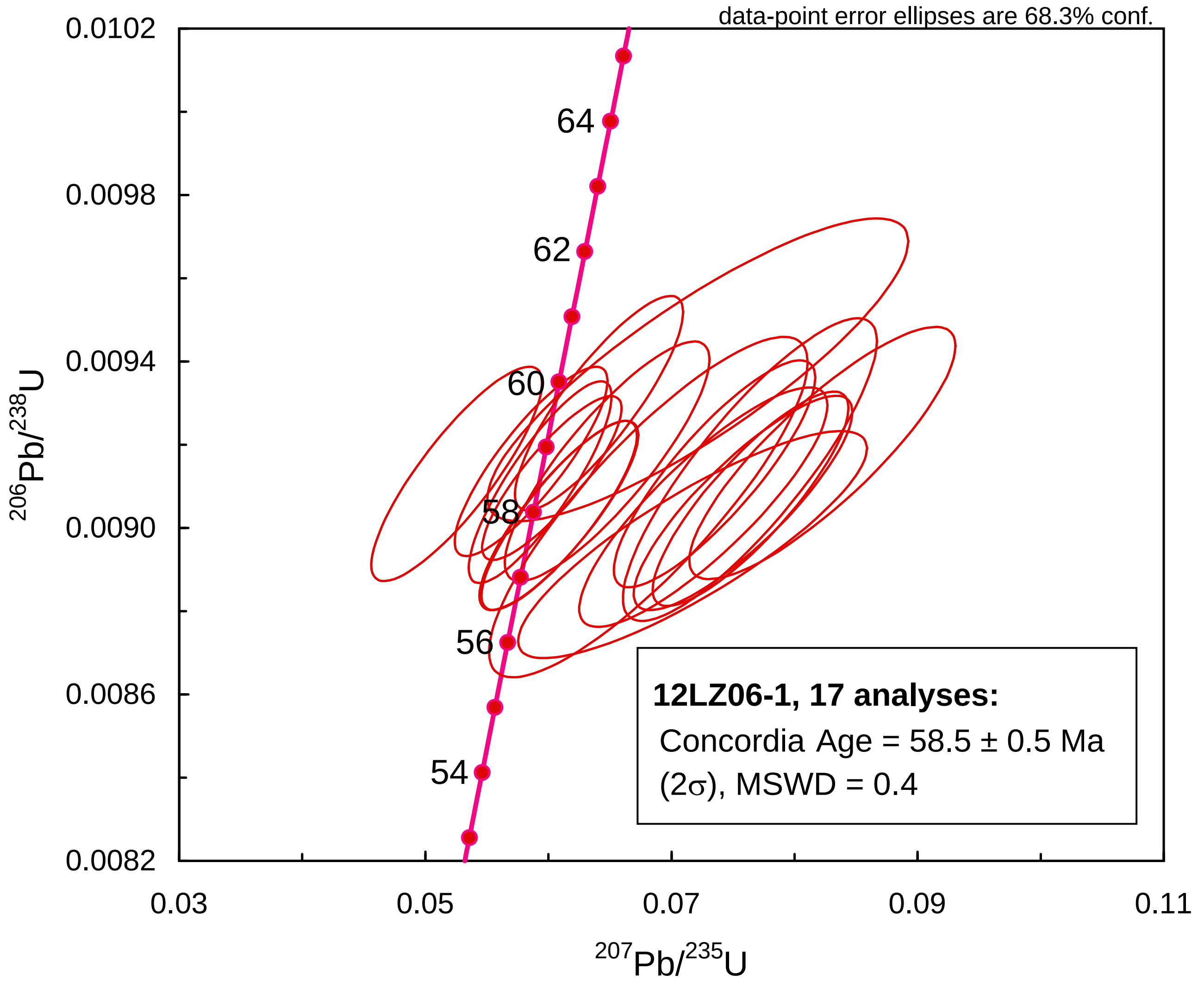

Concordia plots for single zircon analyzed by SIMS 1280 U–Pb dating

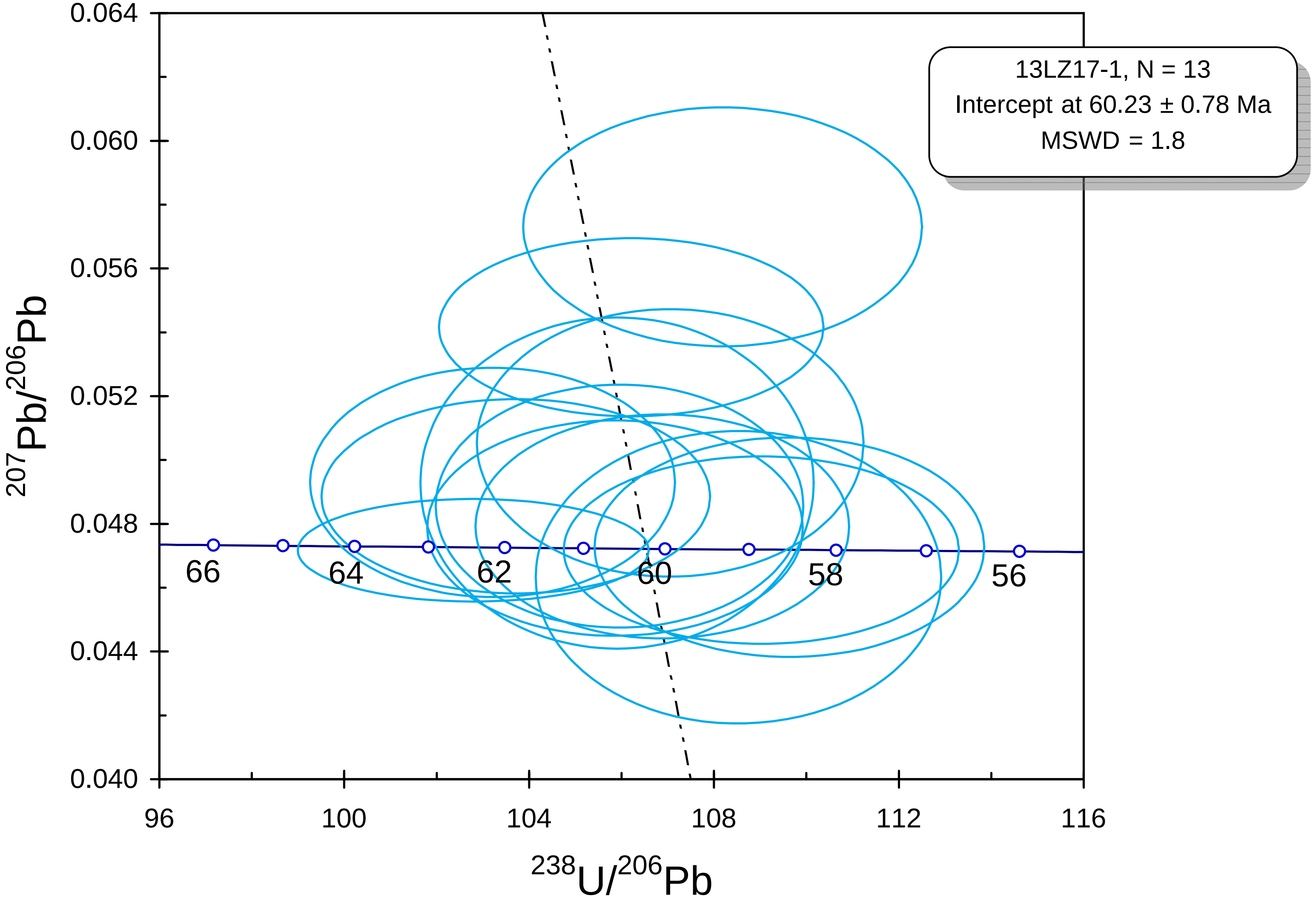

Concordia plots for single zircon analyzed by SIMS 1280 U–Pb dating

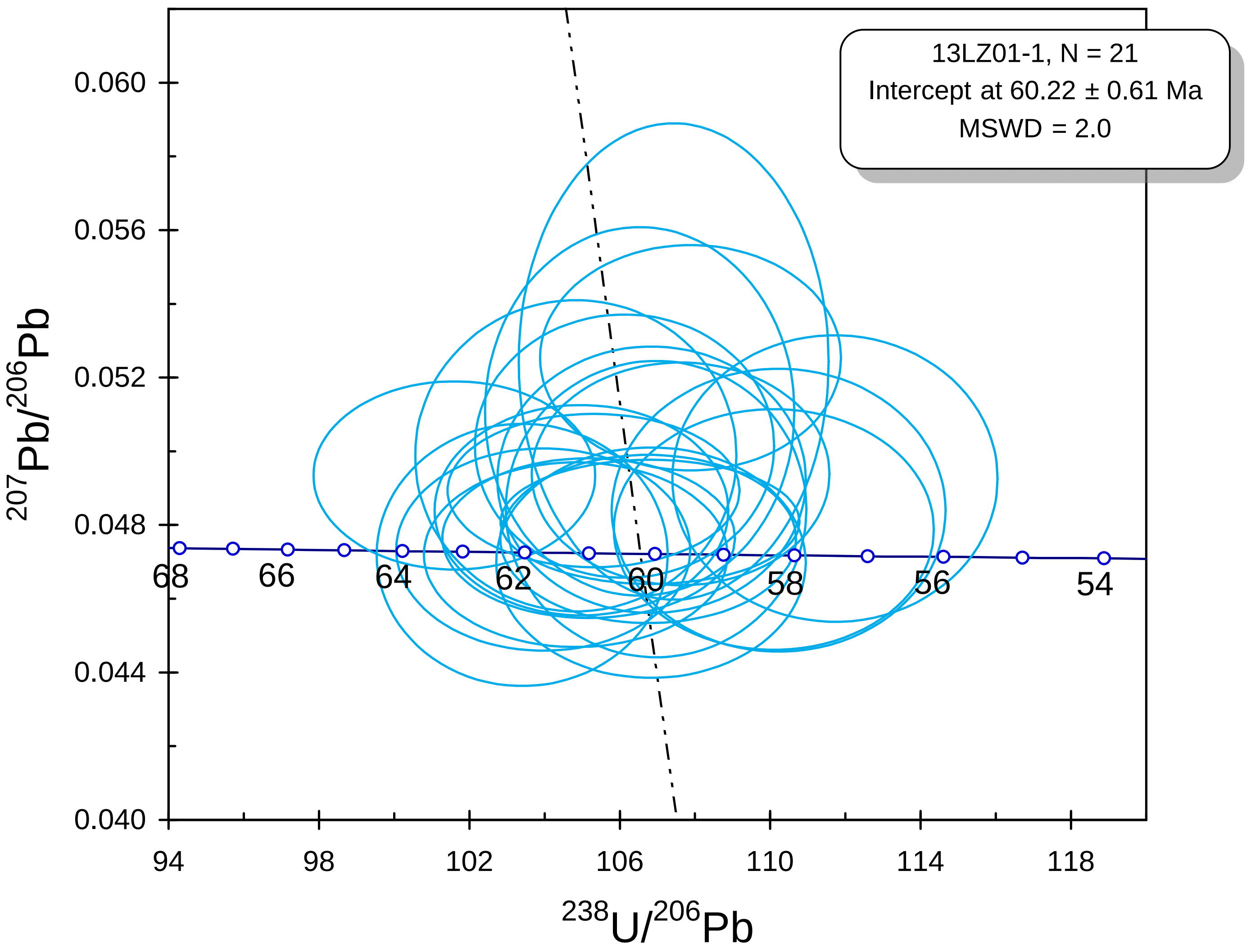

Concordia plots for single zircon analyzed by SIMS 1280 U–Pb dating

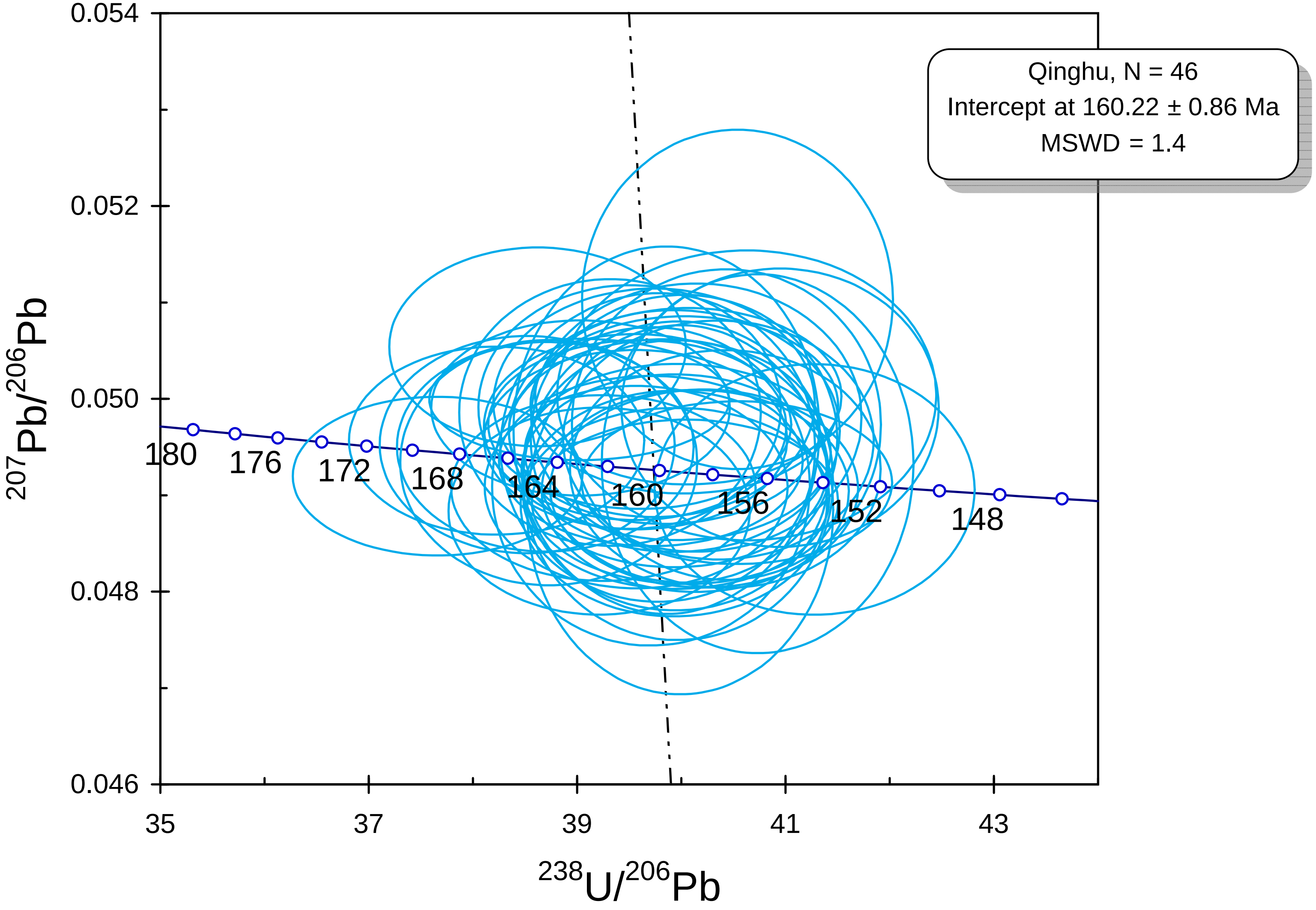

Magmatic record of India-Asia collision

Di-Cheng Zhu*, Qing Wang, Zhi-Dan Zhao, Sun-Lin Chung, Peter A. Cawood, Yaoling Niu, Sheng-Ao Liu, Fu-Yuan Wu, and Xuan-Xue Mo

Table S1 Zircon SIMS U-Pb age data reported in this study for the Linzizong volcanic rocks in Linzhou Basin

					TW concordia columns (Pbc uncorr.)					
**Sample/ spot #**	**[U] ppm**	**[Th] ppm**	**[Pb] ppm**	**Th/U meas**	$^{238}U/^{206}Pb$	**±σ %**	$^{207}Pb/^{206}Pb$	**±σ %**	**207-corr age (Ma)**	**±σ**
13LZ01-1@2	1717	1795	23	1.05	105.301	1.51	0.04894	1.74	60.8	0.9
13LZ01-1@4	475	324	6	0.68	107.433	1.57	0.05243	5.04	59.3	1.0
13LZ01-1@5	1268	1066	16	0.84	103.404	1.53	0.04719	3.08	62.0	1.0
13LZ01-1@6	1396	1487	19	1.06	103.966	1.54	0.04733	2.37	61.7	0.9
13LZ01-1@7	503	314	6	0.63	110.225	1.64	0.04840	3.24	58.1	1.0
13LZ01-1@8	812	715	10	0.88	107.618	1.50	0.04939	2.50	59.5	0.9
13LZ01-1@9	671	321	7	0.48	106.126	1.53	0.05014	2.91	60.2	0.9
13LZ01-1@10	695	491	8	0.71	110.097	1.58	0.04788	2.79	58.2	0.9
13LZ01-1@11	358	217	4	0.61	106.528	1.58	0.05108	4.00	59.9	1.0
13LZ01-1@12	906	918	12	1.01	106.829	1.57	0.04698	2.72	60.1	0.9
13LZ01-1@13	1076	1034	14	0.96	104.978	1.52	0.04840	2.41	61.0	0.9
13LZ01-1@15	889	1417	13	1.59	104.828	1.67	0.04988	3.46	61.0	1.0
13LZ01-1@16	1025	866	13	0.85	106.964	1.53	0.04843	3.39	59.9	0.9
13LZ01-1@17	925	1025	12	1.11	106.847	1.57	0.04923	3.00	59.9	0.9
13LZ01-1@18	2720	4708	41	1.73	106.807	1.52	0.04809	1.43	60.0	0.9
13LZ01-1@21	1643	2454	24	1.49	105.173	1.51	0.04765	1.86	61.0	0.9
13LZ01-1@22	1156	1387	15	1.20	107.880	1.52	0.05254	2.38	59.1	0.9
13LZ01-1@23	1347	1414	18	1.05	106.754	1.50	0.04762	1.96	60.1	0.9
13LZ01-1@24	1085	989	14	0.91	104.827	1.57	0.04719	2.17	61.2	1.0
13LZ01-1@1	644	366	7	0.57	111.719	1.58	0.04926	3.22	57.3	0.9
13LZ01-1@20	2663	4097	40	1.54	101.599	1.51	0.04934	2.11	63.0	0.9
~~13LZ01-1@14~~	~~1535~~	~~1750~~	~~18~~	~~1.14~~	~~114.101~~	~~6.37~~	~~0.05007~~	~~8.96~~	~~56.0~~	~~3.6~~
~~13LZ01-1@3~~	~~2194~~	~~1803~~	~~30~~	~~0.82~~	~~94.779~~	~~1.51~~	~~0.04766~~	~~1.48~~	~~67.6~~	~~1.0~~
~~13LZ01-1@19~~	~~3730~~	~~1639~~	~~210~~	~~0.44~~	~~21.015~~	~~1.50~~	~~0.05270~~	~~0.57~~	~~299.5~~	~~4.4~~
13LZ04-1@1	664	624	7	0.94	125.275	1.55	0.04769	1.97	51.2	0.8
13LZ04-1@2	814	811	9	1.00	125.571	1.50	0.04871	1.83	51.0	0.8
13LZ04-1@3	871	631	9	0.73	123.858	1.51	0.04797	2.97	51.8	0.8
13LZ04-1@4	1860	732	18	0.39	123.791	1.50	0.04872	1.15	51.8	0.8
13LZ04-1@5	1070	726	11	0.68	126.203	1.53	0.04709	1.52	50.9	0.8

13LZ04-1@6	868	710	9	0.82	122.853	1.50	0.04747	1.86	52.2	0.8
13LZ04-1@8	2269	1981	25	0.87	120.824	1.53	0.04820	0.99	53.1	0.8
13LZ04-1@9	739	437	7	0.59	124.374	1.53	0.04781	1.74	51.6	0.8
13LZ04-1@10	1663	3474	24	2.09	120.983	1.52	0.04735	1.45	53.0	0.8
13LZ04-1@11	918	640	9	0.70	125.435	1.53	0.04918	1.59	51.0	0.8
13LZ04-1@13	654	368	6	0.56	125.413	1.54	0.04840	2.01	51.1	0.8
13LZ04-1@18	2337	1661	24	0.71	121.479	1.53	0.04667	1.73	52.9	0.8
13LZ04-1@22	895	675	9	0.75	126.802	1.66	0.04708	3.62	50.6	0.8
13LZ04-1@19	2012	3024	26	1.50	119.980	1.53	0.04816	1.77	53.4	0.8
13LZ04-1@20	993	1350	12	1.36	123.281	1.55	0.04757	4.03	52.0	0.8
13LZ04-1@21	1030	886	11	0.86	122.823	1.56	0.05205	2.46	51.9	0.8
13LZ04-1@12	1523	1019	16	0.67	118.649	1.54	0.04758	1.23	54.1	0.8
13LZ04-1@14	1468	984	16	0.67	116.904	1.51	0.04610	1.52	55.0	0.8
13LZ04-1@15	347	260	4	0.75	117.612	1.79	0.04783	2.83	54.5	1.0
~~13LZ04-1@17~~	~~808~~	~~1065~~	~~9~~	~~1.32~~	~~121.052~~	~~1.56~~	~~0.06371~~	~~3.61~~	~~51.9~~	~~0.8~~
~~13LZ04-1@7~~	~~2258~~	~~3821~~	~~32~~	~~1.69~~	~~111.222~~	~~1.51~~	~~0.06321~~	~~1.81~~	~~56.5~~	~~0.9~~
~~13LZ04-1@16~~	~~3702~~	~~2849~~	~~35~~	~~0.77~~	~~126.016~~	~~1.50~~	~~0.07698~~	~~1.40~~	~~49.0~~	~~0.8~~
13LZ05-1@1	669	472	7	0.71	123.950	1.59	0.04715	3.78	51.8	0.8
13LZ05-1@2	3372	2482	35	0.74	121.663	1.51	0.04989	1.48	52.6	0.8
13LZ05-1@3	3570	6392	47	1.79	120.832	1.50	0.04695	1.47	53.1	0.8
13LZ05-1@4	1087	799	11	0.73	122.072	1.61	0.04930	3.20	52.4	0.8
13LZ05-1@5	2920	2191	32	0.75	117.553	1.53	0.04981	1.51	54.4	0.8
13LZ05-1@6	537	559	6	1.04	122.971	1.67	0.04658	3.50	52.2	0.9
13LZ05-1@7	1430	913	15	0.64	120.838	1.53	0.04447	2.57	53.3	0.8
13LZ05-1@8	1913	2980	25	1.56	119.378	1.53	0.04707	1.82	53.8	0.8
13LZ05-1@9	1116	766	11	0.69	122.124	1.56	0.04897	3.28	52.4	0.8
13LZ05-1@10	917	1318	11	1.44	123.856	1.52	0.04635	2.82	51.9	0.8
13LZ05-1@11	990	808	10	0.82	123.670	1.56	0.04731	2.73	51.9	0.8
13LZ05-1@12	4269	4381	48	1.03	121.921	1.50	0.04775	1.56	52.6	0.8
13LZ05-1@13	1188	948	13	0.80	119.315	1.56	0.04766	2.51	53.8	0.8
13LZ05-1@14	2508	1773	26	0.71	122.744	1.62	0.04921	1.76	52.2	0.8
13LZ05-1@17	1349	1271	14	0.94	124.881	1.62	0.05042	2.20	51.2	0.8
13LZ05-1@18	2333	2276	25	0.98	125.816	1.50	0.04744	2.56	51.0	0.8
~~13LZ05-1@15~~	~~1195~~	~~994~~	~~12~~	~~0.83~~	~~122.167~~	~~1.56~~	~~0.05991~~	~~2.19~~	~~51.7~~	~~0.8~~
~~13LZ05-1@16~~	~~730~~	~~505~~	~~7~~	~~0.69~~	~~126.497~~	~~1.60~~	~~0.06233~~	~~4.65~~	~~49.8~~	~~0.8~~

12LZ13-1@02	395	246	no data	0.62	112.493	1.56	0.09647	3.08	53.5	1.0
12LZ13-1@06	850	996	9	1.17	113.172	1.51	0.10158	2.00	52.8	1.0
12LZ13-1@16	829	636	8	0.77	114.128	1.51	0.09523	2.09	52.8	0.9
12LZ13-1@1	345	198	no data	0.57	114.440	1.66	0.06933	4.00	54.5	0.9
12LZ13-1@21	292	204	3	0.70	109.925	1.60	0.09313	3.15	55.0	1.0
12LZ13-1@23	895	616	10	0.69	112.201	1.53	0.08383	1.36	54.5	0.9
12LZ13-1@05	1272	1323	15	1.04	110.945	1.54	0.08766	3.27	54.9	0.9
12LZ13-1@14	497	351	5	0.71	114.666	1.51	0.07076	2.99	54.3	0.9
12LZ13-1@08	510	406	5	0.80	117.979	1.62	0.04745	3.44	54.4	0.9
12LZ13-1@17	318	251	4	0.79	117.003	1.68	0.05438	2.73	54.4	0.9
12LZ13-1@09	1319	1163	15	0.88	115.406	1.51	0.05179	2.07	55.3	0.8
12LZ13-1@15	526	427	6	0.81	114.732	1.50	0.05135	4.36	55.6	0.9
12LZ13-1@18	221	191	2	0.87	120.363	1.65	0.05019	3.54	53.1	0.9
12LZ13-1@20	209	159	2	0.76	116.348	1.78	0.04697	4.25	55.2	1.0
~~12LZ13-1@19~~	~~1301~~	~~1007~~	~~15~~	~~0.77~~	~~108.954~~	~~1.58~~	~~0.05203~~	~~1.37~~	~~58.5~~	~~0.9~~
~~12LZ13-1@07~~	~~3609~~	~~2187~~	~~40~~	~~0.61~~	~~110.109~~	~~1.51~~	~~0.04854~~	~~1.31~~	~~58.2~~	~~0.9~~
~~12LZ13-1@12~~	~~542~~	~~748~~	~~[5]~~	~~1.38~~	~~67.986~~	~~3.13~~	~~0.41662~~	~~4.29~~	~~49.9~~	~~6.3~~
~~12LZ13-1@11~~	~~628~~	~~466~~	~~6~~	~~0.74~~	~~68.966~~	~~4.92~~	~~0.37724~~	~~7.31~~	~~53.8~~	~~6.5~~
~~12LZ13-1@04~~	~~755~~	~~662~~	~~no data~~	~~0.88~~	~~91.045~~	~~1.64~~	~~0.26516~~	~~1.79~~	~~50.9~~	~~2.7~~
~~12LZ13-1@13~~	~~589~~	~~383~~	~~[5]~~	~~0.65~~	~~101.675~~	~~1.50~~	~~0.20308~~	~~1.65~~	~~50.6~~	~~1.8~~
~~12LZ13-1@22~~	~~799~~	~~601~~	~~14~~	~~0.75~~	~~73.219~~	~~1.53~~	~~0.05134~~	~~1.42~~	~~87.0~~	~~1.3~~
~~12LZ13-1@10~~	~~821~~	~~539~~	~~14~~	~~0.66~~	~~67.910~~	~~1.51~~	~~0.09360~~	~~2.80~~	~~88.8~~	~~1.5~~
~~12LZ13-1@03~~	~~587~~	~~878~~	~~13~~	~~1.50~~	~~68.491~~	~~1.52~~	~~0.05427~~	~~2.48~~	~~92.7~~	~~1.4~~
12LZ14-1@02	1011	333	no data	0.33	109.879	1.51	0.13648	2.39	51.8	1.2
12LZ14-1@07	798	526	8	0.66	110.730	1.52	0.13059	4.76	51.8	1.2
12LZ14-1@15	1475	805	14	0.55	120.503	1.50	0.06070	2.43	52.4	0.8
12LZ14-1@16	904	649	9	0.72	113.498	1.53	0.10297	4.35	52.5	1.0
12LZ14-1@13	1959	961	18	0.49	119.280	1.50	0.06127	1.95	52.8	0.8
12LZ14-1@10	1538	851	15	0.55	116.805	1.50	0.06463	2.06	53.7	0.8
12LZ14-1@12	1067	346	10	0.32	117.616	1.53	0.04854	2.58	54.5	0.8
12LZ14-1@1	828	423	9	0.51	116.628	1.54	0.04786	3.99	55.0	0.9
12LZ14-1@14	853	1460	11	1.71	116.127	1.58	0.04826	2.92	55.2	0.9
12LZ14-1@03	1844	1205	19	0.65	114.543	1.62	0.05044	2.07	55.8	0.9
~~12LZ14-1@11~~	~~420~~	~~227~~	~~[2]~~	~~0.54~~	~~92.538~~	~~4.46~~	~~0.55822~~	~~4.34~~	~~24.1~~	~~6.3~~
~~12LZ14-1@05~~	~~1592~~	~~1292~~	~~[12]~~	~~0.81~~	~~119.331~~	~~1.88~~	~~0.28164~~	~~1.68~~	~~37.7~~	~~2.2~~
~~12LZ14-1@08~~	~~885~~	~~362~~	~~[7]~~	~~0.41~~	~~100.858~~	~~2.42~~	~~0.29904~~	~~3.15~~	~~43.2~~	~~2.9~~
~~12LZ14-1@09~~	~~790~~	~~707~~	~~no data~~	~~0.89~~	~~113.755~~	~~1.50~~	~~0.20301~~	~~3.30~~	~~45.2~~	~~1.7~~

~~12LZ14-1@04~~	~~1792~~	~~1497~~	~~16~~	~~0.84~~	~~95.384~~	~~1.60~~	~~0.26339~~	~~3.00~~	~~48.7~~	~~2.6~~
~~12LZ14-1@06~~	~~763~~	~~217~~	~~48~~	~~0.28~~	~~17.857~~	~~1.54~~	~~0.05584~~	~~1.26~~	~~350.2~~	~~5.3~~
**13LZ16-1@1**	437	659	6	1.51	118.043	1.55	0.04861	2.09	54.3	0.8
**13LZ16-1@15**	705	776	8	1.10	123.590	1.52	0.04791	1.65	51.9	0.8
**13LZ16-1@16**	436	534	5	1.22	123.823	1.53	0.04770	3.37	51.8	0.8
**13LZ16-1@17**	205	156	2	0.76	120.018	1.64	0.05612	3.30	52.9	0.9
13LZ16-1@4	625	292	8	0.47	93.635	1.53	0.04849	1.55	68.4	1.0
13LZ16-1@5	233	295	4	1.27	92.295	1.62	0.04772	2.64	69.4	1.1
13LZ16-1@6	975	852	13	0.87	97.197	1.67	0.04870	1.31	65.9	1.1
13LZ16-1@7	489	194	6	0.40	92.893	1.51	0.04717	1.77	69.0	1.0
13LZ16-1@8	915	772	13	0.84	93.934	1.51	0.04711	1.95	68.3	1.0
13LZ16-1@10	1380	1471	20	1.07	93.985	1.51	0.04678	1.18	68.3	1.0
13LZ16-1@13	665	436	9	0.66	95.934	1.50	0.04764	2.65	66.8	1.0
13LZ16-1@14	725	342	9	0.47	95.731	1.51	0.04975	1.43	66.8	1.0
13LZ16-1@18	498	226	7	0.45	91.134	1.50	0.04751	1.73	70.3	1.1
~~13LZ16-1@3~~	~~346~~	~~201~~	4	~~0.58~~	~~97.425~~	~~1.59~~	~~0.05074~~	~~2.08~~	~~65.5~~	~~1.0~~
~~13LZ16-1@11~~	~~1258~~	~~1281~~	~~18~~	~~1.02~~	~~95.173~~	~~1.51~~	~~0.05073~~	~~1.48~~	~~67.1~~	~~1.0~~
~~13LZ16-1@2~~	~~1421~~	~~2137~~	~~23~~	~~1.50~~	~~88.800~~	~~1.74~~	~~0.06841~~	~~17.99~~	~~70.3~~	~~1.7~~
~~13LZ16-1@12~~	~~564~~	~~359~~	~~6~~	~~0.64~~	~~96.311~~	~~2.00~~	~~0.10216~~	~~4.62~~	~~61.9~~	~~1.4~~
~~13LZ16-1@9~~	~~435~~	~~861~~	~~8~~	~~1.98~~	~~58.047~~	~~5.33~~	~~0.32544~~	~~10.40~~	~~71.2~~	~~7.9~~
13LZ17-1@01	306	166	3	0.54	109.633	1.57	0.04727	2.97	58.5	0.9
13LZ17-1@04	298	168	3	0.56	108.188	1.63	0.05731	2.67	58.5	1.0
13LZ17-1@11	429	302	5	0.70	109.025	1.60	0.04718	2.54	58.9	0.9
13LZ17-1@07	176	83	2	0.47	108.529	1.65	0.04633	4.04	59.2	1.0
13LZ17-1@05	229	123	3	0.54	107.053	1.60	0.05054	3.39	59.7	1.0
13LZ17-1@16	600	443	7	0.74	106.210	1.60	0.05416	2.10	59.9	1.0
13LZ17-1@02	402	321	5	0.80	106.882	1.55	0.04792	2.99	60.0	0.9
13LZ17-1@13	198	134	2	0.68	105.902	1.64	0.04928	4.30	60.4	1.0
13LZ17-1@14	755	583	9	0.77	105.957	1.53	0.04856	3.20	60.5	0.9
13LZ17-1@09	407	173	5	0.42	105.860	1.57	0.04787	2.88	60.6	1.0
13LZ17-1@17	465	219	5	0.47	103.713	1.66	0.04886	2.54	61.7	1.0
13LZ17-1@20	324	135	4	0.42	103.210	1.56	0.04930	2.98	62.0	1.0
13LZ17-1@10	1418	1064	18	0.75	102.794	1.51	0.04717	1.39	62.4	0.9
~~13LZ17-1@19~~	~~100~~	~~66~~	~~2~~	~~0.66~~	~~61.630~~	~~1.62~~	~~0.04618~~	~~4.28~~	~~104.0~~	~~1.7~~
~~13LZ17-1@08~~	~~394~~	~~192~~	~~8~~	~~0.49~~	~~60.541~~	~~1.54~~	~~0.04885~~	~~2.11~~	~~105.5~~	~~1.6~~
~~13LZ17-1@12~~	~~121~~	~~168~~	4	~~1.39~~	~~51.491~~	~~1.56~~	~~0.04850~~	~~3.93~~	~~124.0~~	~~1.9~~

~~13LZ17-1@06~~	~~375~~	~~213~~	~~9~~	~~0.57~~	~~49.593~~	~~1.52~~	~~0.05080~~	~~1.70~~	~~128.3~~	~~1.9~~
~~13LZ17-1@18~~	~~196~~	~~131~~	~~6~~	~~0.67~~	~~40.695~~	~~1.53~~	~~0.04909~~	~~3.00~~	~~156.5~~	~~2.4~~
~~13LZ17-1@03~~	~~349~~	~~133~~	~~20~~	~~0.38~~	~~20.901~~	~~1.50~~	~~0.05267~~	~~1.13~~	~~301.2~~	~~4.5~~
~~13LZ17-1@15~~	~~1074~~	~~742~~	~~67~~	~~0.69~~	~~20.313~~	~~1.50~~	~~0.05213~~	~~0.71~~	~~310.0~~	~~4.6~~
12LZ25-1@16	1255	1526	15	1.22	122.992	1.52	0.04882	1.46	52.1	0.8
12LZ25-1@15	522	531	6	1.02	121.911	1.59	0.04644	2.86	52.7	0.8
12LZ25-1@14	512	515	6	1.01	123.722	1.54	0.04968	3.61	51.7	0.8
12LZ25-1@13	619	732	7	1.18	123.955	1.56	0.04865	2.31	51.7	0.8
12LZ25-1@08	474	518	5	1.09	121.635	1.71	0.06249	3.61	51.7	0.9
12LZ25-1@11	1361	2233	18	1.64	122.104	1.59	0.04730	1.51	52.6	0.8
12LZ25-1@10	3176	870	30	0.27	119.616	1.59	0.04739	0.99	53.6	0.9
12LZ25-1@07	348	315	4	0.91	122.554	1.66	0.05012	2.72	52.2	0.9
12LZ25-1@06	857	863	10	1.01	119.582	1.58	0.04643	2.04	53.7	0.9
12LZ25-1@03	928	1209	11	1.30	122.673	1.54	0.05649	3.16	51.7	0.8
12LZ25-1@05	356	576	4	1.62	122.686	1.50	0.05881	4.46	51.5	0.8
12LZ25-1@04	1290	1797	15	1.39	122.518	1.57	0.05530	2.74	51.9	0.8
12LZ25-1@1	805	1206	9	1.50	122.838	1.70	0.04802	3.12	52.2	0.9
~~12LZ25-1@09~~	~~1830~~	~~1943~~	~~22~~	~~1.06~~	~~115.528~~	~~1.60~~	~~0.06726~~	~~3.53~~	~~54.1~~	~~0.9~~
~~12LZ25-1@02~~	~~687~~	~~1052~~	~~8~~	~~1.53~~	~~126.135~~	~~1.54~~	~~0.05114~~	~~3.32~~	~~50.6~~	~~0.8~~
~~12LZ25-1@12~~	~~580~~	~~767~~	~~7~~	~~1.32~~	~~119.202~~	~~1.58~~	~~0.06250~~	~~9.57~~	~~52.8~~	~~0.9~~
12LZ27-1@15	648	532	7	0.82	122.775	1.56	0.04874	2.07	52.2	0.8
12LZ27-1@14	346	396	4	1.15	123.629	1.58	0.04870	2.88	51.8	0.8
12LZ27-1@13	1540	1124	17	0.73	120.481	1.52	0.04680	1.69	53.3	0.8
12LZ27-1@12	1003	624	11	0.62	120.158	1.52	0.04632	1.71	53.5	0.8
12LZ27-1@11	1547	1586	18	1.03	119.830	1.53	0.04772	1.58	53.5	0.8
12LZ27-1@10	864	888	10	1.03	121.922	1.53	0.04837	1.84	52.6	0.8
12LZ27-1@09	627	748	8	1.19	122.723	1.62	0.04799	2.06	52.3	0.8
12LZ27-1@08	598	725	7	1.21	120.978	1.54	0.04664	2.13	53.1	0.8
12LZ27-1@07	1318	947	14	0.72	123.416	1.67	0.04701	1.45	52.0	0.9
12LZ27-1@06	683	525	7	0.77	121.753	1.57	0.04773	1.98	52.7	0.8
12LZ27-1@05	610	677	7	1.11	122.872	1.55	0.04913	2.11	52.1	0.8
12LZ27-1@04	643	802	8	1.25	122.039	1.55	0.04849	2.07	52.5	0.8
12LZ27-1@03	1472	942	15	0.64	121.065	1.51	0.04864	1.35	52.9	0.8
12LZ27-1@02	228	167	2	0.73	123.920	1.65	0.04734	3.49	51.8	0.9
12LZ27-1@1	981	997	11	1.02	121.556	1.53	0.04728	1.70	52.8	0.8
~~12LZ27-1@17~~	~~159~~	~~114~~	~~2~~	~~0.72~~	~~126.116~~	~~1.73~~	~~0.04523~~	~~4.39~~	~~51.0~~	~~0.9~~

~~12LZ27-1@16~~	~~162~~	~~119~~	~~2~~	~~0.74~~	~~119.454~~	~~1.67~~	~~0.04604~~	~~4.71~~	~~53.8~~	~~0.9~~
12LZ29-1@07	1926	982	21	0.51	113.334	1.54	0.04716	2.11	56.6	0.9
12LZ29-1@05	2185	2082	26	0.95	113.938	1.50	0.04755	1.25	56.3	0.8
12LZ29-1@08	1466	1067	16	0.73	114.938	1.50	0.04753	1.86	55.8	0.8
12LZ29-1@1	1955	1656	22	0.85	115.763	1.50	0.04747	1.31	55.4	0.8
12LZ29-1@04	1277	1822	17	1.43	115.611	1.53	0.04923	2.28	55.4	0.8
12LZ29-1@14	887	576	10	0.65	115.937	1.59	0.04732	1.71	55.4	0.9
12LZ29-1@13	934	538	10	0.58	115.953	1.54	0.04758	1.68	55.3	0.8
12LZ29-1@02	1013	568	11	0.56	116.249	1.60	0.04740	1.80	55.2	0.9
12LZ29-1@10	386	190	4	0.49	116.057	1.57	0.04887	4.00	55.2	0.9
12LZ29-1@06	1369	1036	15	0.76	116.190	1.52	0.04886	1.48	55.1	0.8
12LZ29-1@11	1131	741	12	0.66	116.566	1.53	0.04784	1.54	55.0	0.8
12LZ29-1@12	500	334	5	0.67	117.096	1.57	0.04613	2.80	54.9	0.9
12LZ29-1@03	621	364	7	0.59	117.074	1.56	0.04857	2.26	54.7	0.9
12LZ29-1@15	840	785	10	0.94	117.515	1.54	0.04684	2.05	54.6	0.8
~~12LZ29-1@09~~	~~830~~	~~844~~	~~12~~	~~1.02~~	~~97.539~~	~~1.51~~	~~0.04685~~	~~1.74~~	~~65.8~~	~~1.0~~
13LZ08-1@1	525	391	6	0.74	109.796	1.60	0.04791	2.29	57.9	0.9
13LZ08-1@03	352	262	4	0.74	109.759	1.63	0.05012	2.35	57.7	0.9
13LZ08-1@04	1498	834	20	0.56	92.549	1.62	0.04959	1.02	68.5	1.1
13LZ08-1@12	1797	1518	25	0.85	93.967	1.57	0.05263	1.93	67.8	1.1
13LZ08-1@07	741	936	34	1.26	31.315	1.50	0.05109	1.02	200.6	3.0
13LZ08-1@06	2667	1033	100	0.39	30.949	1.50	0.05093	0.44	203.0	3.0
13LZ08-1@11	927	1094	42	1.18	31.127	1.50	0.05120	0.79	201.8	3.0
13LZ08-1@08	2573	1039	147	0.40	20.533	1.52	0.05248	0.37	303.9	4.6
13LZ08-1@10	935	468	221	0.501	5.236	1.59	0.08027	0.45	1112.7	17.1

					TW concordia columns (Pbc uncorr.)					
**Sample/ spot #**	**[U] ppm**	**[Th] ppm**	**[Pb] ppm**	**Th/U meas**	**$^{238}U/^{206}Pb$**	**±σ %**	**$^{207}Pb/^{206}Pb$**	**±σ %**	**207-corr age (Ma)**	**±σ**
Qinghu@1	561	321	17	0.57	41.280	1.52	0.04906	1.08	154.3	2.3
Qinghu@2	1065	389	31	0.37	39.561	1.50	0.04958	0.77	160.9	2.4
~~Qinghu@3~~	~~1462~~	~~653~~	~~44~~	~~0.45~~	~~39.164~~	~~1.50~~	~~0.05711~~	~~0.67~~	~~160.9~~	~~2.4~~
Qinghu@4	2587	1458	81	0.56	39.021	1.51	0.04999	0.51	163.0	2.4
Qinghu@5	1132	320	32	0.28	39.600	1.50	0.04973	0.72	160.7	2.4
Qinghu@11	1480	612	44	0.41	39.952	1.50	0.04897	0.78	159.4	2.4

Qinghu@10	1334	699	42	0.52	39.253	1.52	0.04907	0.80	162.2	2.4
Qinghu@8	1344	536	40	0.40	39.556	1.50	0.04971	0.79	160.9	2.4
Qinghu@7	1043	410	31	0.39	39.928	1.51	0.04891	0.98	159.5	2.4
Qinghu@6	2441	1383	74	0.57	39.857	1.51	0.04967	1.57	159.7	2.4
Qinghu@5	2309	1351	70	0.58	40.145	1.61	0.04981	1.14	158.5	2.5
Qinghu@4	1733	837	52	0.48	39.708	1.57	0.04909	1.37	160.4	2.5
Qinghu@3	1664	901	51	0.54	39.943	1.51	0.04921	1.16	159.4	2.4
Qinghu@2	1139	673	34	0.59	40.539	1.50	0.05103	1.41	156.7	2.3
Qinghu@1	1643	803	49	0.49	40.629	1.83	0.05004	1.23	156.6	2.8
Qinghu@1	1836	1083	59	0.59	38.228	1.52	0.04957	0.80	166.4	2.5
Qinghu@3	1743	757	53	0.43	39.032	1.50	0.04990	0.74	163.0	2.4
~~Qinghu@4~~	~~830~~	~~352~~	~~25~~	~~0.42~~	~~37.426~~	~~1.51~~	~~0.08969~~	~~1.30~~	~~161.3~~	~~2.7~~
Qinghu@5	1543	575	45	0.37	39.566	1.50	0.04908	0.87	160.9	2.4
Qinghu@6	1680	798	52	0.47	38.726	1.50	0.04934	1.05	164.3	2.5
Qinghu@7	2310	1137	72	0.49	38.521	1.50	0.04952	0.93	165.2	2.5
Qinghu@8	1751	770	53	0.44	38.694	1.50	0.04950	0.90	164.4	2.5
Qinghu@18	1238	462	36	0.37	40.219	1.50	0.04907	0.86	158.4	2.4
Qinghu@17	1447	587	42	0.41	40.534	1.50	0.04913	0.70	157.1	2.3
Qinghu@16	1801	772	54	0.43	40.036	1.50	0.04893	0.71	159.1	2.4
Qinghu@15	689	279	20	0.41	40.422	1.51	0.04927	1.02	157.5	2.4
Qinghu@14	2473	1854	92	0.75	37.660	1.51	0.04920	0.68	169.0	2.5
Qinghu@13	1764	613	52	0.35	39.708	1.50	0.04935	0.73	160.3	2.4
Qinghu@12	1414	576	41	0.41	40.067	1.50	0.05003	0.75	158.8	2.4
Qinghu@11	1880	962	57	0.51	39.965	1.50	0.04945	0.76	159.3	2.4
Qinghu@10	1320	500	39	0.38	39.931	1.50	0.04925	0.83	159.4	2.4
Qinghu@9	1500	704	45	0.47	40.028	1.51	0.04994	0.75	158.9	2.4
Qinghu@8	1228	494	36	0.40	40.134	1.50	0.04904	0.87	158.7	2.4
Qinghu@7	1676	826	51	0.49	39.834	1.53	0.04966	0.79	159.8	2.4
Qinghu@6	1206	743	38	0.62	39.739	1.59	0.04996	0.97	160.1	2.5
Qinghu@5	1727	971	54	0.56	39.211	1.51	0.04884	0.90	162.4	2.4
Qinghu@4	1094	590	33	0.54	40.356	1.51	0.04957	1.02	157.7	2.4
Qinghu@3	1068	454	31	0.42	40.432	1.50	0.04973	1.32	157.4	2.3
Qinghu@2	1285	679	40	0.53	39.316	1.50	0.04986	1.13	161.8	2.4
Qinghu@1	1164	548	35	0.47	40.026	1.50	0.04959	1.23	159.0	2.4
Qinghu@1	1139	609	35	0.54	39.934	1.62	0.04966	1.03	159.4	2.6
Qinghu@2	2065	905	62	0.44	39.775	1.55	0.04949	1.32	159.6	2.5
Qinghu@3	1948	908	58	0.47	39.989	1.50	0.04885	1.60	158.9	2.4
Qinghu@4	1790	887	52	0.50	40.947	1.52	0.04991	1.18	155.0	2.3

Qinghu@5	736	398	22	0.54	40.732	1.50	0.04933	1.63	155.9	2.3
Qinghu@6	1831	887	55	0.48	39.504	1.50	0.04991	1.04	160.6	2.4
Qinghu@7	2494	1238	78	0.50	38.620	1.51	0.05054	0.83	164.1	2.5
~~Qinghu@8~~	~~735~~	~~332~~	~~22~~	~~0.45~~	~~39.949~~	~~1.53~~	~~0.05475~~	~~1.83~~	~~157.8~~	~~2.4~~
Qinghu@9	1088	356	31	0.33	39.969	1.52	0.04915	1.37	158.9	2.4

Note: Qinghu represents external standardization (Li et al., 2009) during zircon SIMS U-Pb dating

Li, X.H. et al. Precise determination of Phanerozoic zircon Pb/Pb age by multicollector SIMS without external standardization. Geochem. Geophys. Geosyst. 10, Q04010, doi: 10.1029/2009GC002400 (2009).

Magmatic record of India-Asia collision

Di-Cheng Zhu*, Qing Wang, Zhi-Dan Zhao, Sun-Lin Chung, Peter A. Cawood, Yaoling Niu, Sheng-Ao Liu, Fu-Yuan Wu, and Xuan-Xue Mo

Table S2 Zircon LA-ICPMS U-Pb age data reported in this study for the Linzizong volcanic rocks in Linzhou Basin

	CORRECTED RATIOS										CORRECTED AGES (Ma)							
Analyses	$^{207}Pb/^{206}Pb$	1σ	$^{207}Pb/^{235}U$	1σ	$^{206}Pb/^{238}U$	1σ	$^{208}Pb/^{232}Th$	1σ	$^{238}U/^{232}Th$	1σ	$^{207}Pb/^{206}Pb$	1σ	$^{207}Pb/^{235}U$	1σ	$^{206}Pb/^{238}U$	1σ	$^{208}Pb/^{232}Th$	1σ
12LZ06-1-01	0.04161	0.00341	0.05255	0.00458	0.00913	0.00017	0.00289	0.00010	1.03	0.01	-198	142	52	4	59	1	58	2
12LZ06-1-02	0.05038	0.00365	0.06085	0.00426	0.00903	0.00015	0.00281	0.00009	0.91	0.01	213	128	60	4	58	1	57	2
12LZ06-1-03	0.05813	0.00441	0.07350	0.00540	0.00913	0.00018	0.00271	0.00012	1.19	0.01	535	128	72	5	59	1	55	2
12LZ06-1-04	0.06786	0.00693	0.08227	0.00714	0.00918	0.00020	0.00290	0.00013	1.07	0.01	864	145	80	7	59	1	58	3
12LZ06-1-05	0.06452	0.00681	0.07259	0.00666	0.00905	0.00019	0.00271	0.00015	1.43	0.01	759	160	71	6	58	1	55	3
12LZ06-1-06	0.05575	0.00884	0.07214	0.01129	0.00938	0.00024	0.00292	0.00006	1.22	0.01	442	348	71	11	60	2	59	1
12LZ06-1-07	0.05250	0.00563	0.06812	0.00855	0.00905	0.00027	0.00325	0.00022	0.92	0.01	307	228	67	8	58	2	66	5
12LZ06-1-08	0.05209	0.00448	0.06477	0.00549	0.00916	0.00019	0.00287	0.00013	1.18	0.01	289	155	64	5	59	1	58	3
12LZ06-1-09	0.05129	0.00414	0.06088	0.00417	0.00903	0.00015	0.00286	0.00011	1.14	0.01	254	127	60	4	58	0.9	58	2
12LZ06-1-10	0.06441	0.00580	0.07637	0.00681	0.00914	0.00024	0.00300	0.00020	1.8	0.02	755	145	75	6	59	2	61	4
12LZ06-1-11	0.04709	0.00336	0.05859	0.00410	0.00916	0.00015	0.00271	0.00011	1.25	0.01	54	122	58	4	58.8	1	55	2
12LZ06-1-12	0.04732	0.00281	0.06028	0.00374	0.00912	0.00013	0.00278	0.00010	1.45	0.01	65	109	59	4	58.5	0.8	56	2
12LZ06-1-13	0.05215	0.00389	0.06412	0.00451	0.00930	0.00017	0.00308	0.00012	1.18	0.01	292	128	63	4	60	1	62	2
~~12LZ06-1-14~~	~~0.04850~~	~~0.00173~~	~~0.32266~~	~~0.01139~~	~~0.04792~~	~~0.00052~~	~~0.01439~~	~~0.00044~~	~~2.97~~	~~0.03~~	~~124~~	~~62~~	~~284~~	~~9~~	~~302~~	~~3~~	~~289~~	~~9~~
12LZ06-1-15	0.04830	0.00323	0.05933	0.00383	0.00911	0.00016	0.00277	0.00010	1.04	0.01	114	112	59	4	58	1	56	2
12LZ06-1-16	0.06358	0.00547	0.07582	0.00586	0.00906	0.00017	0.00290	0.00012	0.99	0.01	728	133	74	6	58	1	59	2
12LZ06-1-17	0.06301	0.00442	0.07642	0.00525	0.00907	0.00017	0.00285	0.00013	1.09	0.01	708	115	75	5	58	1	57	3
12LZ06-1-18	0.05802	0.00767	0.07171	0.00936	0.00896	0.00018	0.00278	0.00004	1.62	0.02	531	301	70	9	58	1	56.1	0.9
~~12LZ23-1-01~~	~~0.04719~~	~~0.00508~~	~~0.04100~~	~~0.00438~~	~~0.00630~~	~~0.00009~~	~~0.00200~~	~~0.00009~~	~~1.41~~	~~0.01~~	~~59~~	~~226~~	~~41~~	4	~~40.5~~	~~0.5~~	~~40~~	~~2~~
12LZ23-1-02	0.05175	0.00591	0.05455	0.00574	0.00772	0.00017	0.00199	0.00013	1.6	0.02	275	196	54	6	50	1	40	3
12LZ23-1-03	0.04463	0.00226	0.04750	0.00243	0.00768	0.00010	0.00242	0.00007	1.51	0.02	-38	82	47	2	49.3	0.7	49	2
12LZ23-1-04	0.04795	0.00303	0.05162	0.00329	0.00781	0.00011	0.00267	0.00010	1.91	0.02	97	114	51	3	50.1	0.7	54	2
12LZ23-1-05	0.04927	0.00337	0.05168	0.00338	0.00773	0.00013	0.00258	0.00012	1.95	0.02	161	116	51	3	49.7	0.8	52	2
12LZ23-1-06	0.05853	0.00415	0.06194	0.00430	0.00775	0.00014	0.00241	0.00011	1.26	0.01	550	122	61	4	49.7	0.9	49	2
12LZ23-1-07	0.04249	0.00297	0.04531	0.00319	0.00789	0.00016	0.00235	0.00011	1.68	0.02	-150	118	45	3	51	1	48	2
12LZ23-1-08	0.04917	0.00283	0.05123	0.00270	0.00772	0.00011	0.00227	0.00008	1.18	0.01	156	94	51	3	49.6	0.7	46	2
~~12LZ23-1-09~~	~~0.06359~~	~~0.00657~~	~~0.06958~~	~~0.00724~~	~~0.00790~~	~~0.00022~~	~~0.00223~~	~~0.00015~~	~~1.71~~	~~0.02~~	~~728~~	~~174~~	~~68~~	~~7~~	~~51~~	~~1~~	~~45~~	~~3~~
12LZ23-1-10	0.05340	0.00288	0.05756	0.00308	0.00779	0.00010	0.00240	0.00007	1.34	0.01	346	98	57	3	50	0.6	48	1
12LZ23-1-11	0.05575	0.00388	0.05780	0.00392	0.00772	0.00014	0.00238	0.00010	1.26	0.01	442	120	57	4	49.6	0.9	48	2
12LZ23-1-12	0.05323	0.00382	0.05507	0.00391	0.00767	0.00013	0.00234	0.00010	1.23	0.01	339	130	54	4	49.3	0.9	47	2
12LZ23-1-13	0.04400	0.00231	0.04667	0.00240	0.00776	0.00012	0.00223	0.00007	1.32	0.01	-71	81	46	2	49.8	0.8	45	1

12LZ23-1-14	0.04633	0.00211	0.04972	0.00240	0.00768	0.00011	0.00227	0.00008	1.45	0.01	15	76	49	2	49.3	0.7	46	2
12LZ23-1-15	0.04942	0.00222	0.05302	0.00236	0.00777	0.00011	0.00223	0.00006	1.05	0.01	168	78	52	2	49.9	0.7	45	1
12LZ23-1-16	0.04168	0.00220	0.04426	0.00240	0.00769	0.00012	0.00208	0.00007	1.23	0.01	-194	95	44	2	49.4	0.8	42	1
12LZ23-1-17	0.04982	0.00344	0.05170	0.00325	0.00774	0.00013	0.00227	0.00007	1.07	0.01	187	113	51	3	49.7	0.8	46	1
12LZ23-1-18	0.04987	0.00292	0.05302	0.00300	0.00775	0.00011	0.00224	0.00007	1.03	0.01	189	104	52	3	49.8	0.7	45	1
13LZ06-1-01	0.04654	0.00363	0.05079	0.00387	0.00790	0.00012	0.00265	0.00009	1.64	0.02	26	140	50	4	50.8	0.7	54	2
13LZ06-1-02	0.05084	0.00230	0.05432	0.00244	0.00780	0.00009	0.00243	0.00006	0.99	0.01	234	83	54	2	50.1	0.6	49	1
13LZ06-1-03	0.04989	0.00344	0.05300	0.00353	0.00785	0.00014	0.00233	0.00009	1.32	0.01	190	119	52	3	50.4	0.9	47	2
13LZ06-1-04	0.04688	0.00239	0.05098	0.00271	0.00789	0.00010	0.00247	0.00007	0.99	0.01	43	92	50	3	50.7	0.6	50	1
~~13LZ06-1-05~~	~~0.04390~~	~~0.00398~~	~~0.05186~~	~~0.00467~~	~~0.00869~~	~~0.00012~~	~~0.00298~~	~~0.00009~~	~~1.18~~	~~0.01~~	~~-76~~	~~160~~	~~51~~	~~5~~	~~55.8~~	~~0.8~~	~~60~~	~~2~~
13LZ06-1-06	0.04805	0.00351	0.05201	0.00375	0.00787	0.00012	0.00264	0.00009	1.57	0.02	102	131	51	4	50.5	0.8	53	2
13LZ06-1-07	0.04455	0.00246	0.04848	0.00274	0.00782	0.00011	0.00251	0.00007	1.47	0.01	-42	94	48	3	50.2	0.7	51	2
13LZ06-1-08	0.04756	0.00316	0.05173	0.00335	0.00791	0.00012	0.00257	0.00009	1.37	0.01	77	115	51	3	50.8	0.7	52	2
13LZ06-1-09	0.04728	0.00388	0.05027	0.00405	0.00784	0.00012	0.00239	0.00007	0.86	0.01	63	148	50	4	50.3	0.8	48	1
13LZ06-1-10	0.04332	0.00297	0.04663	0.00318	0.00780	0.00010	0.00244	0.00006	0.66	0.01	-106	126	46	3	50.1	0.6	49	1
~~13LZ06-1-11~~	~~0.05666~~	~~0.00694~~	~~0.06053~~	~~0.00731~~	~~0.00775~~	~~0.00016~~	~~0.00241~~	~~0.00003~~	~~1.2~~	~~0.01~~	~~478~~	~~279~~	~~60~~	~~7~~	~~50~~	~~1~~	~~48.6~~	~~0.7~~
13LZ06-1-12	0.04852	0.00308	0.05205	0.00328	0.00787	0.00012	0.00257	0.00009	1.2	0.01	125	114	52	3	50.6	0.8	52	2
13LZ06-1-13	0.05220	0.00448	0.05443	0.00449	0.00786	0.00013	0.00244	0.00009	1.16	0.01	294	157	54	4	50.4	0.8	49	2
13LZ06-1-14	0.05118	0.00426	0.05570	0.00463	0.00791	0.00012	0.00242	0.00008	1.11	0.01	249	161	55	4	50.8	0.8	49	2
13LZ06-1-15	0.04663	0.00253	0.05141	0.00299	0.00790	0.00013	0.00275	0.00010	1.52	0.02	30	96	51	3	50.7	0.8	55	2
13LZ06-1-16	0.05049	0.00359	0.05479	0.00383	0.00783	0.00012	0.00258	0.00009	0.98	0.01	218	130	54	4	50.3	0.8	52	2
13LZ06-1-17	0.04805	0.00290	0.05176	0.00315	0.00786	0.00011	0.00256	0.00008	1.43	0.01	102	109	51	3	50.5	0.7	52	2
13LZ06-1-18	0.04763	0.00311	0.05081	0.00318	0.00788	0.00011	0.00251	0.00007	0.95	0.01	81	112	50	3	50.6	0.7	51	1

Magmatic record of India-Asia collision

Di-Cheng Zhu*, Qing Wang, Zhi-Dan Zhao, Sun-Lin Chung, Peter A. Cawood, Yaoling Niu, Sheng-Ao Liu, Fu-Yuan Wu, and Xuan-Xue Mo

Table S3 Zircon U-Pb age data of the 80-40 Ma intrusive rocks from the Gangdese Batholith (85–95°E) in southern Tibet

No.	Sample	Longitude	Latitude	Rock Type	SiO_2 (wt%)	Zircon U-Pb age (Ma)	± 2σ	References
1	06FW101	91.11	29.69	Monzogranite	72.29	64.7	1.1	Ji et al., 2012, Journal of Asian Earth Sciences 53, 82–95
2	06FW104	91.08	29.68	Monzogranite	71.71	64.4	0.9	Ji et al., 2012, Journal of Asian Earth Sciences 53, 82–95
3	06FW105	90.93	29.68	Granodiorite	67.62	55.2	1.5	Ji et al., 2012, Journal of Asian Earth Sciences 53, 82–95
4	06FW108	90.78	29.76	Diorite	62.25	56.8	0.7	Ji et al., 2012, Journal of Asian Earth Sciences 53, 82–95
5	06FW110	90.83	29.74	Monzogranite	73.45	54.3	0.9	Ji et al., 2012, Journal of Asian Earth Sciences 53, 82–95
6	06FW111	90.96	29.44	Monzogranite	66.49	50.6	0.7	Ji et al., 2012, Journal of Asian Earth Sciences 53, 82–95
7	06FW112	90.96	29.49	Granodiorite	60.23	53.4	1.0	Ji et al., 2012, Journal of Asian Earth Sciences 53, 82–95
8	06FW119	90.94	29.50	Granodiorite	63.91	51.2	0.7	Ji et al., 2012, Journal of Asian Earth Sciences 53, 82–95
9	06FW126	90.87	29.48	Diorite	56.62	55.3	1.0	Ji et al., 2012, Journal of Asian Earth Sciences 53, 82–95
10	06FW127	90.87	29.48	Granitic dike	77.16	49.5	0.6	Ji et al., 2012, Journal of Asian Earth Sciences 53, 82–95
11	06FW129	90.90	29.46	Diorite	57.43	52.9	0.7	Ji et al., 2012, Journal of Asian Earth Sciences 53, 82–95
12	06FW131	90.91	29.41	Granodiorite	69.90	44.0	0.8	Ji et al., 2012, Journal of Asian Earth Sciences 53, 82–95
13	06FW133	90.87	29.33	Monzonite	67.45	47.1	1.0	Ji et al., 2012, Journal of Asian Earth Sciences 53, 82–95
14	06FW134	90.88	29.36	Monzogranite	70.57	41.9	0.6	Ji et al., 2012, Journal of Asian Earth Sciences 53, 82–95
15	06FW139	90.72	29.38	Diorite	61.01	41.5	0.7	Ji et al., 2012, Journal of Asian Earth Sciences 53, 82–95
16	06FW140	90.72	29.47	Monzogranite	70.99	43.7	0.9	Ji et al., 2012, Journal of Asian Earth Sciences 53, 82–95
17	06FW146	90.72	29.40	Gabbro	52.88	56.9	1.4	Ji et al., 2012, Journal of Asian Earth Sciences 53, 82–95
18	06FW148	90.72	29.37	Monzogranite	71.69	51.3	0.6	Ji et al., 2012, Journal of Asian Earth Sciences 53, 82–95
19	06FW151	90.72	29.36	Diorite	56.09	55.5	1.2	Ji et al., 2012, Journal of Asian Earth Sciences 53, 82–95
20	06FW152-2	90.18	29.40	Diorite	53.49	57.3	0.9	Ji et al., 2012, Journal of Asian Earth Sciences 53, 82–95
21	06FW154	90.27	29.58	Monzogranite	75.43	51.3	0.7	Ji et al., 2012, Journal of Asian Earth Sciences 53, 82–95
22	06FW155	90.18	29.54	Bi-monzogranite	70.33	61.1	1.2	Ji et al., 2012, Journal of Asian Earth Sciences 53, 82–95
23	06FW156	90.27	29.50	Granodiorite	67.99	55.4	0.8	Ji et al., 2012, Journal of Asian Earth Sciences 53, 82–95
24	06FW162	89.62	29.54	Diorite	60.29	50.9	0.8	Ji et al., 2012, Journal of Asian Earth Sciences 53, 82–95
25	06FW174	90.10	29.35	Diorite	56.45	50.2	1.5	Ji et al., 2012, Journal of Asian Earth Sciences 53, 82–95
26	06FW175	90.07	29.35	Diorite	57.57	52.6	1.2	Ji et al., 2012, Journal of Asian Earth Sciences 53, 82–95

27	06FW176	90.25	29.33	Diorite	54.48	53.6	1.0	Ji et al., 2012, Journal of Asian Earth Sciences 53, 82–95
28	08CQ02	85.74	29.63	Monzogranite	78.07	43.9	0.3	Zhu et al., 2011, Earth and Planetary Science Letters 301, 241–255
29	08CQ03	85.76	29.78	Monzogranite	67.92	51.9	0.4	Zhu et al., 2011, Earth and Planetary Science Letters 301, 241–255
30	08CQ09	85.74	29.90	Monzogranite	68.52	50.0	0.4	Zhu et al., 2011, Earth and Planetary Science Letters 301, 241–255
31	08DX01	90.57	30.01	Diorite	55.57	69.8	0.7	Zhu et al., unpublished data
32	08FW50	91.18	29.64	Granodiorite	68.13	67.1	4.1	Ji, W.Q., 2010, Ph.D thesis, p. 1–146
33	08FW54	91.99	29.73	Diorite	54.83	65.6	1.4	Ji, W.Q., 2010, Ph.D thesis, p. 1–146
34	08FW56	92.20	29.71	Monzogranite	65.58	70.5	2.1	Ji, W.Q., 2010, Ph.D thesis, p. 1–146
35	08FW60	93.78	29.81	Monzogranite	77.24	47.6	2.5	Ji, W.Q., 2010, Ph.D thesis, p. 1–146
36	08FW61	94.01	29.76	Bi granite	75.98	53.8	1.4	Ji, W.Q., 2010, Ph.D thesis, p. 1–146
37	08FW62	94.29	29.74	Granodiorite	74.05	72.7	3.8	Ji, W.Q., 2010, Ph.D thesis, p. 1–146
38	08FW63	94.19	29.75	Granodiorite	73.05	53.0	3.8	Ji, W.Q., 2010, Ph.D thesis, p. 1–146
39	08FW66	92.04	29.72	Syenogranite	76.03	70.1	2.7	Ji, W.Q., 2010, Ph.D thesis, p. 1–146
40	09FW10	93.31	29.62	Gneissic granodiorite	65.52	66.2	1.1	Ji, W.Q., 2010, Ph.D thesis, p. 1–146
41	09FW30	93.20	29.15	Two mica monzogranite	72.25	76.7	1.6	Ji, W.Q., 2010, Ph.D thesis, p. 1–146
42	09FW33	93.18	29.12	Two mica monzogranite	79.70	79.7	1.3	Ji, W.Q., 2010, Ph.D thesis, p. 1–146
43	09FW41	92.74	29.42	Monzogranite	70.44	56.1	1.1	Ji, W.Q., 2010, Ph.D thesis, p. 1–146
44	09FW42	92.75	29.41	Granitic gneiss	69.74	50.7	1.1	Ji, W.Q., 2010, Ph.D thesis, p. 1–146
45	09FW43	92.75	29.41	Monzogranite	70.66	42.0	0.7	Ji, W.Q., 2010, Ph.D thesis, p. 1–146
46	09FW50	92.70	29.24	Monzogranite	67.85	50.2	0.9	Ji, W.Q., 2010, Ph.D thesis, p. 1–146
47	10CK-02	86.14	30.37	Granodiorite	68.00	65.2	0.5	Wang et al., 2012, Acta Petrologica Sinica, 28(5), 1647–1662
48	11LS01-1	90.94	29.50	Gabbro	50.13	47.3	0.4	Zhu et al., unpublished data
49	11LS06-1	90.71	29.36	Gabbro	54.35	46.2	1.5	Zhu et al., unpublished data
50	12AM04-2	86.32	29.63	Gabbro	52.03	44.8	0.2	Zhu et al., unpublished data
51	12AM06-2	86.32	29.58	Monzogranite	73.02	51.2	0.6	Zhu et al., unpublished data
52	12CZ15-1	86.75	29.75	Granodiorite	79.14	60.8	0.6	Zhu et al., unpublished data
53	12DJ08-1	87.79	29.44	Gabbro	66.09	51.8	0.5	Zhu et al., unpublished data
54	12DJ12-1	87.87	29.43	Monzogranite	66.72	44.3	0.4	Zhu et al., unpublished data
55	12LY00-1	89.24	29.72	Granodiorite	77.02	51.0	0.5	Zhu et al., unpublished data
56	12PC30-1	87.99	29.36	Granodiorite		52.3	0.6	Zhu et al., unpublished data
57	13WBD	89.55	29.29	Diabase dyke		49.8	1.3	Wang et al., 2013, Personal communication
58	D093TW	92.72	29.63	Granodiorite	68.90	70.4	2.2	Chen et al., 2010, Journal of Mineral and Petrology 30(1), 83–92
59	ET021D	91.63	29.69	Monzogranite	71.60	64.6	2.5	Wen, D.R., 2007, Ph.D thesis, National Taiwan University, p. 1–120
60	GR-20	93.42	29.08	Granite		68.0	4.0	Quidelleur et al., 1997, Journal of Geophysical Research 102(B2), 2659–2679

61	LS2	91.00	29.89	Granite	69.59	51.0	1.2	Huang et al., 2010. Acta Petrologica Sinica 26, 10, 3131–3142
62	LS5	91.24	29.69	Dioritie	56.09	61.0	2.9	Huang et al., 2010. Acta Petrologica Sinica 26, 10, 3131–3142
63	LZ09-2	94.47	29.55	Granite	75.22	51.4	1.4	Ji, W.Q., 2010, Ph.D thesis, p. 1–146
64	LZ11-1	94.46	29.46	Granodiorite	73.11	44.3	0.8	Ji, W.Q., 2010, Ph.D thesis, p. 1–146
65	LZ15-5	94.35	29.63	Dioritic enclave	55.22	64.0	1.2	Ji, W.Q., 2010, Ph.D thesis, p. 1–146
66	LZ16-2	94.37	29.60	Granodiorite	61.56	67.4	1.4	Ji, W.Q., 2010, Ph.D thesis, p. 1–146
67	LZ17-1	94.43	29.46	Two-mica monzogranite	73.23	51.7	1.0	Ji, W.Q., 2010, Ph.D thesis, p. 1–146
68	ML06-1	93.37	29.05	Granodiorite	70.58	79.3	0.4	Zhu et al., 2011, Earth and Planetary Science Letters 301, 241–255
69	ML07-1	93.52	29.17	Granodiorite	69.88	73.5	1.2	Ji, W.Q., 2010, Ph.D thesis, p. 1–146
70	ML08-1	93.47	29.15	Bt monzogranite	74.08	73.0	1.7	Ji, W.Q., 2010, Ph.D thesis, p. 1–146
71	ML09-1	93.38	29.06	Bt monzogranite	72.48	78.0	1.3	Ji, W.Q., 2010, Ph.D thesis, p. 1–146
72	ML11-1	93.12	29.11	Two-mica monzogranite	72.15	75.0	1.3	Ji, W.Q., 2010, Ph.D thesis, p. 1–146
73	ML18-4	93.68	29.15	Diorite	54.80	38.5	0.3	Guan et al., 2012, Gondwana Research 21, 88–99
74	ML19-1	93.75	29.13	Granodiorite	70.68	78.1	0.6	Guan et al., 2010, Acta Petrologica Sinica 26, 2165–2179
75	ML25-1	94.43	29.46	Syenogranite	76.11	56.7	1.0	Zhu et al., unpublished data
76	ML26-1	94.35	29.63	Granodiorite	66.17	64.8	0.5	Zhu et al., unpublished data
77	ML28-1	94.24	29.76	Bi monzogranite	70.23	53.4	0.4	Zhu et al., unpublished data
78	ML30-1	93.78	29.81	Granite	76.60	42.0	0.4	Zhu et al., unpublished data
79	ML37-1	93.30	29.60	Bi monzogranite	70.96	70.1	0.4	Zhu et al., unpublished data
80	ML38-2	93.30	29.60	Gabbroic enclave	48.23	69.5	0.7	Zhu et al., unpublished data
81	NB-159	94.70	29.75	Diorite	58.61	66.0	1.9	Booth et al., 2004, American Journal of Science 304, 889–929
82	NM01	89.08	29.54	Diorite	60.53	44.3	1.1	Cong et al., 2012, Journal of Jilin University 42 (6), 1783–1795
83	NM02	89.10	29.49	Granodiorite	65.28	45.5	1.1	Cong et al., 2012, Journal of Jilin University 42 (6), 1783–1795
84	NML03-1	89.06	29.62	Diorite	58.03	62.4	0.3	Zhu et al., 2011, Earth and Planetary Science Letters 301, 241–255
85	PD2-12	92.24	29.62	Syenogranite	77.26	66.6	0.7	Liu et al., 2012, Mineral Deposits 31, 4, 727–744
86	RB12	89.69	29.36	Diorite	54.73	45.0	1.6	Huang et al., 2010. Acta Petrologica Sinica 26, 10, 3131–3142
87	SR01-1	92.10	29.29	Granodiorite	62.52	45.4	0.7	Ji et al., 2012, Journal of Asian Earth Sciences 53, 82–95
88	SR02-1	92.12	29.29	Granite	72.05	37.7	0.7	Ji et al., 2012, Journal of Asian Earth Sciences 53, 82–95
89	SR03-1	92.22	29.27	Granodiorite	71.48	59.8	0.9	Ji et al., 2012, Journal of Asian Earth Sciences 53, 82–95
90	SR04-1	92.24	29.32	Granodiorite	61.81	42.1	0.7	Ji et al., 2012, Journal of Asian Earth Sciences 53, 82–95
91	ST043A	88.23	29.45	Granodiorite	63.60	50.4	1.2	Wen et al., 2008, Chemical Geology 252, 191–201
92	ST104A	91.81	29.27	Granodiorite	65.58	60.1	1.4	Chu et al., 2006, Geology 34, 745–748
93	ST147A	90.18	29.40	Diorite	53.87	50.6	0.7	Wen et al., 2008, Chemical Geology 252, 191–201
94	ST152A	90.69	29.33	Gabbro	49.75	52.7	1.4	Wen et al., 2008, Chemical Geology 252, 191–201

95	SWYT-1	91.69	29.87	Monzogranite		61.0	1.9	Luo et al., 2011, Mineral Deposits 30(2), 266–278
96	T024	93.75	29.14	Tonalite	69.53	80.4	1.1	Wen, D.R., 2007, Ph.D thesis, National Taiwan University, p. 1–120
97	T044E	89.08	29.49	Gabbro	52.10	48.0	1.2	Wen et al., 2008, Chemical Geology 252, 191–201
98	T076	91.22	29.67	Granodiorite	69.90	59.3	1.6	Wen et al., 2008, Chemical Geology 252, 191–201
99	T10-123-2	94.41	29.61	Two-mica granite	75.57	61.9	0.4	Zhang et al., 2013, Journal of Petrology 54, 12, 2547–2580
100	T10-74-2	94.71	29.64	Grt-Bt-Amp gneiss	55.24	61.3	1.2	Zhang et al., 2013, Journal of Petrology 54, 12, 2547–2580
101	T10-94-10	93.89	29.21	Gabbro	51.75	77.3	2.5	Zhang et al., 2014, Geochimica et Cosmochimica Acta, In press
102	T10-95-2	93.91	29.19	Pegimatite		68.2	1.2	Zhang et al., 2014, Geochimica et Cosmochimica Acta, In press
103	T308	90.93	29.48	Monzogranite	64.95	50.7	0.5	Xu, W.C., 2010. Ph.D thesis, China University of Geosciences (Wuhan), p. 1–172
104	T315	90.87	29.38	Granodiorite	59.77	40.5	0.5	Xu, W.C., 2010. Ph.D thesis, China University of Geosciences (Wuhan), p. 1–172
105	T316	90.87	29.38	Qtz diorite	61.49	47.1	0.7	Xu, W.C., 2010. Ph.D thesis, China University of Geosciences (Wuhan), p. 1–172
106	T364	90.27	29.51	Monzogranite	69.97	46.7	0.7	Xu, W.C., 2010. Ph.D thesis, China University of Geosciences (Wuhan), p. 1–172
107	T377	90.06	29.51	Granodiorite	66.43	54.1	1.3	Xu, W.C., 2010. Ph.D thesis, China University of Geosciences (Wuhan), p. 1–172
108	T390	90.12	29.35	Qtz diorite	61.02	48.5	0.8	Xu, W.C., 2010. Ph.D thesis, China University of Geosciences (Wuhan), p. 1–172
109	T392	88.37	29.95	Monzogranite	70.66	47.9	1.1	Xu, W.C., 2010. Ph.D thesis, China University of Geosciences (Wuhan), p. 1–172
110	T405	88.32	29.56	Monzogranite	68.88	49.0	1.0	Xu, W.C., 2010. Ph.D thesis, China University of Geosciences (Wuhan), p. 1–172
111	T420	87.45	29.38	Granodiorite	63.24	48.1	0.6	Xu, W.C., 2010. Ph.D thesis, China University of Geosciences (Wuhan), p. 1–172
112	T426	86.21	29.6	Monzogranite	67.72	50.5	0.8	Xu, W.C., 2010. Ph.D thesis, China University of Geosciences (Wuhan), p. 1–172
113	T439	89.10	29.46	Granodiorite	65.92	49.4	1.0	Xu, W.C., 2010. Ph.D thesis, China University of Geosciences (Wuhan), p. 1–172
114	T445	89.06	29.62	Monzogranite	74.36	40.7	0.5	Xu, W.C., 2010. Ph.D thesis, China University of Geosciences (Wuhan), p. 1–172
115	T446	89.06	29.63	Granodiorite	64.67	50.9	1.1	Xu, W.C., 2010. Ph.D thesis, China University of Geosciences (Wuhan), p. 1–172
116	T478	90.93	29.67	Monzogranite	67.58	46.4	0.8	Xu, W.C., 2010. Ph.D thesis, China University of Geosciences (Wuhan), p. 1–172
117	T479	90.60	30.04	Two-mica granite	71.22	49.6	0.6	Xu, W.C., 2010. Ph.D thesis, China University of Geosciences (Wuhan), p. 1–172
118	T490	91.98	29.73	Monzogranite	71.45	65.3	1.2	Xu, W.C., 2010. Ph.D thesis, China University of Geosciences (Wuhan), p. 1–172
119	T497	92.15	29.71	Granodiorite	64.12	67.7	1.2	Xu, W.C., 2010. Ph.D thesis, China University of Geosciences (Wuhan), p. 1–172
120	T529	94.43	29.45	Granitic gneiss	73.93	49.1	0.4	Xu, W.C., 2010. Ph.D thesis, China University of Geosciences (Wuhan), p. 1–172
121	T556	92.20	29.30	Monzogranite	69.71	59.7	0.6	Xu, W.C., 2010. Ph.D thesis, China University of Geosciences (Wuhan), p. 1–172
122	T560	93.18	29.26	Granite	70.00	60.9	0.9	Xu, W.C., 2010. Ph.D thesis, China University of Geosciences (Wuhan), p. 1–172
123	T670	94.44	29.55	Granitic gneiss	71.72	80.5	0.7	Guo et al., 2011, Lithos 127, 54–67
124	T8-15-2	94.01	29.30	Granodiorite	70.46	63.0	0.6	Zhang et al., 2013, Journal of Petrology 54, 12, 2547–2580
125	T9-33-5	94.44	29.55	Ep granodiorite	68.42	64.8	1.3	Zhang et al., 2013, Journal of Petrology 54, 12, 2547–2580
126	T9-33-7	94.44	29.55	Grt two-mica granodiorite	69.31	54.9	0.8	Zhang et al., 2013, Journal of Petrology 54, 12, 2547–2580
127	T9-62-1	94.45	29.43	Grt-Bt gneiss	64.27	63.6	1.8	Zhang et al., 2013, Journal of Petrology 54, 12, 2547–2580

Magmatic record of India-Asia collision

Di-Cheng Zhu*, Qing Wang, Zhi-Dan Zhao, Sun-Lin Chung, Peter A. Cawood, Yaoling Niu, Sheng-Ao Liu, Fu-Yuan Wu, and Xuan-Xue Mo

Table S4 Geochemical data of the Linzizong volcanic rocks in Linzhou Basin*

Sample	Rock type	Latitude	Longitude	Strata		Age	[SiO_2]	[TiO_2]	[Al_2O_3]	[Fe_2O_3]	[MnO]	[MgO]	[CaO]	[Na_2O]	[K_2O]	[P_2O_5]	LOI	Zr/Y	Zr (ppm)	Zircon saturation temperature (°C)	References
12LZ02-1	Andesite	29.95	91.20	Dianzhong	E1d	60.2	63.15	0.69	16.89	5.79	0.11	2.16	5.51	2.97	2.54	0.19	4.26		122	739	This study
12LZ03-1	Andesite	29.95	91.20	Dianzhong	E1d	60.2	63.45	0.67	16.67	5.56	0.12	2.09	5.69	3.16	2.40	0.19	3.87		122	734	This study
12LZ04-1	Andesite	29.95	91.20	Dianzhong	E1d	60.2	64.12	0.66	16.81	5.22	0.13	1.57	5.44	3.28	2.60	0.19	4.30		123	738	This study
12LZ06-1	Dacite	29.95	91.20	Dianzhong	E1d	58.5	66.01	0.69	16.46	5.85	0.06	1.77	4.62	2.95	1.42	0.19	2.59		103	747	This study
12LZ07-1	Andesite	29.95	91.20	Dianzhong	E1d	58.5	63.23	0.69	17.00	5.68	0.10	1.98	5.16	3.38	2.59	0.19	2.58		126	741	This study
12LZ08-1	Andesite	29.95	91.20	Dianzhong	E1d	58.5	60.21	0.82	17.51	7.14	0.10	1.93	6.35	3.32	2.41	0.22	4.73		133	731	This study
12LZ09-1	Andesite	29.95	91.20	Dianzhong	E1d	58.5	59.86	0.82	17.52	7.36	0.12	2.71	6.14	3.09	2.17	0.22	3.92		135	739	This study
12LZ10-1	Andesite	29.96	91.21	Dianzhong	E1d	58.5	60.58	0.80	17.41	7.17	0.15	2.77	5.40	3.35	2.13	0.22	3.38		138	747	This study
12LZ11-1	Andesite	29.96	91.21	Dianzhong	E1d	58.5	63.52	0.71	17.11	6.01	0.14	2.03	5.02	3.75	1.52	0.19	6.82		119	743	This study
12LZ12-1	Andesite	29.97	91.18	Dianzhong	E1d	58.5	57.41	0.90	18.45	8.39	0.12	1.64	6.26	3.33	3.26	0.24	6.30		105	709	This study
13LZ01-1	Andesite	29.95	91.20	Dianzhong	E1d	60.2	60.21	0.75	17.99	5.64	0.22	2.55	5.52	6.10	0.81	0.20	6.00		126	718	This study
13LZ02-1	Andesite	29.95	91.21	Dianzhong	E1d	60.2	56.78	0.87	19.42	7.14	0.27	2.55	8.50	3.06	1.13	0.27	4.66		122	717	This study
13LZ08-1	Andesite	29.98	91.19	Dianzhong	E1d	58.3	54.41	1.02	19.38	9.28	0.17	3.73	6.10	5.34	0.35	0.23	7.06				This study
D-2	Andesite	29.95	91.13	Dianzhong	E1d	58.5	60.02	0.71	16.25	8.64	0.15	3.71	3.72	4.17	2.42	0.19	3.62		136	746	Mo et al., 2008, Chemical Geology
D-15	Andesite	29.96	91.19	Dianzhong	E1d	58.5	59.04	0.69	16.19	9.89	0.15	2.57	6.60	2.78	1.91	0.17	4.22		118	717	Mo et al., 2008, Chemical Geology
BD-145	Andesite	29.95	91.20	Dianzhong	E1d	58.5	62.32	0.63	16.07	7.78	0.11	1.79	5.69	3.07	2.38	0.17	3.48		129	734	Mo et al., 2008, Chemical Geology
BD-151	Andesite	29.97	91.21	Dianzhong	E1d	58.5	58.38	0.65	18.33	8.49	0.11	1.94	8.05	2.65	1.22	0.18	4.56		156	742	Mo et al., 2008, Chemical Geology
BD-160	Andesite	29.96	91.18	Dianzhong	E1d	58.5	60.20	0.73	17.53	7.88	0.10	1.81	3.21	5.77	2.55	0.23	3.07		134	740	Mo et al., 2008, Chemical Geology
T240B	Andesite	29.91	91.07	Dianzhong	E1d	60.2	61.07	0.86	17.89	6.43	0.11	2.54	5.69	1.77	3.45	0.20	1.93		199	788	Lee et al., 2012, JAES
T239	Andesite	29.91	91.08	Dianzhong	E1d	60.2	59.49	0.94	18.35	6.81	0.10	2.97	7.15	2.44	1.55	0.20	3.16		188	771	Lee et al., 2012, JAES
T238B	Andesite	29.96	91.15	Dianzhong	E1d	60.2	55.77	0.95	19.02	8.54	0.11	3.36	8.95	2.82	0.26	0.22	5.07		161	737	Lee et al., 2012, JAES
T233B	Andesite	29.95	91.20	Dianzhong	E1d	60.2	61.41	0.77	17.55	6.39	0.14	2.54	4.17	4.38	2.47	0.18	2.50		126	742	Lee et al., 2012, JAES
BD-55	Rhyolitic tuff	29.98	91.17	Nianbo	E2n	54.5	76.32	0.12	12.15	1.91	0.09	0.44	2.42	2.49	4.02	0.03	3.09		114	753	Mo et al., 2008, Chemical Geology
BD-65	Basalt	29.98	91.17	Nianbo	E2n	52.7	51.11	0.95	17.57	11.86	0.17	3.36	8.92	2.94	2.67	0.45	5.03	3.8			Mo et al., 2008, Chemical Geology
BD-77	Rhyolitic tuff	29.98	91.14	Nianbo	E2n	52.6	70.22	0.25	18.42	0.90	0.01	0.27	0.29	4.22	5.35	0.06	1.68		234	843	Mo et al., 2008, Chemical Geology
LZ991	Rhyolitic tuff	29.97	91.13	Nianbo	E2n	55.4	76.16	0.13	14.72	2.18	0.02	1.08	0.98	0.57	4.12	0.03	3.54		113	811	Mo et al., 2008, Chemical Geology
LZ9921	Rhyolitic tuff	29.98	91.19	Nianbo	E2n	54.9	77.32	0.13	12.45	2.20	0.03	0.47	2.36	2.39	2.61	0.03	3.68		95.6	757	Mo et al., 2008, Chemical Geology
L060	Rhyolitic tuff	29.98	91.19	Nianbo	E2n	54.9	79.44	0.12	11.68	1.53	0.04	0.21	1.64	3.54	1.76	0.03	2.12		112	769	Mo et al., 2008, Chemical Geology
12LZ27-1	Rhyolitic tuff	29.98	91.15	Nianbo	E2n	52.6	73.31	0.63	17.92	1.70	0.01	0.48	0.22	0.59	5.02	0.12	3.34		380	946	This study
12LZ28-1	Basalt	29.97	91.15	Nianbo	E2n	52.9	50.77	0.98	18.95	9.23	0.20	3.27	7.36	4.17	4.56	0.50	6.36	5.5			This study
12LZ29-1	Rhyolite	29.97	91.15	Nianbo	E2n	55.4	76.05	0.37	13.71	1.40	0.03	0.90	0.65	5.35	1.44	0.09	2.16		165	804	This study
13LZ10-1	Basalt	29.98	91.19	Nianbo	E2n	54.5	48.16	1.14	17.35	10.55	0.54	5.16	10.31	3.62	2.79	0.38	8.17	3.7			This study
13LZ11-1	Basalt	29.98	91.19	Nianbo	E2n	54.5	51.85	1.09	18.24	10.31	0.13	5.30	7.41	3.95	1.27	0.48	8.63	5.3			This study
13LZ12-1	Andesite	29.98	91.19	Nianbo	E2n	54.5	56.00	0.88	15.79	9.90	0.17	5.60	5.55	3.96	1.79	0.35	5.35		114	702	This study
13LZ14-1	Basaltic andesite	29.98	91.19	Nianbo	E2n	54.5	53.36	0.87	17.98	9.49	0.17	2.40	9.24	3.30	2.72	0.48	4.84	5.9			This study
13LZ15-1	Basaltic andesite	29.98	91.19	Nianbo	E2n	54.5	54.50	0.87	17.58	8.85	0.16	3.38	8.53	3.15	2.53	0.44	3.65				This study
13LZ16-1	Andesite	29.98	91.19	Nianbo	E2n	52.7	57.01	0.76	18.43	7.57	0.15	3.01	6.58	3.40	2.66	0.43	6.93		157	737	This study
T235B	Rhyolitic tuff	29.98	91.17	Nianbo	E2n	54.9	78.21	0.12	12.89	1.10	0.04	0.33	1.58	2.82	2.90	0.01	2.67		106	771	Lee et al., 2012, JAES
T235A	Rhyolitic tuff	29.98	91.17	Nianbo	E2n	54.9	78.24	0.12	13.24	1.34	0.02	0.39	0.70	2.71	3.23	0.01	2.23		141	810	Lee et al., 2012, JAES
T234C	Dacite	29.98	91.18	Nianbo	E2n	54.5	68.36	0.57	15.12	5.20	0.07	1.13	2.97	4.31	1.99	0.27	2.35		174	786	Lee et al., 2012, JAES
T234B	Basaltic andesite	29.98	91.18	Nianbo	E2n	54.5	53.07	0.88	18.54	9.39	0.16	3.20	8.78	3.02	2.50	0.44	3.91	6.2			Lee et al., 2012, JAES
T234A	Andesite	29.98	91.18	Nianbo	E2n	54.5	56.72	0.71	18.26	7.53	0.15	2.98	6.78	3.25	3.28	0.37	2.89		168	735	Lee et al., 2012, JAES

12LZ16-1	Basalt	29.98	91.19	Pa'na	E2p	52.7	52.80	0.91	18.74	9.52	0.13	2.93	8.66	3.34	2.49	0.49	4.66	6.7			This study
12LZ17-1	Basalt	29.98	91.19	Pa'na	E2p	52.7	52.73	0.89	18.20	9.33	0.14	3.82	8.54	3.41	2.46	0.48	2.93	6.0			This study
12LZ18-1	Rhyolite	29.98	91.19	Pa'na	E2p	52.7	70.65	0.47	14.46	3.74	0.15	0.98	2.14	3.14	4.15	0.12	4.05		152	781	This study
12LZ23-1	Rhyolitic ignimbrite	29.99	91.14	Pa'na	E2p	52.2	75.45	0.21	14.41	1.22	0.05	0.11	0.22	2.95	5.32	0.07	1.29		136	796	This study
12LZ24-1	Rhyolitic ignimbrite	29.99	91.14	Pa'na	E2p	52.2	74.74	0.19	14.23	1.23	0.07	0.25	0.37	3.31	5.55	0.06	0.89		135	785	This study
12LZ25-1	Rhyolite	29.99	91.14	Pa'na	E2p	52.2	71.11	0.35	15.32	2.27	0.05	0.33	1.49	3.11	5.86	0.12	2.50		270	835	This study
12LZ26-1	Rhyolitic ignimbrite	29.98	91.14	Pa'na	E2p	52.2	77.52	0.34	15.72	0.34	0.01	0.39	0.14	0.31	5.17	0.06	2.74		252	900	This study
13LZ06-1	Rhyolite	30.00	91.15	Pa'na	E2p	52.2	71.34	0.31	14.07	2.62	0.11	0.77	2.60	2.89	5.18	0.09	3.08		140	761	This study
13LZ07-1	Rhyolitic ignimbrite	29.99	91.14	Pa'na	E2p	52.2	76.15	0.19	13.49	1.34	0.02	0.16	0.21	2.82	5.57	0.05	0.89		119	780	This study
LZ9917	Rhyolitic ignimbrite	30.01	91.15	Pa'na	E2p	52.5	76.65	0.30	11.37	3.06	0.03	0.49	0.60	1.73	5.63	0.14	1.77		113	771	Mo et al., 2008, Chemical Geology
LZ9916	Rhyolitic ignimbrite	30.01	91.15	Pa'na	E2p	52.5	80.26	0.31	9.83	2.76	0.02	0.23	0.39	2.32	3.71	0.16	1.12		101	767	Mo et al., 2008, Chemical Geology
BD-103	Rhyolitic ignimbrite	30.01	91.14	Pa'na	E2p	52.5	70.22	0.37	14.02	3.72	0.10	0.87	2.87	2.93	4.76	0.14	3.39		150	764	Mo et al., 2008, Chemical Geology
BD-106	Rhyolitic ignimbrite	30.01	91.14	Pa'na	E2p	52.5	69.35	0.37	14.68	4.05	0.11	0.73	3.21	2.54	4.80	0.16	3.93		176	781	Mo et al., 2008, Chemical Geology
P-1	Rhyolitic ignimbrite	29.99	91.15	Pa'na	E2p	52.2	74.41	0.17	13.39	1.76	0.08	0.28	1.07	3.35	5.46	0.04	0.80		142	774	Mo et al., 2008, Chemical Geology
11LZ01-1	Diabase dyke	29.97	91.15			52.9	51.35	0.94	18.94	9.20	0.15	3.44	8.07	3.15	4.28	0.48	6.15	5.2			Jia et al., 2013
11LZ01-2	Diabase dyke	29.97	91.15			52.9	50.65	0.93	18.25	9.41	0.23	3.21	8.76	4.76	3.34	0.46	7.08	4.6			Jia et al., 2013
11LZ02-1	Diabase dyke	29.97	91.15			52.9	51.92	0.95	18.58	9.55	0.15	4.75	7.41	2.87	3.36	0.47	5.58	5.1			Jia et al., 2013
11LZ02-2	Diabase dyke	29.97	91.15			52.9	49.52	0.95	18.55	10.32	0.28	3.57	9.09	5.46	1.91	0.36	7.77	4.3			Jia et al., 2013
11LZ02-3	Diabase dyke	29.97	91.15			52.9	52.41	0.84	18.91	9.52	0.17	3.34	6.95	3.63	3.77	0.46	5.72	4.9			Jia et al., 2013
11LZ03-1	Diabase dyke	29.97	91.15			52.9	51.16	0.85	19.21	9.81	0.13	3.09	7.05	4.84	3.40	0.47	6.71	4.9			Jia et al., 2013
11LZ03-2	Diabase dyke	29.97	91.15			52.9	51.91	0.87	19.18	9.51	0.17	3.91	7.13	3.69	3.17	0.46	5.99	4.9			Jia et al., 2013
11LZ04-1	Diabase dyke	29.97	91.15			52.9	51.97	0.93	19.22	9.43	0.14	3.89	5.85	4.81	3.36	0.40	5.83	4.8			Jia et al., 2013
2003T320	Diabase dyke					52.9	50.94	1.02	17.82	10.18	0.16	5.29	7.78	2.91	3.44	0.46	4.75	5.2			Yue et al., 2006
2003T324	Diabase dyke					52.9	47.29	0.90	17.21	11.86	0.20	5.42	10.32	3.70	2.61	0.49	0.86	4.1			Yue et al., 2006
2003T348	Diabase dyke					52.9	49.60	1.01	17.86	11.15	0.17	4.39	10.45	2.39	2.56	0.42	7.08	4.6			Yue et al., 2006
2003T350	Diabase dyke					52.9	48.62	1.10	17.32	11.59	0.18	5.51	10.16	2.56	2.45	0.50	6.90	5.1			Yue et al., 2006
2003T353	Diabase dyke					52.9	49.78	1.07	18.62	10.85	0.19	3.04	10.67	2.94	2.37	0.46	6.23	4.8			Yue et al., 2006
2003T363	Diabase dyke					52.9	50.40	1.21	17.32	10.29	0.12	5.23	9.64	3.03	2.31	0.45	5.08	5.3			Yue et al., 2006

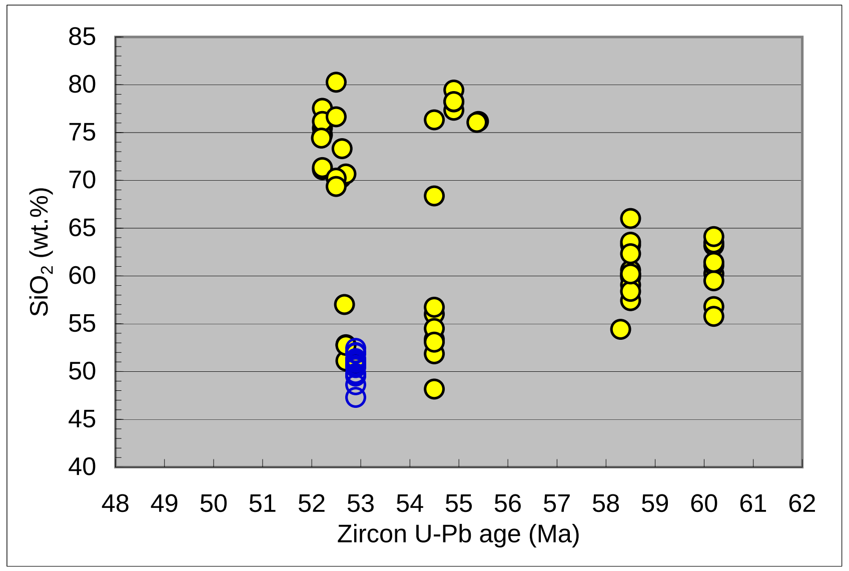

Fig. 3a Plot of SiO2 content versus age (Ma) for the Linzizong volcanic rocks in Linzhou Basin (see Table S4 for data)

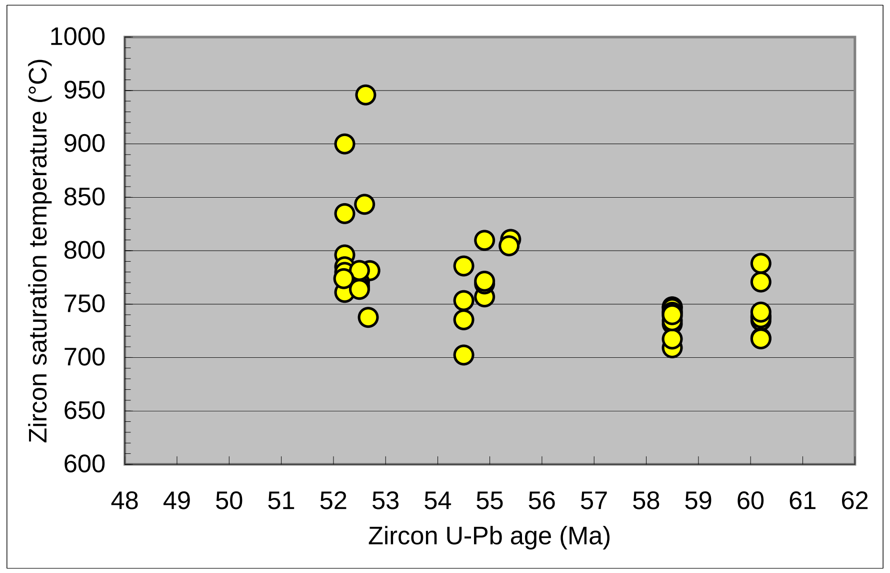

Fig. 3b Plot of zircon saturation temperature (°C) versus age (Ma) for the Linzizong volcanic rocks (see Table S4 for data)

See captions of the Figures 2a and 2b in the text for detrails

Note: 1) Age data used for this table are from Table 1 and Yue and Ding (2006); 2) Major element contents are recalculated on an anhydrous basis. Zircon saturation temperatures were calculated from whole-rock compositions with SiO_2 > 56 wt.% following the method of Watson and Harrison (1983)

Magmatic record of India-Asia collision

Di-Cheng Zhu*, Qing Wang, Zhi-Dan Zhao, Sun-Lin Chung, Peter A. Cawood, Yaoling Niu, Sheng-Ao Liu, Fu-Yuan Wu, and Xuan-Xue Mo

Table S5 SiO_2 contents of the 80-40 Ma intrusive rocks from the Gangdese Batholith (85–95°E) in southern Tibet

No.	Sample	Longitude	Latitude	Rock Type	Age (Ma)	SiO_2	References
1	06FW101	91.11	29.69	Monzogranite	64.7	72.29	Ji et al., 2012, Journal of Asian Earth Sciences 53, 82–95
2	06FW104	91.08	29.68	Monzogranite	64.4	71.71	Ji et al., 2012, Journal of Asian Earth Sciences 53, 82–95
3	06FW105	90.93	29.68	Monzogranite	55.2	67.62	Ji et al., 2012, Journal of Asian Earth Sciences 53, 82–95
4	06FW108	90.78	29.76	Granodiorite	56.8	62.25	Ji et al., 2012, Journal of Asian Earth Sciences 53, 82–95
5	06FW110	90.83	29.74	Monzogranite	54.3	73.45	Ji et al., 2012, Journal of Asian Earth Sciences 53, 82–95
6	06FW111	90.96	29.44	Monzogranite	50.6	66.49	Ji et al., 2012, Journal of Asian Earth Sciences 53, 82–95
7	06FW112	90.96	29.49	Granodiorite	53.4	60.23	Ji et al., 2012, Journal of Asian Earth Sciences 53, 82–95
8	06FW118	90.94	29.50	Monzogranite	51.0	73.27	Ji et al., 2012, Journal of Asian Earth Sciences 53, 82–95
9	06FW119	90.94	29.50	Granodiorite	51.2	63.91	Ji et al., 2012, Journal of Asian Earth Sciences 53, 82–95
10	06FW120	90.94	29.50	Dioritic enclave	50.3	51.94	Ji et al., 2012, Journal of Asian Earth Sciences 53, 82–95
11	06FW121	90.94	29.50	Graniticdike	51.1	75.29	Ji et al., 2012, Journal of Asian Earth Sciences 53, 82–95
12	06FW126	90.87	29.48	Granodiorite	55.3	56.62	Ji et al., 2012, Journal of Asian Earth Sciences 53, 82–95
13	06FW127	90.87	29.48	Graniticdike	49.5	77.16	Ji et al., 2012, Journal of Asian Earth Sciences 53, 82–95
14	06FW128	90.87	29.48	Doleriticdike	49.9	54.99	Ji et al., 2012, Journal of Asian Earth Sciences 53, 82–95
15	06FW129	90.90	29.46	Granodiorite	52.9	57.43	Ji et al., 2012, Journal of Asian Earth Sciences 53, 82–95
16	06FW131	90.91	29.41	Tonalitic gneiss	44.0	69.90	Ji et al., 2012, Journal of Asian Earth Sciences 53, 82–95
17	06FW133	90.87	29.33	Monzonite	47.1	67.45	Ji et al., 2012, Journal of Asian Earth Sciences 53, 82–95
18	06FW134	90.88	29.36	Monzogranite	41.9	70.57	Ji et al., 2012, Journal of Asian Earth Sciences 53, 82–95
19	06FW139	90.88	29.38	Monzonite	41.5	61.01	Ji et al., 2012, Journal of Asian Earth Sciences 53, 82–95
20	06FW140	90.72	29.47	Monzogranite	43.7	70.99	Ji et al., 2012, Journal of Asian Earth Sciences 53, 82–95
21	06FW146	90.72	29.40	Monzodiorite	56.9	52.88	Ji et al., 2012, Journal of Asian Earth Sciences 53, 82–95
22	06FW147	90.72	29.37	Granodiorite	51.5	61.42	Ji et al., 2012, Journal of Asian Earth Sciences 53, 82–95
23	06FW148	90.72	29.37	Syenogranitic dyke	51.3	71.69	Ji et al., 2012, Journal of Asian Earth Sciences 53, 82–95
24	06FW151	90.72	29.36	Diorite	55.5	56.09	Ji et al., 2012, Journal of Asian Earth Sciences 53, 82–95

25	06FW152-2	90.18	29.40	Diorite	57.3	53.49	Ji et al., 2012, Journal of Asian Earth Sciences 53, 82–95
26	06FW154	90.27	29.58	Syenogranite	51.3	75.43	Ji et al., 2012, Journal of Asian Earth Sciences 53, 82–95
27	06FW155	90.27	29.54	Monzodiorite	61.1	70.33	Ji et al., 2012, Journal of Asian Earth Sciences 53, 82–95
28	06FW156	90.27	29.50	Monzodiorite	55.4	67.99	Ji et al., 2012, Journal of Asian Earth Sciences 53, 82–95
29	06FW162	89.62	29.54	Granodiorite	48.2	60.29	Ji et al., 2012, Journal of Asian Earth Sciences 53, 82–95
30	06FW163	89.62	29.54	Monzogranite	50.9	71.58	Ji et al., 2012, Journal of Asian Earth Sciences 53, 82–95
31	06FW174	90.10	29.35	Diorite	50.2	56.45	Ji et al., 2012, Journal of Asian Earth Sciences 53, 82–95
32	06FW175	90.07	29.35	Diorite	52.6	57.57	Ji et al., 2012, Journal of Asian Earth Sciences 53, 82–95
33	06FW176	90.25	29.34	Diorite	53.6	54.48	Ji et al., 2012, Journal of Asian Earth Sciences 53, 82–95
34	08CQ01	85.74	29.63	Syenogranite	43.9	76.87	Wang and Zhu et al., 2014, To be submitted
35	08CQ02	85.74	29.63	Syenogranite	43.9	78.07	Zhu et al., 2011, Earth and Planetary Science Letters 301, 241–255
36	08CQ03	85.76	29.78	Monzogranite	51.9	67.92	Zhu et al., 2011, Earth and Planetary Science Letters 301, 241–255
37	08CQ09	85.74	29.90	Granodiorite porphyry	50.0	68.52	Zhu et al., 2011, Earth and Planetary Science Letters 301, 241–255
38	08CQ13	85.41	30.14	Diorite	51.5	54.04	Zhu et al., 2011, Earth and Planetary Science Letters 301, 241–255
39	08DX01	90.57	30.01	Diorite	69.8	55.57	Zhu et al., unpublished data
40	08FW50	91.18	29.64	Granodiorite	67.1	68.13	Ji, W.Q., 2010, Ph.D thesis, p. 1–146
41	08FW51	91.16	29.64	Granodiorite	64.5	66.35	Ji et al., 2012, Journal of Asian Earth Sciences 53, 82–95
42	08FW54	91.99	29.73	Diorite	65.6	54.83	Ji, W.Q., 2010, Ph.D thesis, p. 1–146
43	08FW56	92.20	29.71	Monzogranite	70.5	65.58	Ji, W.Q., 2010, Ph.D thesis, p. 1–146
44	08FW60	93.78	29.81	Monzogranite	47.6	77.24	Ji, W.Q., 2010, Ph.D thesis, p. 1–146
45	08FW61	94.01	29.76	Syenogranite	53.8	75.98	Ji, W.Q., 2010, Ph.D thesis, p. 1–146
46	08FW62	94.29	29.74	Monzogranite	72.7	74.05	Ji, W.Q., 2010, Ph.D thesis, p. 1–146
47	08FW63	94.19	29.75	Monzogranite	53.0	73.05	Ji, W.Q., 2010, Ph.D thesis, p. 1–146
48	08FW66	92.04	29.72	Syenogranite	70.1	76.03	Ji, W.Q., 2010, Ph.D thesis, p. 1–146
49	09FW08	93.31	29.62	Gabbro	66.0	47.34	Ji, W.Q., 2010, Ph.D thesis, p. 1–146
50	09FW09	93.31	29.62	Diorite	66.6	54.38	Ji, W.Q., 2010, Ph.D thesis, p. 1–146
51	09FW10	93.31	29.62	Gneissic granodiorite	66.2	65.52	Ji, W.Q., 2010, Ph.D thesis, p. 1–146
52	09FW30	93.20	29.15	Two-mica Monzogranite	76.7	72.25	Ji, W.Q., 2010, Ph.D thesis, p. 1–146
53	09FW33	93.18	29.12	Two-mica Monzogranite	79.7	72.76	Ji, W.Q., 2010, Ph.D thesis, p. 1–146
54	09FW41	92.74	29.42	Monzogranite	56.1	70.44	Ji et al., 2012, Journal of Asian Earth Sciences 53, 82–95

55	09FW42	92.75	29.41	Monzogranite	50.7	69.74	Ji et al., 2012, Journal of Asian Earth Sciences 53, 82–95
56	09FW43	92.75	29.41	Monzogranite	42.0	70.66	Ji et al., 2012, Journal of Asian Earth Sciences 53, 82–95
57	09FW50	92.70	29.24	Granodiorite	50.2	67.85	Ji et al., 2012, Journal of Asian Earth Sciences 53, 82–95
58	10CK-02	86.14	30.37	Granitic porphyry	65.2	68.00	Wang et al., 2012, Acta Petrologica Sinica, 28(5), 1647–1662
59	10CK-03	86.14	30.37	Granitic porphyry	65.2	76.89	Wang et al., 2012, Acta Petrologica Sinica, 28(5), 1647–1662
60	10CK-04	86.14	30.37	Granitic porphyry	65.2	75.49	Wang et al., 2012, Acta Petrologica Sinica, 28(5), 1647–1662
61	10CK-07	86.14	30.37	Granitic porphyry	64.4	75.66	Wang et al., 2012, Acta Petrologica Sinica, 28(5), 1647–1662
62	10CK-08	86.14	30.37	Granitic porphyry	64.4	76.18	Wang et al., 2012, Acta Petrologica Sinica, 28(5), 1647–1662
63	10CK-10	86.14	30.37	Granitic porphyry	64.4	75.98	Wang et al., 2012, Acta Petrologica Sinica, 28(5), 1647–1662
64	10CK-11	86.14	30.37	Granitic porphyry	64.4	77.01	Wang et al., 2012, Acta Petrologica Sinica, 28(5), 1647–1662
65	10CK-14	86.14	30.37	Granitic porphyry	64.4	68.86	Wang et al., 2012, Acta Petrologica Sinica, 28(5), 1647–1662
66	12DJC12-1	85.72	29.63	Syenogranite	42.6	69.79	Wang and Zhu et al., 2014, To be submitted
67	12DJC13-1	85.74	29.63	Syenogranite	42.6	68.66	Wang and Zhu et al., 2014, To be submitted
68	12DJC13-2	85.74	29.63	Syenogranite	42.7	71.49	Wang and Zhu et al., 2014, To be submitted
69	12DJC14-1	85.74	29.63	Syenogranite	43.6	71.59	Wang and Zhu et al., 2014, To be submitted
70	12DJC14-2	85.74	29.63	Monzogranite	43.6	71.62	Wang and Zhu et al., 2014, To be submitted
71	12DJC14-3	85.74	29.63	Syenogranite	43.6	75.59	Wang and Zhu et al., 2014, To be submitted
72	12DJC15-1	85.74	29.63	Monzogranite	43.6	76.85	Wang and Zhu et al., 2014, To be submitted
73	12DJC15-2	85.74	29.63	Syenogranite	43.6	70.08	Wang and Zhu et al., 2014, To be submitted
74	12DJC15-3	85.74	29.63	Syenogranite	43.6	74.72	Wang and Zhu et al., 2014, To be submitted
75	12DJC16-1	85.74	29.62	Syenogranite	43.6	77.01	Wang and Zhu et al., 2014, To be submitted
76	12DJC16-2	85.74	29.62	Monzogranite	43.6	77.25	Wang and Zhu et al., 2014, To be submitted
77	12DJC16-3	85.74	29.62	Monzogranite	43.9	76.33	Wang and Zhu et al., 2014, To be submitted
78	LS2	91.00	29.89	Granodiorite	51.0	69.59	Huang et al., 2010. Acta Petrologica Sinica 26, 10, 3131–3142
79	LS3	91.00	29.89	Dioritic enclave	61.0	54.14	Huang et al., 2010. Acta Petrologica Sinica 26, 10, 3131–3142
80	LS4	91.27	29.68	Dioritie	61.0	54.15	Huang et al., 2010. Acta Petrologica Sinica 26, 10, 3131–3142
81	LS5	91.24	29.69	Dioritie	61.0	56.09	Huang et al., 2010. Acta Petrologica Sinica 26, 10, 3131–3142
82	LZ09-2	94.47	29.55	Granitic gneiss	51.4	75.22	Ji, W.Q., 2010, Ph.D thesis, p. 1–146
83	LZ11-1	94.46	29.46	Gneissic granodiorite	44.3	73.11	Ji, W.Q., 2010, Ph.D thesis, p. 1–146
84	LZ13-1	94.38	29.64	Granodiorite	67.1	68.23	Ji, W.Q., 2010, Ph.D thesis, p. 1–146

85	LZ15-5	94.35	29.63	Dioritic enclave	64.0	55.22	Ji, W.Q., 2010, Ph.D thesis, p. 1–146
86	LZ16-2	94.37	29.60	Granodiorite	67.4	61.56	Ji, W.Q., 2010, Ph.D thesis, p. 1–146
87	LZ17-1	94.43	29.46	Two-mica monzogranite	51.7	73.23	Ji, W.Q., 2010, Ph.D thesis, p. 1–146
88	ML05-1	93.77	29.14	Monzogranite	78.4	70.93	Ji, W.Q., 2010, Ph.D thesis, p. 1–146
89	ML05-1	93.35	29.04	Granodiorite	79.3	67.17	Guan et al., 2010, Acta Petrologica Sinica 26, 2165–2179
90	ML06-1	93.37	29.05	Granodiorite	79.3	70.58	Zhu et al., 2011, Earth and Planetary Science Letters 301, 241–255
91	ML07-1	93.52	29.17	Granodiorite	73.5	69.88	Ji, W.Q., 2010, Ph.D thesis, p. 1–146
92	ML08-1	93.47	29.15	Monzogranite	73.0	74.08	Ji, W.Q., 2010, Ph.D thesis, p. 1–146
93	ML08-1	93.39	29.09	Monzogranite	79.3	70.67	Guan et al., 2010, Acta Petrologica Sinica 26, 2165–2179
94	ML09-1	93.38	29.06	Monzogranite	78.0	72.48	Ji, W.Q., 2010, Ph.D thesis, p. 1–146
95	ML11-1	93.12	29.11	Two-mica granite	75.0	72.15	Ji, W.Q., 2010, Ph.D thesis, p. 1–146
96	ML18-1	93.68	29.15	Gabbroic enclave	38.5	51.08	Guan et al., 2012, Gondwana Research 21, 88–99
97	ML18-10	93.68	29.15	Granodiorite	37.4	65.91	Guan et al., 2012, Gondwana Research 21, 88–99
98	ML18-2	93.68	29.15	Granodiorite	37.4	71.03	Guan et al., 2012, Gondwana Research 21, 88–99
99	ML18-3	93.68	29.15	Granodiorite	37.4	66.59	Guan et al., 2012, Gondwana Research 21, 88–99
100	ML18-4	93.68	29.15	Dioritic enclave	38.5	54.80	Guan et al., 2012, Gondwana Research 21, 88–99
101	ML18-5	93.68	29.15	Dioritic enclave	38.5	56.29	Guan et al., 2012, Gondwana Research 21, 88–99
102	ML18-6	93.68	29.15	Dioritic enclave	38.5	55.97	Guan et al., 2012, Gondwana Research 21, 88–99
103	ML18-7	93.68	29.15	Granodiorite	37.4	66.35	Guan et al., 2012, Gondwana Research 21, 88–99
104	ML18-8	93.68	29.15	Granodiorite	37.4	65.26	Guan et al., 2012, Gondwana Research 21, 88–99
105	ML18-9	93.68	29.15	Granodiorite	37.4	68.07	Guan et al., 2012, Gondwana Research 21, 88–99
106	ML19-1	93.75	29.13	Monzogranite	78.1	70.68	Guan et al., 2010, Acta Petrologica Sinica 26, 2165–2179
107	ML25-1	94.43	29.46	Monzogranite	56.7	76.11	Zhu et al., unpublished data
108	ML26-1	94.35	29.63	Granodiorite	64.8	66.17	Zhu et al., unpublished data
109	ML26-2	94.35	29.63	Monzogranite	64.8	73.82	Zhu et al., unpublished data
110	ML28-1	94.24	29.76	Monzogranite	54.5	70.23	Zhu et al., unpublished data
111	ML37-1	93.30	29.60	Monzogranite	70.1	70.96	Zhu et al., unpublished data
112	ML37-2	93.30	29.60	Dioritic enclave	69.8	55.62	Zhu et al., unpublished data
113	ML37-3	93.30	29.60	Dioritic enclave	70.1	55.79	Zhu et al., unpublished data
114	ML37-5	93.30	29.60	Gabbroic enclave	69.8	50.46	Zhu et al., unpublished data

115	ML38-2	93.30	29.60	Gabbroic enclave	69.5	48.23	Zhu et al., unpublished data
116	ML38-3	93.30	29.60	Gabbroic enclave	69.5	49.57	Zhu et al., unpublished data
117	ML38-6	93.30	29.60	Gabbroic enclave	69.5	47.97	Zhu et al., unpublished data
118	NML03-1	89.06	29.62	Diorite	62.4	58.03	Zhu et al., 2011, Earth and Planetary Science Letters 301, 241–255
119	RB10	89.69	29.36	Gabbro	45.0	50.84	Huang et al., 2010. Acta Petrologica Sinica 26, 10, 3131–3142
120	RB12	89.69	29.36	Diorite	45.0	54.73	Huang et al., 2010. Acta Petrologica Sinica 26, 10, 3131–3142
121	RB15	89.69	29.36	Gabbroic enclave	45.0	49.80	Huang et al., 2010. Acta Petrologica Sinica 26, 10, 3131–3142
122	SR01-1	92.10	29.29	Granodiorite	45.4	62.52	Ji et al., 2012, Journal of Asian Earth Sciences 53, 82–95
123	SR02-1	92.12	29.29	Granite	37.7	72.05	Ji et al., 2012, Journal of Asian Earth Sciences 53, 82–95
124	SR03-1	92.22	29.27	Granodiorite	59.8	71.48	Ji et al., 2012, Journal of Asian Earth Sciences 53, 82–95
125	SR04-1	92.24	29.32	Granodiorite	42.1	61.81	Ji et al., 2012, Journal of Asian Earth Sciences 53, 82–95
126	T024	93.75	29.14	Granodiorite	80.4	69.53	Wen et al., 2008, Lithos 105, 1–11
127	T026	93.44	29.12	Granodiorite	80.4	70.11	Wen et al., 2008, Lithos 105, 1–11
128	T10-113-8	94.44	29.55	Grt two-mica granite	63.3	72.21	Zhang et al., 2013, Journal of Petrology 54, 12, 2547–2580
129	T10-123-2	94.41	29.61	Two-mica granite	61.9	75.57	Zhang et al., 2013, Journal of Petrology 54, 12, 2547–2580
130	T10-72-2	94.79	29.96	Grt two-mica granodiorite	65.9	68.34	Zhang et al., 2013, Journal of Petrology 54, 12, 2547–2580
131	T215	93.41	29.10	Tonalite	80.4	68.28	Wen et al., 2008, Lithos 105, 1–11
132	T216A	93.61	29.17	Tonalite	80.4	67.89	Wen et al., 2008, Lithos 105, 1–11
133	T217	93.64	29.14	Tonalite	80.4	68.78	Wen et al., 2008, Lithos 105, 1–11
134	T218B	93.75	29.14	Tonalite	80.4	67.79	Wen et al., 2008, Lithos 105, 1–11
135	T300	90.95	29.54	Granodiorite	50.3	65.58	Xu, W.C., 2010. Ph.D thesis, China University of Geosciences (Wuhan), p. 1–172
136	T308	90.93	29.48	Granodiorite	50.7	64.95	Xu, W.C., 2010. Ph.D thesis, China University of Geosciences (Wuhan), p. 1–172
137	T315	90.87	29.38	Qtz diorite	40.5	59.77	Xu, W.C., 2010. Ph.D thesis, China University of Geosciences (Wuhan), p. 1–172
138	T316	90.87	29.38	Qtz diorite	47.1	61.49	Xu, W.C., 2010. Ph.D thesis, China University of Geosciences (Wuhan), p. 1–172
139	T318	90.87	29.38	Qtz diorite	47.1	60.91	Xu, W.C., 2010. Ph.D thesis, China University of Geosciences (Wuhan), p. 1–172
140	T319	90.73	29.35	Qtz diorite	47.1	63.72	Xu, W.C., 2010. Ph.D thesis, China University of Geosciences (Wuhan), p. 1–172
141	T364	90.27	29.51	Granodiorite	46.7	69.97	Xu, W.C., 2010. Ph.D thesis, China University of Geosciences (Wuhan), p. 1–172
142	T365	90.27	29.51	Granodiorite	46.7	66.80	Xu, W.C., 2010. Ph.D thesis, China University of Geosciences (Wuhan), p. 1–172
143	T366	90.27	29.51	Granodiorite	46.7	62.78	Xu, W.C., 2010. Ph.D thesis, China University of Geosciences (Wuhan), p. 1–172
144	T375	90.06	29.51	Qtz diorite	54.1	61.85	Xu, W.C., 2010. Ph.D thesis, China University of Geosciences (Wuhan), p. 1–172

145	T376	90.06	29.51	Granodiorite	54.1	66.31	Xu, W.C., 2010. Ph.D thesis, China University of Geosciences (Wuhan), p. 1–172
146	T377	90.06	29.51	Granodiorite	54.1	66.43	Xu, W.C., 2010. Ph.D thesis, China University of Geosciences (Wuhan), p. 1–172
147	T378	90.05	29.52	Granodiorite	54.1	67.46	Xu, W.C., 2010. Ph.D thesis, China University of Geosciences (Wuhan), p. 1–172
148	T390	90.12	29.35	Qtz diorite	48.5	61.02	Xu, W.C., 2010. Ph.D thesis, China University of Geosciences (Wuhan), p. 1–172
149	T391	90.02	29.34	Diorite	48.5	54.18	Xu, W.C., 2010. Ph.D thesis, China University of Geosciences (Wuhan), p. 1–172
150	T392	88.37	29.95	Monzogranite	47.9	70.66	Xu, W.C., 2010. Ph.D thesis, China University of Geosciences (Wuhan), p. 1–172
151	T393	88.37	29.95	Monzogranite	47.9	70.32	Xu, W.C., 2010. Ph.D thesis, China University of Geosciences (Wuhan), p. 1–172
152	T395	88.37	29.95	Monzogranite	47.9	71.35	Xu, W.C., 2010. Ph.D thesis, China University of Geosciences (Wuhan), p. 1–172
153	T396	88.39	29.87	Monzogranite	47.9	76.28	Xu, W.C., 2010. Ph.D thesis, China University of Geosciences (Wuhan), p. 1–172
154	T398	88.39	29.83	Monzogranite	47.9	75.32	Xu, W.C., 2010. Ph.D thesis, China University of Geosciences (Wuhan), p. 1–172
155	T405	88.32	29.56	Monzogranite	49.0	68.88	Xu, W.C., 2010. Ph.D thesis, China University of Geosciences (Wuhan), p. 1–172
156	T406	88.32	29.56	Monzogranite	49.0	66.31	Xu, W.C., 2010. Ph.D thesis, China University of Geosciences (Wuhan), p. 1–172
157	T410	88.32	29.51	Monzogranite	49.0	66.86	Xu, W.C., 2010. Ph.D thesis, China University of Geosciences (Wuhan), p. 1–172
158	T411	88.32	29.48	Monzogranite	49.0	69.13	Xu, W.C., 2010. Ph.D thesis, China University of Geosciences (Wuhan), p. 1–172
159	T420	87.45	29.38	Granodiorite	48.1	63.24	Xu, W.C., 2010. Ph.D thesis, China University of Geosciences (Wuhan), p. 1–172
160	T426	86.21	29.6	Monzogranite	50.5	67.72	Xu, W.C., 2010. Ph.D thesis, China University of Geosciences (Wuhan), p. 1–172
161	T427	86.21	29.58	Monzogranite	50.5	69.93	Xu, W.C., 2010. Ph.D thesis, China University of Geosciences (Wuhan), p. 1–172
162	T430	86.21	29.58	Monzogranite	48.2	68.12	Xu, W.C., 2010. Ph.D thesis, China University of Geosciences (Wuhan), p. 1–172
163	T432	86.22	29.57	Monzogranite	48.2	70.32	Xu, W.C., 2010. Ph.D thesis, China University of Geosciences (Wuhan), p. 1–172
164	T439	89.10	29.46	Granodiorite	49.4	65.92	Xu, W.C., 2010. Ph.D thesis, China University of Geosciences (Wuhan), p. 1–172
165	T440	89.10	29.47	Qtz diorite	49.4	60.28	Xu, W.C., 2010. Ph.D thesis, China University of Geosciences (Wuhan), p. 1–172
166	T442	89.09	29.53	Qtz diorite	49.4	61.65	Xu, W.C., 2010. Ph.D thesis, China University of Geosciences (Wuhan), p. 1–172
167	T443	89.08	29.55	Qtz diorite	49.4	61.10	Xu, W.C., 2010. Ph.D thesis, China University of Geosciences (Wuhan), p. 1–172
168	T445	89.06	29.62	Monzogranite	40.7	74.36	Xu, W.C., 2010. Ph.D thesis, China University of Geosciences (Wuhan), p. 1–172
169	T446	89.06	29.63	Granodiorite	50.9	64.67	Xu, W.C., 2010. Ph.D thesis, China University of Geosciences (Wuhan), p. 1–172
170	T447	89.06	29.63	Granodiorite	50.9	64.88	Xu, W.C., 2010. Ph.D thesis, China University of Geosciences (Wuhan), p. 1–172
171	T477	90.93	29.67	Granodiorite	49.9	68.54	Xu, W.C., 2010. Ph.D thesis, China University of Geosciences (Wuhan), p. 1–172
172	T478	90.93	29.67	Monzogranite	46.4	67.58	Xu, W.C., 2010. Ph.D thesis, China University of Geosciences (Wuhan), p. 1–172
173	T479	90.60	30.04	Monzogranite	49.6	71.22	Xu, W.C., 2010. Ph.D thesis, China University of Geosciences (Wuhan), p. 1–172
174	T490	91.98	29.73	Monzogranite	65.3	71.45	Xu, W.C., 2010. Ph.D thesis, China University of Geosciences (Wuhan), p. 1–172

175	T491	92.02	29.72	Monzogranite	65.3	72.81	Xu, W.C., 2010. Ph.D thesis, China University of Geosciences (Wuhan), p. 1–172
176	T493	92.02	29.72	Monzogranite	65.3	73.13	Xu, W.C., 2010. Ph.D thesis, China University of Geosciences (Wuhan), p. 1–172
177	T497	92.15	29.71	Granodiorite	67.7	64.12	Xu, W.C., 2010. Ph.D thesis, China University of Geosciences (Wuhan), p. 1–172
178	T498	92.15	29.71	Granodiorite	67.7	64.12	Xu, W.C., 2010. Ph.D thesis, China University of Geosciences (Wuhan), p. 1–172
179	T500	92.15	29.71	Granodiorite	67.7	69.31	Xu, W.C., 2010. Ph.D thesis, China University of Geosciences (Wuhan), p. 1–172
180	T529	94.43	29.45	Granitic gneiss	49.1	73.93	Xu, W.C., 2010. Ph.D thesis, China University of Geosciences (Wuhan), p. 1–172
181	T554	92.20	29.30	Monzogranite	59.7	68.71	Xu, W.C., 2010. Ph.D thesis, China University of Geosciences (Wuhan), p. 1–172
182	T555	92.20	29.30	Monzogranite	59.7	70.02	Xu, W.C., 2010. Ph.D thesis, China University of Geosciences (Wuhan), p. 1–172
183	T556	92.20	29.30	Monzogranite	59.7	69.71	Xu, W.C., 2010. Ph.D thesis, China University of Geosciences (Wuhan), p. 1–172
184	T557	92.20	29.30	Monzogranite	59.7	67.69	Xu, W.C., 2010. Ph.D thesis, China University of Geosciences (Wuhan), p. 1–172
185	T560	93.18	29.26	Fine-grained granite	60.9	70.00	Xu, W.C., 2010. Ph.D thesis, China University of Geosciences (Wuhan), p. 1–172
186	T562	93.18	29.26	Fine-grained granite	60.9	70.05	Xu, W.C., 2010. Ph.D thesis, China University of Geosciences (Wuhan), p. 1–172
187	T672	94.47	29.55	Leucosome	50.3	79.89	Guo et al., 2011, Lithos 127, 54–67
188	T8-15-2	94.01	29.30	Ep granodiorite	63.0	70.46	Zhang et al., 2013, Journal of Petrology 54, 12, 2547–2580
189	T9-30-2	94.35	29.63	Ep granodiorite	63.2	64.41	Zhang et al., 2013, Journal of Petrology 54, 12, 2547–2580
190	T9-33-5	94.44	29.55	Ep granodiorite	64.8	68.42	Zhang et al., 2013, Journal of Petrology 54, 12, 2547–2580
191	T9-33-7	94.44	29.55	Grt two-mica granodiorite	54.9	69.31	Zhang et al., 2013, Journal of Petrology 54, 12, 2547–2580
192	11LS01-1	90.94	29.50	Gabbro	47.3	50.13	Zhu et al., unpublished data
193	11LS06-1	90.71	29.36	Gabbroic diorite	46.2	54.35	Zhu et al., unpublished data
194	11NM01-1	90.27	29.34	Gabbro	46.5	46.90	Zhu et al., unpublished data
195	12AM03-1	86.31	29.64	Granodiorite	53.1	66.45	Zhu et al., unpublished data
196	12AM04-2	86.32	29.63	Gabbro	44.8	52.03	Zhu et al., unpublished data
197	12AM06-2	86.32	29.58	Monzogranite	51.2	73.02	Zhu et al., unpublished data
198	12CZ15-1	86.75	29.75	Monzogranite	60.8	79.14	Zhu et al., unpublished data
199	12DJ01-1	87.71	29.42	Granodiorite	55.1	71.99	Zhu et al., unpublished data
200	12DJ04-1	87.73	29.42	Monzogranite	44.4	74.42	Zhu et al., unpublished data
201	12DJ08-1	87.79	29.44	Granodiorite	51.8	66.09	Zhu et al., unpublished data
202	12DJ11-1	87.87	29.43	Monzogranite	44.5	71.28	Zhu et al., unpublished data
203	12DJ12-1	87.87	29.43	Granodiorite	44.3	66.72	Zhu et al., unpublished data
204	12DJ13-2	87.88	29.43	Gabbro	50.9	50.73	Zhu et al., unpublished data

205	12DJ15-1	87.90	29.43	Granodiorite	51.9	66.54	Zhu et al., unpublished data
206	12DJ17-1	87.99	29.39	Granodiorite	52.1	65.23	Zhu et al., unpublished data
207	12DJC09-3	85.79	29.78	Granodiorite	51.5	67.07	Zhu et al., unpublished data
208	12LY00-1	89.24	29.72	Monzogranite	51.0	77.02	Zhu et al., unpublished data
209	12LY01-1	89.24	29.76	Granodiorite	50.4	67.98	Zhu et al., unpublished data
210	12PC02-2	87.97	29.56	Gabbro	55.0	49.26	Zhu et al., unpublished data
211	12PC12-1	87.99	29.38	Diorite	51.8	56.49	Zhu et al., unpublished data
212	12PC12-2	87.99	29.38	Diorite	51.1	58.60	Zhu et al., unpublished data
213	ST104A	91.81	29.27	Granodiorite	60.1	65.58	Chu et al., 2006, Geology 34, 745–748
214	PIII-17	90.57	29.57		46.1	70.29	Ma et al., 2014, Lithos 196–197, 321–338
215	D0175	90.57	29.57		46.1	73.87	Ma et al., 2014, Lithos 196–197, 321–338
216	PIII-03	90.55	29.52		48.2	72.40	Ma et al., 2014, Lithos 196–197, 321–338
217	PIII-04	90.55	29.52		48.2	70.69	Ma et al., 2014, Lithos 196–197, 321–338
218	PIII-05	90.55	29.52		48.2	71.26	Ma et al., 2014, Lithos 196–197, 321–338
219	PIII-06	90.55	29.52		48.2	71.80	Ma et al., 2014, Lithos 196–197, 321–338
220	PIII-07	90.55	29.52		48.2	72.32	Ma et al., 2014, Lithos 196–197, 321–338
221	PIII-08	90.55	29.52		48.2	69.95	Ma et al., 2014, Lithos 196–197, 321–338
222	PIII-16	90.55	29.52		48.2	68.58	Ma et al., 2014, Lithos 196–197, 321–338
223	D0182	90.55	29.52		48.2	66.37	Ma et al., 2014, Lithos 196–197, 321–338
224	PIII-01	90.60	29.58		48.6	75.95	Ma et al., 2014, Lithos 196–197, 321–338
225	PIII-02	90.60	29.58		48.6	74.51	Ma et al., 2014, Lithos 196–197, 321–338
226		91.81	29.33	Quartz diorite (northern)	56.3	64.55	Chen et al., 2015, Lithos 212–215, 379–396
227		91.81	29.33	Quartz diorite (northern)	56.3	64.09	Chen et al., 2015, Lithos 212–215, 379–396
228		91.80	29.30	Monzogranite	52.4	66.01	Chen et al., 2015, Lithos 212–215, 379–396

Magmatic record of India-Asia collision

Di-Cheng Zhu*, Qing Wang, Zhi-Dan Zhao, Sun-Lin Chung, Peter A. Cawood, Yaoling Niu, Sheng-Ao Liu, Fu-Yuan Wu, and Xuan-Xue Mo

Table S6: Calculation for the timing of collision

The timing of collision is calculated using the following equation similar to the ideas of Leech et al. (2005) and Donaldson et al. (2013), giving the timing as a function of depth of slab breakoff, age of slab breakoff, convergence rate, and slab dip.

$$\textbf{Timing of Collision} = \frac{\text{Depth of Slab Breakoff}}{(\text{Convergence Rate} \times \text{Sin(Slab Dip)})} + \textbf{Age of Slab Breakoff}$$

where, (1) the depth of slab breakoff is assumed to be occurred at 110, 120, 130, 140, and 150 km, given that the exhumed ultrahigh-pressure rocks at ca. 46 Ma (100 km in depth) may not represent the materials from the leading edge of subducted Indian continental margin, (2) the age of slab breakoff is the favored age in this study (ca. 53 Ma), (3) the convergence rates used here are changed from 170 to 150 mm/yr between 67 Ma and 51 Ma (van Hinsbergen et al., 2011), and (4) the slab dip is assumed to be changed from 30°, 45°, and 60° assuming that it increases with depth.

Calculated results indicate that the initial India-Asia collision **was just 1–2 Ma** before the slab breakoff, corresponding to a collision age of ca. 55 Ma.

Table S6 Calculation for the timing of collision

Depth of crust subducted	Convergence rate	Slab dip	Timing of slab breakoff	Initial collision age
100	90	30	53	**55.2**
110	150	30	53	**54.5**
110	160	45	53	**54.0**
110	170	60	53	**53.7**
120	150	30	53	**54.6**
120	160	45	53	**54.1**
120	170	60	53	**53.8**
130	150	30	53	**54.7**

130	160	45	53	**54.1**
130	170	60	53	**53.9**
140	150	30	53	**54.9**
140	160	45	53	**54.2**
140	170	60	53	**54.0**
150	150	30	53	**55.0**
150	160	45	53	**54.3**
150	170	60	53	**54.0**

References:

Donaldson, D.G., Webb. A.A.G., Menold. C.A., Kylander-Clark, A.R.C., Hacker, B.R., 2013. Petrochronology of Himalayan ultrahigh-pressure eclogite. Geology 41, 835–838.

Leech, M.L., Singh, S., Jain, A.K., Klemperer, S.L., Manichavasgam, R.M., 2005. The onset of India-Asia continental collision: Early, steep subduction required by the timing of UHP metamorphism in the western Himalaya. Earth and Planetary Science Letters 234, 83–97.

van Hinsbergen, D.J.J., Steinberger, B., Doubrovine, P., Gassmöller, R., 2011. Acceleration and deceleration of India-Asia convergence since the Cretaceous: Roles of mantle plumes and continental collision. Journal of Geophysical Research 116, B06101, doi: 10.1029/2010JB008051.#

3. References Cited in Tables S3, S4, and S5

Booth, A.L., Zeitler, P.K., Kidd, W.S.F., Wooden, J., Liu, Y.P., Idleman, B., Hren, M., Chamberlain, C.P., 2004. U-Pb zircon constraints on the tectonic evolution of Southeastern Tibet, Namche Barwa area. American Journal of Science 304, 889–929.

Chen, L., Qin, K.Z., Li, G.M., Li, J.X., Xiao, B., Zhao, J.X., Fan, X., 2014. Zircon U–Pb ages, geochemistry and Sr-Nd-Pb-Hf isotopes of Nuri intrusive rocks in Gangdese area, southern Tibet: Constraints on timing, petrogenesis and tectonic transformation. Lithos 212–215, 379–396.

Chen, W., Ma, C.Q., Bian, Q.J., Zhou, R.J., Long, T.C., Yu, S.L., Chen, D.M., Tu, J.H., 2010. Subduction-related late Cretaceous granitoids in Demingding area, east of middle Gangdese, Tibet: Evidences from zircon U-Pb geochronology and geochemistry. Journal of Mineral and Petrology 30, 83–92.

Chu, M.F., Chung, S.L., O'Reilly, S.Y., Pearson, N.J., Wu, F.Y., Li, X.H., Liu, D.Y., Ji, J.Q., Chu, C.H., Lee, H.Y., 2011. India's hidden inputs to Tibetan orogeny revealed by Hf isotopes of Transhimalayan zircons and host rocks. Earth and Planetary Science Letters 307, 479–486.

Chu, M.F., Chung, S.L., Song, B., Liu, D.Y., O'Reilly, S.Y., Pearson, N.J., Ji, J.Q., Wen, D.J., 2006. Zircon U-Pb and Hf isotope constraints on the Mesozoic tectonics and crustal evolution of southern Tibet. Geology 34, 745–748.

Cong, Y., Xiao, K.Y., Zhai, Q.G., Dong, Q.J., 2012. LA-ICP-MS dating and Hf isotopic analysis of zircon from the Puluogang granite in Namulin County, Tibet, and its geological significance. Journal of Jilin University 42, 1783–1795.

Guan, Q., Zhu, D.C., Zhao, Z.D., Dong, G.C., Mo, X.X., Liu, Y.S., Hu, Z.C., Yuan, H.L., 2011. Zircon U-Pb chronology, geochemistry of the Late Cretaceous mafic magmatism in the southern Lhasa terrane and its implications. Acta Petrologica Sinica 27, 2083–2094 (in Chinese with English abstract).

Guan, Q., Zhu, D.C., Zhao, Z.D., Dong, G.C., Zhang, L.L., Li, X.W., Liu, M., Liu, M.H., Mo, X.X., Liu, Y.S., Yuan, H.L., 2012. Crustal thickening prior to 38 Ma in southern Tibet: evidence from lower crust-derived adakitic magmatism in the Gangdese Batholith. Gondwana Research 21, 88–99.

Guan, Q., Zhu, D.C., Zhao, Z.D., Zhang, L.L., Liu, M., Li, X.W., Yu, F., Mo, X.X., 2010. Late Cretaceous adakites in the eastern segment of the Gangdese Belt, southern Tibet: Products

of Neo-Tethyan ridge subduction? Acta Petrologica Sinica 26, 2165–2179 (in Chinese with English abstract).
Guo, L., 2012. The tectono-magma events in the western margin of the eastern Himalayan syntaxis and their geodynamic implications. Ph.D thesis, China University of Geosciences (Wuhan), p. 1–139.
Guo, L., Zhang, H.F., Harris, N., Pan, F.B., Xu, W.C., 2011. Origin and evolution of multi-stage felsic melts in eastern Gangdese belt: Constraints from U-Pb zircon dating and Hf isotopic composition. Lithos 127, 54–67.
Huang, Y., Zhao, Z.D., Zhang, F.Q., Zhu, D.C., Dong, G.C., Mo, X.X., 2010. Geochemistry and implication of the Gangdese batholiths from Renbu and Lhasa areas in southern Gangdese, Tibet. Acta Petrologica Sinica 26, 3131–3142 (in Chinese with English abstract).
Ji, W. Q., Wu, F. Y., Liu, C. Z., Chung, S. L., 2012. Early Eocene crustal thickening in southern Tibet: New age and geochemical constraints from the Gangdese batholith. Journal of Asian Earth Sciences 53, 82–95.
Ji, W.Q., 2010. Geochronology and petrogenesis of granitic rocks from east segment of the Gangdese Batholith, southern Tibet. Ph.D thesis, Chinese Academy of Sciences, p. 1–146.
Ji, W.Q., Wu, F.Y., Chung, S.L., Li, J.X., Liu, C.Z., 2009. Zircon U-Pb chronology and Hf isotopic constraints on the petrogenesis of Gangdese batholiths, southern Tibet. Chemical Geology 262, 229–245.
Jia, L.L., Wang, Q., Zhu, D.C., Chen, Y., Wu, X.Y., Liu, S.A., Zheng, J.P., Zhao, T.P., 2013. Rethinking the geodynamical implications of the basic rocks from Linzhou basin, Tibet. Acta Petrologica Sinica 29, 3671–3680 (in Chinese with English abstract).
Jiang, Z.Q., Wang, Q., Li, Z.X., Wyman, D.A., Tang, G.J., Jia, X.H., Yang, Y.H., 2012. Late Cretaceous (ca. 90 Ma) adakitic intrusive rocks in the Kelu area, Gangdese belt (southern Tibet): Slab melting and implications for Cu–Au mineralization. Journal of Asian Earth Sciences 53, 67–81.
Lee, H.Y., Chung, S.L., Ji, J.Q., Qian, Q., Gallet, S., Lo, C.H., Lee, T.Y., Zhang, Q., 2012. Geochemical and Sr-Nd isotopic constraints on the genesis of the Cenozoic Linzizong volcanic successions, southern Tibet. Journal of Asian Earth Sciences 53, 96–114.
Liu, Y.F., Yang, Z.M., Xie, Y.L., Zhou, P., Du, D.H., Li, Y.X., Li, Q.Y., Qu, H.C., Xu, B., 2012. Zircon SHRIMP U-Pb age and geochemistry of intrusive rocks from Nongruri gold deposit, Gangdese, Tibet. Mineral Deposits 31, 727–744 (in Chinese with English abstract).

Luo, M.C., Wang, L.Q., Leng, Q.F., Chen, W., 2011. Zircon Hf isotope and Ce^{4+}/Ce^{3+} ratio of the monzogranite porphyry and biotite monzonitic granite in Bangpu Mo(Cu) deposit, Tibet. Mineral Deposits 30, 266–278 (in Chinese with English abstract).

Ma, L., Wang, B.D., Jiang, Z.Q., Wang, Q., Li, Z.X., Wyman, D.A., Zhao, S.R., Yang, J.H., Gou, G.N., Guo, H.F., 2014. Petrogenesis of the Early Eocene adakitic rocks in the Napuri area, southern Lhasa: Partial melting of thickened lower crust during slab break-off and implications for crustal thickening in southern Tibet. Lithos 196–197, 321–338.

Ma, L., Wang, Q., Li, Z.X., Wyman, D.A., Jiang. Z.Q., Yang. J.H., Gou, G.N., Guo, H.F., 2013. Early Late Cretaceous (ca. 93 Ma) norites and hornblendites in the Milin area, eastern Gangdese: Lithosphere–asthenosphere interaction during slab roll-back and an insight into early Late Cretaceous (ca. 100–80 Ma) magmatic "flare-up" in southern Lhasa (Tibet). Lithos 172–173, 17–30.

Ma, L., Wang, Q., Wyman, D.A., Jiang, Z.Q., Yang, J.H., Li, Q.L., Gou, G.N., Guo, H.F., 2013. Late Cretaceous crustal growth in the Gangdese area, southern Tibet: Petrological and Sr–Nd–Hf–O isotopic evidence from Zhengga diorite–gabbro. Chemical Geology 349–350, 54–70.

Ma, L., Wang, Q., Wyman, D.A., Li, Z.X., Jiang, Z.Q., Yang, J.H., Gou, G.N., Guo, H.F., 2013. Late Cretaceous (100–89 Ma) magnesian charnockites with adakitic affinities in the Milin area, eastern Gangdese: Partial melting of subducted oceanic crust and implications for crustal growth in southern Tibet. Lithos 175–176, 315–332.

Mo, X.X., Niu, Y.L., Dong, G.C., Zhao, Z.D., Hou, Z.Q., Zhou, S., Ke, S., 2008. Contribution of syncollisional felsic magmatism to continental crust growth: A case study of the Paleocene Linzizong Volcanic Succession in southern Tibet. Chemical Geology 250, 49–67.

Quidelleur, X., Grove, M., Lovera, O.M., Harrison, T.M., Yin, A., Ryerson, F.J., 1997. Thermal evolution and slip history of the Renbu Zedong Thrust, southeastern Tibet. Journal of Geophysical Research 102(B2): 2659–2679.

Wang, B.D., Wang, L.Q., Li, B., Huang, H.X., Chen, F.Q., Duan, Z.M., Zeng, Q.G., 2012. Geochronology and petrogenesis of the ore-bearing pluton in Chagele deposit in middle of the Gangdese metallogenic belt. Acta Petrologica Sinica 28, 1647−1662.

Wang, L., Zeng, L.S., Gao, L.E., Chen, Z.Y.m 2013. Early Cretaceous high Mg# and high Sr/Y clinopyroxene-bearing diorite in the southeast Gangdese batholith, southern Tibet. Acta Petrologica Sinica 29, 1977−1994.

Watson, E.B., Harrison, T.M., 1983. Zircon saturation revisited: temperature and composition effects in a variety of crustal magma types. Earth and Planetary Science Letters 64, 295–304.

Wen, D.R., Chung, S.L., Song, B., Iizuka, Y., Yang, H.J., Ji, J., Liu, D., Gallet, S., 2008b. Late Cretaceous Gangdese intrusions of adakitic geochemical characteristics, SE Tibet: Petrogenesis and tectonic implications. Lithos 105, 1–11.

Wen, D.R., Liu, D.Y., Chung, S.L., Chu, M.F., Ji, J.Q., Zhang, Q., Song, B., Lee, T.Y., Yeh, M.W., Lo, C.H., 2008. Zircon SHRIMP U–Pb ages of the Gangdese batholith and implications for Neotethyan subduction in southern Tibet. Chemical Geology 252, 191–201.

Wen, D.W., 2007. The Gangdese Batholith, southern Tibet: Ages, geochemical characteristics and petrogenesis. Ph.D thesis, National Taiwan University, p. 1−120.

Xu, W.C., 2010. Spatial variation of zircon U-Pb ages and Hf isotopic compositions of the Gangdese granitoids and its geologic implications. Ph.D thesis, China University of Geosciences (Wuhan), p. 1−172.

Yue, Y.H., Ding, L., 2006. $^{40}Ar/^{39}Ar$ Geochronology, geochemical characteristics and genesis of the Linzhou basic dikes, Tibet. Acta Petrologica Sinica, 22(4): 855–866 (in Chinese with English abstract).

Zhang, Z.M., Dong, X., Xiang, H., He, Z.Y., Liou, J.G., 2014. Metagabbros of the Gangdese arc root, south Tibet: Implications for the growth of continental crust. Geochimica et Cosmochimica Acta, doi: 10.1016/j.gca.2014.01.045.

Zhang, Z.M., Dong, X., Xiang, H., Liou, J.G., Santosh, M., 2013. Building of the Deep Gangdese Arc, South Tibet: Paleocene Plutonism and Granulite-Facies Metamorphism. Journal of Petrology 54, 2547−2580.

Zhang, Z.M., Zhao, G.C., Santosh, M., Wang, J.L., Dong, X., Shen, K., 2010. Late Cretaceous charnockite with adakitic affinities from the Gangdese batholith, southeastern Tibet: Evidence for Neo-Tethyan mid-ocean ridge subduction? Gondwana Research 17, 615–631.

Zhu, D.C., Zhao, Z.D., Niu, Y.L., Dilek, Y., Hou, Z.Q., Mo, X.X., 2013. The origin and pre-Cenozoic evolution of the Tibetan Plateau. Gondwana Research 23, 1429–1454.

Zhu, D.C., Zhao, Z.D., Niu, Y.L., Mo, X.X., Chung, S.L., Hou, Z.Q., Wang, L.Q., Wu, F.Y., 2011. The Lhasa Terrane: Record of a microcontinent and its histories of drift and growth. Earth and Planetary Science Letters 301, 241–255.

#
